# Comprehensive Transcriptome Analysis of mRNA Expression Patterns of Early Embryo Development in Goat under Hypoxic and Normoxic Conditions

**DOI:** 10.3390/biology10050381

**Published:** 2021-04-28

**Authors:** Yongjie Wan, Dongxu Li, Mingtian Deng, Zifei Liu, Liang Liu, Feng Wang

**Affiliations:** Jiangsu Livestock Embryo Engineering Laboratory, College of Animal Science and Technology, Nanjing Agricultural University, Nanjing 210095, China; 2019105030@njau.edu.cn (D.L.); mtdeng@njau.edu.cn (M.D.); 2019205002@njau.edu.cn (Z.L.); 2020105029@stu.njau.edu.cn (L.L.); caeet@njau.edu.cn (F.W.)

**Keywords:** goat, embryo development, normoxia, hypoxia, RNA-seq, DEGs, oxidative stress

## Abstract

**Simple Summary:**

Oxygen plays a vital role in the development of early embryos, no matter whether it is too high or low, it will adversely affect the early embryo development, but the mechanisms involved in these effects are still unclear. RNA-seq was performed to compare 8-cell-stage and blastocyst-stage goat embryos under hypoxic and normoxic conditions, the mRNA expression mechanisms of 8-cell- and blastocyst-stage embryos were systematically analyzed under hypoxic and normoxic conditions. Functional enrichment analysis indicated that these differentially expressed genes (DEGs) were mainly related to biological processes and function regulation. In conclusion, we can infer that oxidative stress regulates early embryo development by affecting the expression of zygotic genes and transcription factors, and those stress genes play a potential role in adaptation to normoxic environments in goat embryos.

**Abstract:**

It has been reported that hypoxic environments were more suitable for the in vitro development of mammalian embryos, but the underlying mechanisms were still unclear. In the present study, RNA-seq was performed to compare 8-cell-stage and blastocyst-stage goat embryos under hypoxic and normoxic conditions; zygotes were checked at 72 and 168 h to 8-cell stage (L8C) and blastocyst stage (LM) in hypoxic conditions and 8-cell stage (H8C) and blastocyst stage (HM) in normoxic conditions. In the H8C and L8C groups, 399 DEGs were identified, including 348 up- and 51 down-regulated DEGs. In the HM and LM groups, 1710 DEGs were identified, including 1516 up- and 194 down-regulated DEGs. The expression levels of zygotic genes, transcription factors, and maternal genes, such as *WEE2, GDF9, HSP70.1, BTG4,* and *UBE2S* showed significant changes. Functional enrichment analysis indicated that these DEGs were mainly related to biological processes and function regulation. In addition, combined with the pathway–gene interaction network and protein–protein interaction network, twenty-two of the hub genes were identified and they are mainly involved in energy metabolism, immune stress response, cell cycle, receptor binding, and signal transduction pathways. The present study provides comprehensive insights into the effects of oxidative stress on early embryo development in goats.

## 1. Introduction

Goats are widely distributed and diverse domestic animals because of their high social value and short gestation period; they have become an important species in current biological research and applications [1]. Assisted reproductive technologies such as superovulation, in vitro fertilization, in vitro embryo culture, and embryo transfer have been widely promoted and applied in goats and sheep, and they play a significant role in improving livestock productivity [2]. In vitro embryo culture has always been the top priority of our research; it directly determines the development of the individual. In addition, the study of goat embryos in vitro has laid the foundation for human-assisted reproductive technology, which is of great significance for the promotion of biological research.

Oxidative stress refers to a state in which the oxidative and antioxidative effects in the body are unbalanced, leading to excessive reactive oxygen species (ROS) accumulation; oxidative stress participates in a variety of key biological processes [3,4,5]. Recent reports suggested that the in vitro culture of embryos significantly increases the accumulation of ROS in embryonic cells, and the harmful effects of oxidative stress on embryonic development are caused by the excessive increase of ROS [6]. Compared with embryos developed in vivo, embryonic cells developed in vitro were more prone to apoptosis [7]. It has been reported that apoptosis of embryos cultured in vitro occurs at a specific stage, which may be caused by the influence of certain genes on a certain pathway, for example, apoptosis occurs in human 2–8-cell embryos [8], mice 1-cell embryos [9], and cattle 8–16-cell embryos [10]. In early embryos of mice, it was found that high oxygen concentration in the culture environment would accumulate a large amount of ROS and easily increase the incidence of apoptosis, while the hypoxic environment was more suitable for the in vitro development of mammalian embryos [11].

The culture environment of cells is the crucial factor that induces cell apoptosis and leads to embryonic development stagnation. Oxygen plays an important role in in vitro embryo culture, and the oxygen concentration directly affects the embryo development rate and quality [12]. It has been shown that early embryo culture can develop normally under atmospheric oxygen concentrations [13]. However, studies have found that the oxygen concentration in body tissues was generally 2–8% of the volume fraction in the hypoxic environment, and the fallopian tubes of most mammals were a hypoxic environment [14,15]. Studies have shown that reducing the oxygen concentration in the incubator was more conducive to embryo development in humans [16], cattle [17], mice [18], and swine [19]. In subsequent studies, 5% oxygen concentrations (hypoxic) were also used in vitro, resulting in a higher blastocyst rate and greater benefits before and after implantation compared with those of 20% oxygen concentrations (normoxia) [20].

In this study, we aimed to identify critical functional genes and pathways involved in goat embryos the oxidative stress adaptation. The mRNA expression mechanisms of 8-cell- and blastocyst-stage embryos were systematically analyzed under hypoxic and normoxic conditions. The analysis of these data will lay a preliminary foundation for further research on the biological mechanism of oxidative stress on embryonic development.

## 2. Materials and Methods

The goats were supplied by the Jinsheng Goat Breeding Technology Development Co, Ltd. Haimen City, Jiangsu Province, China. The trial was conducted at the Goat R&D Center in Haimen City, Jiangsu Province in December 2019. All biological reagents were sourced from Sigma-Aldrich (St. Louis, MO, USA), unless noted otherwise. This experiment was approved by the Animal Care (SYXK2011-0036) and Use Committee of Nanjing Agricultural University, China.

### 2.1. Collection and Culture of Embryos

Collection and culture of embryos was completed with reference to previous research methods [21]. Briefly, eight-month-old female goats were estrus synchronized, superovulated, and then mated with 2-year-old bucks. Progesterone sponges were intravaginally implanted in goats for 11 days, followed by the administration of 100 IU PG at the time of sponge removal, then donors were mated at 36 h and 48 h after sponge removal. The zygotes of goats within 24 h of mating were removed from the fallopian tubes by surgical method, washed three times with the embryo culture medium in vitro, divided into two groups, and transferred into the pre-balanced BO-IVC medium (IVC Bioscience, Falmouth, United Kingdom) supplemented with 10% fetal bovine se-rum (FBS). They were cultured at the 38.5 °C at a saturated humidity and at different gas composition conditions (5% CO_2_, 5% O_2_, 90% N_2_ and 5% CO_2_, 20% O_2_, 75% N_2_,). The one-cell embryos were cultured and divided into the hypoxia group and the normoxia group, which were checked at 72 and 168 h to 8-cells stage (L8C) and blastocyst stage (LM) under hypoxia and to 8-cell stage (H8C) and blastocyst stage (HM) under normoxia. Five 8-cell-stage or blastocyst-stage embryos were randomly absorbed by the mouth pipette as a sample, each group had 3 replicates, which were directly lysed for RNA sequencing.

### 2.2. RNA Library Construction and Sequencing

The Smart-seq2 method was used for cDNA synthesis as in a previous study [22]. The initiation and terminal connectors of the cDNA sequence were employed as primers for PCR amplification, and the obtained double-stranded cDNA was sufficient for library construction and sequencing. Incubation was performed with the fragmented cDNA using dsDNA fragmentase (NEB, M0348S) at 37 °C for 30 min. The fragmented cDNA at the size of 150–300 bp was screened by the provided sample purification beads for library construction. Illumina Novaseq™ 6000 was used for sequencing after the library was qualified; the sequencing read length was 2 × 150 bp.

### 2.3. Read Alignment and Differential Expression Analysis

The sequencing data were aligned to goat genome ARS1 using HISAT2 (version 2.0) with default parameters. SAMtools (version 0.19) was used to sort and index the reads, and only reads with a unique mapping location in the genome were retained for further analysis. Using Htseq with product count data for each transcript, the expression of genes was normalized to fragments per kilobase of exon model per million mapped reads (FPKM). EdgeR was used for differential analysis of StringTie (version 1.3.0) assembled and quantified genes, the threshold of significant difference was|log2 foldchange|≥ 1, *p* < 0.05). Graphic displays of the results of the differential expression were created using R software (version 3.2.5).

### 2.4. Functional Annotation of Differentially Expressed Genes

GO function and KEGG pathway enrichment analyses were performed with reference to previous studies [23]. GO and KEGG terms with FDR adjusted *p* < 0.05 were considered statistically significant.

### 2.5. Whole Transcriptome Amplification of Single-Cell Embryo and Quantitative Real-Time PCR

Single cell whole transcriptome amplification was performed according to the instructions of the CellAmp Whole Transcriptome Amplification Kit (Takara, Dalian, China). In brief, after putting 8-cell- or blastocyst-stage embryos directly into the lysate buffer to completely lyse, we add edMgCl_2_ and RT Enzyme Mix to synthesize the first strand of cDNA. Then, dATP and TdT enzymes were added to the above reaction solution for an A tail reaction. Second-strand cDNA was synthesized by combining dNTP mix and *Ex TaqHot* Start, and the cDNA was amplified using *Ex TaqHot*, which was diluted for subsequent experiments.

Quantitative Real-Time PCR (qRT-PCR) was implemented using a previous researched method [24]. The relative mRNA expression levels of target genes were calculated using the comparative 2^−ΔΔCt^ method after normalization to *GAPDH* control. The PCR primers were designed using NCBI Primer Blast online software, synthesized by Tsingke (Nanjing, China), and are shown in Table S1.

### 2.6. Cell Staining

The Hoechst staining method was performed according to previous research [25]. Embryos were washed thrice with phosphate-buffered saline (PBS) containing 0.3% polyvinyl pyrrolidone and then fixed in 4% paraformaldehyde for 1 h, washed thrice again and transferred to 0.5% TritonX-100 for 20 min. After blockade with 1% bovine serum albumin for 1 h at room temperature they were treated with Hoechst 33,342 for 10 min. Finally, embryos were mounted on glass slides and observed under a confocal laser scanning microscope (LSM 700 META; Zeiss, Jena, Germany).

### 2.7. Statistical and Data Analysis

The data for cell counts were normalized by arcsine transformation and tested by Wilcoxon test. The data obtained for cleavage rate, blastocyst rate, and qRT-PCR were statistically analyzed by using the Student’s *t*-test. All the statistical analysis was performed using SPSS software (version 24.0, SPSS) and * indicates a significant difference (*p* < 0.05), while ** means the difference was extremely significant (*p* < 0.01). The data were expressed as mean ± SE.

## 3. Results

### 3.1. Effects of Oxygen Concentration on Embryonic Growth and Development

**Figure 1:**
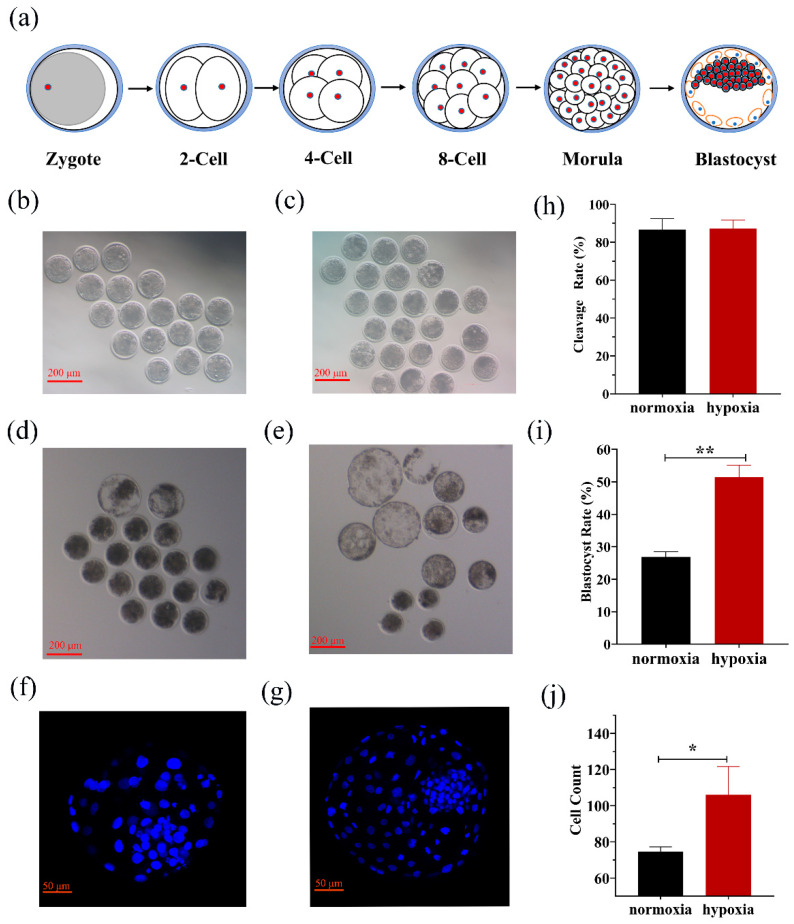
The stages of early embryonic development. (**a**) Six key stages of embryonic development including zygote, 2-cell, 4-cell, 8-cell, morula, and blastocyst stages. Fertilized embryos were cultured to the 8-cell stage (**b**) and blastocyst stage (**d**) in normoxic conditions and to the 8-cell stage (**c**) and blastocyst stage (**e**) in hypoxic conditions. The images (**f**,**g**) were captured with a microscope (LSM 700 META; Zeiss, Jena, Germany). The cleavage rate (**j**) was not significantly different under hypoxic conditions compared to that of normoxic conditions, but the blastocyst rate (**h**) and cell count (**i**) were significantly increased compared to those in the normoxic conditions. Embryonic development data were collected under hypoxic (n = 71) and normoxic (n = 60) conditions. * *p* < 0.05, ** *p* < 0.01.

As shown in Figure 1, the zygotes were checked at 72 and 168 h at the 8-cell stage and blastocyst stage, respectively. The cell counts were calculated by staining the blastocysts under normoxic and hypoxic conditions with Hoechst 33,342. The results showed that the cleavage rates (87.30 ± 4.39 vs. 86.67 ± 5.77) were not significantly different under hypoxic conditions compared to normoxic conditions. However, hypoxia significantly (*p* < 0.05) improved the blastocyst rate (51.44 ± 3.67 vs. 26.85 ± 1.60) and increased the cell count (106 ± 10.44 vs. 74.67 ± 2.52) as compared to normoxia.

### 3.2. Summary of Transcriptomic Profiles of Goat Embryos

**Figure 2:**
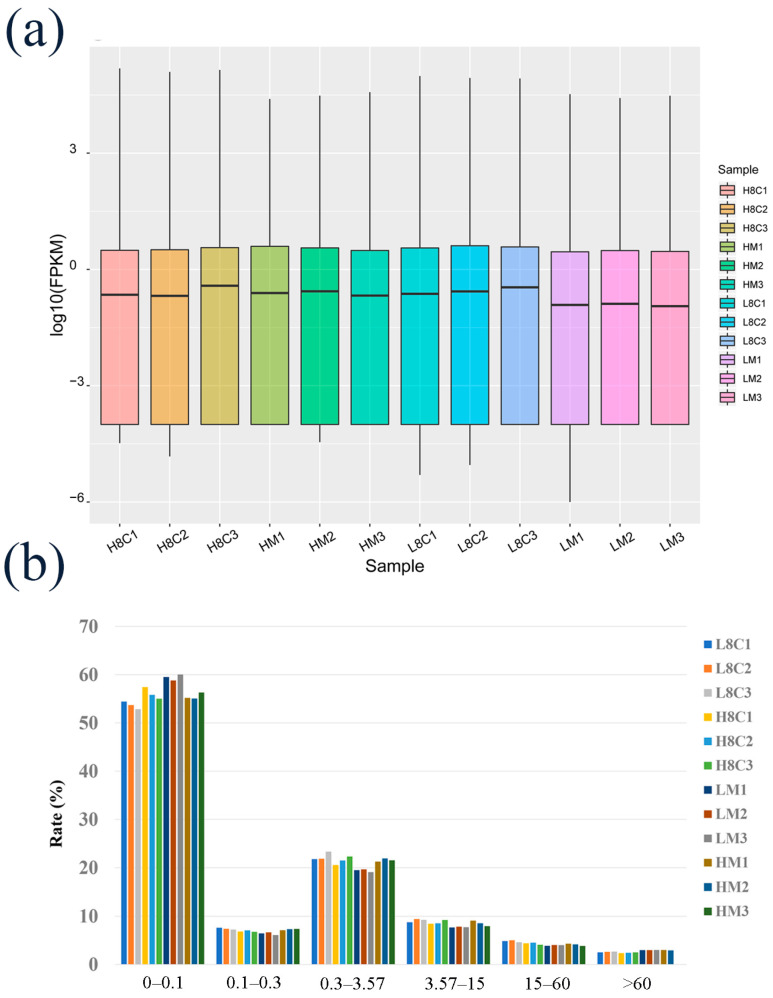
Genomic information on mRNA in goat embryos. (**a**) Distributions of expression values of twelve samples. The box-and-whisker plots show log10 (FPKM) of each gene from the twelve sets of RNA-Seq data. The black line in the box represents the median. (**b**) The number of detected genes with different expression levels against the range of fragments per kilobase of exon length million mapped reads (FPKM) values. Zygotes were cultured to 8-cell stage (L8C) and blastocyst stage (LM) in hypoxic conditions and to 8-cell stage (H8C) and blastocyst stage (HM) in normoxic conditions.

Our RNA sequencing data generated a total of 75.37 GB of clean data, in which 84.26–89.59% were mapped to the goat reference genome, and about 60% of the reads were uniquely mapped to the genomes in the mapped reads (Table S2). In the reference genome, alignment region distributions of 88.87–93.58% were located in annotated exons and 5.13–7.86% in introns (Figure S1). The known annotation information in the goat database was counted (Figure S2). The average fragments per kilobase of transcript per million (FPKM) values of expressed gene distribution were similar among the twelve samples (Figure 2a), and the FPKM intervals of each sample were largely between 0–0.1 and 0.3–3.57 (Figure 2b).

### 3.3. Identification and Functional Analysis of DEGs in 8-Cell-Stage Goat Embryo

**Figure 3:**
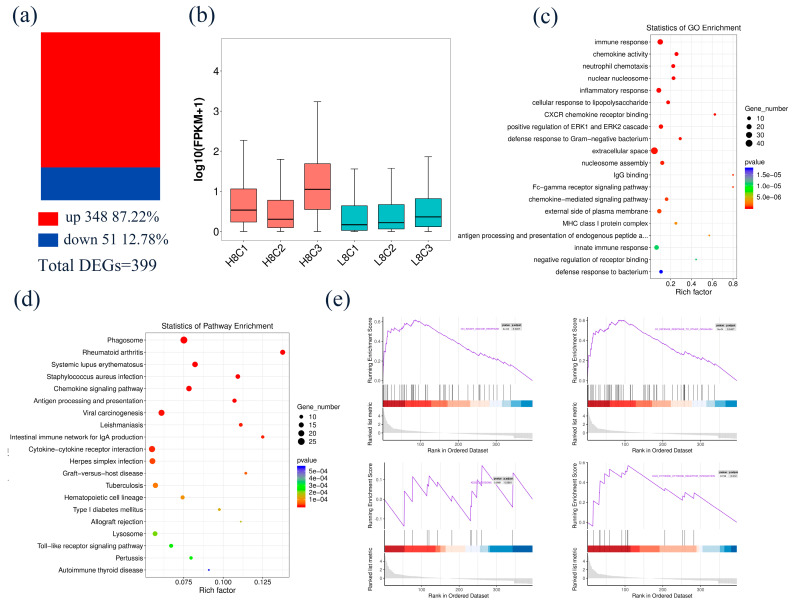
Identification and functional analysis of DEGs in 8-cell-stage embryo. (**a**) Comparing H8C with L8C, a total of 399 DEGs were identified, including 348 up-regulated and 51 down-regulated DEGs. (**b**) Boxplot of FPKM between H8C and L8C. (**c**,**d**) The GO and KEGG enrichment analyses of the DEGs. (**e**) The DEGs were enriched in cytokine–cytokine receptor interactions, lysosomes, innate immune responses, and defense response to Gram-negative bacterium.

Comparing H8C with L8C, a total of 399 DEGs were identified, including 348 up-regulated and 51 down-regulated DEGs (Figure 3a, Table S3). The average FPKM of the DEGs in each sample in H8C was significantly increased compared to that of the samples of L8C (Figure 3b). To further study the role of these DEGs in 8-cell- and blastocyst-stage embryos under oxidative stress, GO and KEGG analyses were implemented to evaluate the function of the DEGs. The DEGs were enriched in GO terms including immune response, neutrophil chemotaxis, nuclear nucleosome, and chemokine activity (Figure 3c, Table S4). KEGG pathway enrichment analysis mainly contained the phagosome, toll-like receptor signaling pathway, cytokine–cytokine receptor interaction, and chemokine signaling pathway, (Figure 3d, Table S5). Similar results were found in the GSEA (Figure 3e).

### 3.4. Identification and Functional Analysis of DEGs in Blastocyst-Stage Goat Embryo

**Figure 4:**
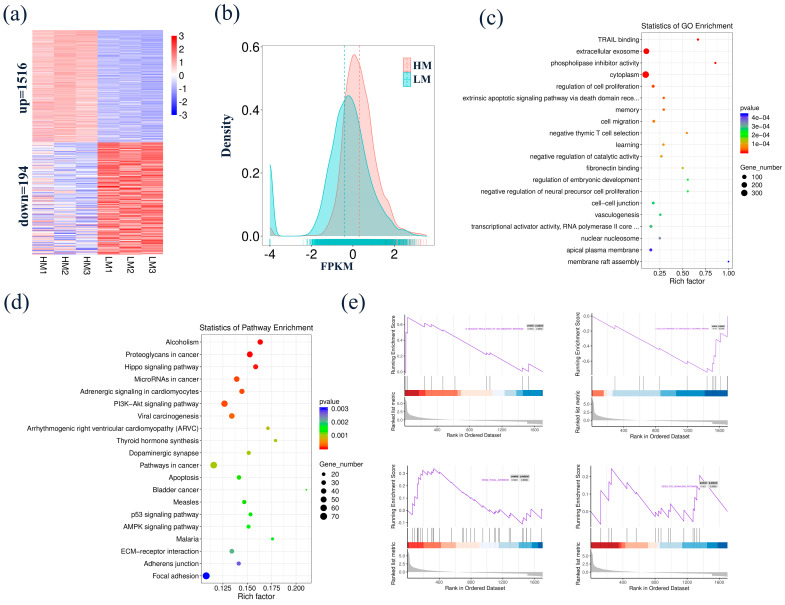
Identification and functional analysis of DEGs in blastocyst-stage embryo. (**a**) Comparing HM with LM, a total of 1710 DEGs were identified, including 1515 up-regulated and 194 down-regulated DEGs. (**b**) Density plot of FPKM between HM and LM. (**c**,**d**) The GO and KEGG enrichment analyses of the DEGs. (**e**) The DEGs were enriched in negative thymic T cell selection, p53 signaling pathway, and focal adhesion.

Comparing HM with LM, a total of 1710 DEGs were identified, including 1515 up-regulated and 194 down-regulated DEGs (Figure 4a, Table S6). The average FPKM of the DEGs in each sample in HM was significantly increased compared to that of the samples of LM (Figure 4b). To further study the role of these DEGs in blastocyst-stage embryos under oxidative stress, GO and KEGG analyses were implemented to evaluate the function of the DEGs. GO enrichment analysis indicated that the DEGs were enriched in the regulation of cell proliferation; extracellular exosome, cytoplasm, and phospholipase inhibitor activity; and other correlated biological processes (Figure 4c, Table S7). The KEGG pathway mainly included the Hippo signaling pathway, thyroid hormone synthesis, PI3K-Akt signaling pathway, and apoptosis (Figure 4d, Table S8). Similar results were found in the GSEA (Figure 4e).

### 3.5. Identification and Functional Analysis of Common DEGs between H8C and L8C Groups and HM and LM Groups

**Figure 5:**
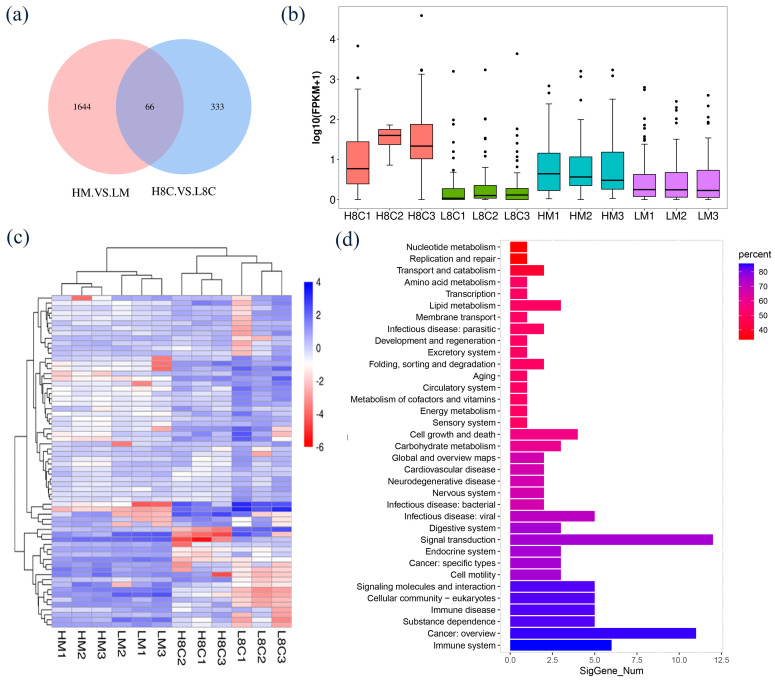
Hub gene identification and DEGs’ protein–protein interaction analysis. (**a**) Venn diagram of DEGs between the H8C and L8C embryos, and between the HM and LM embryos. (**b**) Boxplot of FPKM in the 8-cell stage and blastocyst stage embryos. Heatmap (**c**) and KEGG enrichment analysis (**d**) of 66 DEGs.

A total of 66 DEGs were identified in both groups of embryos (Figure 5a, Table S9). The average FPKM in each group of embryos under normoxia was significantly increased when compared to those under hypoxia (Figure 5b). The cluster analysis of 66 DEGs is shown in the Figure 5c. KEGG enrichment analysis was implemented and revealed that these DEGs were principally enriched in pathways related to cellular processes; environ-mental information processing; genetic information processes; human diseases; metabolism and organismal systems, such as cellular community, signal molecules, and interaction; folding, sorting, and degradation; immune disease; nucleotide metabolism; and immune system (Figure 5d, Table S10).

### 3.6. Analysis of the Protein–Protein Interaction (PPI) and Pathway–Gene Interaction Networks of DEGs

In HM and LM groups, the string online database and Cytoscape (version 3.7.2) tool were utilized to establish the PPI network, which was made up of 184 nodes (FPKM ≥ 2 and fold change ≥ 2) and 265 edges (Figure S3a, Table S11). The MOCODE tool was used to screen the most important modules in the PPI network, including 6 nodes and 14 edges (Figure S3b), such as *PLK2, WEE1, CKS1B, CCNA1, CDC25B,* and *CDKN1A*. KEGG enrichment analysis of the DEGs in these modules associated them with the FOXO signaling pathway, cell cycle, P53 signaling pathway, and PI3K-Akt signaling pathway (Table S12).

**Figure 6:**
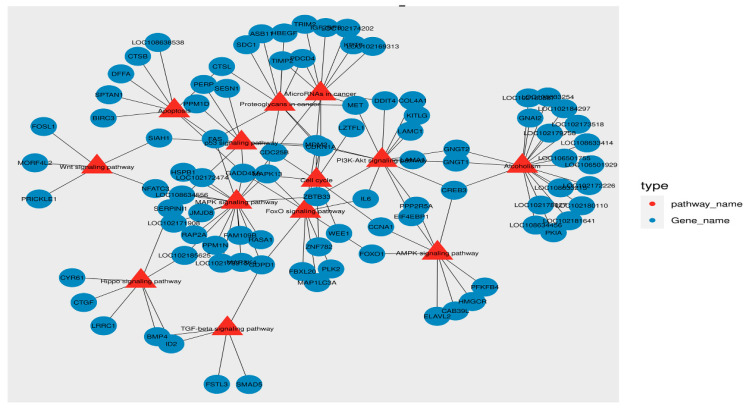
The pathway–gene interaction network diagram was established using ten KEGG pathways and 113 DEGs; the red is the pathway and the green is the gene name.

The pathway–gene interaction network diagram was established to further analyze the effects of different oxygen concentrations on embryonic development. In HM and LM groups, ten KEGG pathways associated with oxidative stress were screened, and a total of 113 DEGs (FPKM > 1) participated in these pathways, the main genes involved in these pathways were *FAS, GAPD1, GADD45A, ZBTB33, CREB3, MDM2, CDKN1A, CDC25B,* and *IL6*. (Figure 6). In addition, seven DEGs (*HSP70.1, HSPB1, FOXO1, FABP3, PPP1R1A, GPX6, MAPK13*) were related to energy metabolism, stress responses, and metabolism. The enriched KEGG mainly contained the AMPK signaling pathway, FOXO signaling pathway, and Rap1 signaling pathway. In summary, the analysis results showed that sixteen DEGs were identified to play a potential role in adaptation to normoxic environments in goat embryos (Table S12).

### 3.7. Key Genes Associated with Oxidative Stress Affecting Embryonic Development and Validation of Sequencing Data

**Figure 7:**
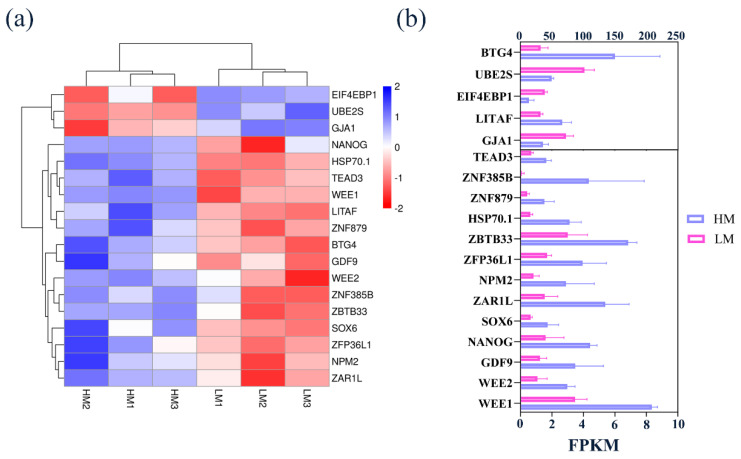
Analysis of DEGs that regulate embryonic development and validation of sequencing results. (**a**) Heatmap analysis of the 18 DEGs (zygotic genes, transcription factors, and maternal genes). (**b**) The histogram of FPKM of zygotic genes, transcription factors, and maternal genes.

The normal development of an embryo depends on the activation of zygotic genome activation (ZGA), and transcription factors are involved in regulating the activation of ZGA. Eighteen DEGs related to embryonic development were screened and used for heat map analysis in HM and LM (Figure 7a). As shown in Figure 7b, the expression levels of zygotic genes, *BTG4* and *HSP70.1*, transcription factors *TEAD3, ZNF596, ZNF879, ZNF385B, ZAR1L, NPM2, ZFP36L1, SOX6, LITAF, NANOG,* and *ZBTB33,* and maternal genes *WEE1, WEE2,* and *GDF9* were significantly increased, while the expression levels of zygotic genes *UBE2S* and *EIF4EBP1* were significantly decreased. Oxidative stress may affect early embryo development by regulating the expression of these genes.

To further assess the reliability of RNA sequencing, *CKS1B, ALDH1A1, UBB, SUMO2,* and *OXT* were randomly selected by qRT–PCR to validate the relative expression level of sequencing in the embryo. The relative expression level of qRT–PCR had the same trend as that of RNA–seq, which showed that sequencing data were efficient and reliable (Figure S4).

## 4. Discussion

This experiment showed that different oxygen concentrations had significant effects on embryo development and identified the key genes and pathways involved in oxidative stress, which further revealed the regulatory mechanism of oxidative stress. It lays a preliminary foundation for us to further study the influence of culture environment on early embryo development.

ZGA is the genome-wide gene activation in the zygote, which plays an extremely critical role in early embryonic development [26]. In this experiment, 8-cell-stage and blastocyst-stage embryos were selected for sequencing analysis to explore the effects of different oxygen concentrations on the development of early goat embryos. Some studies have shown that the ZGA period of goats and sheep both occurs in the 8-cell stage [21,27]. During the ZGA period, maternal mRNA and protein were gradually eliminated, and zygotic genes were activated to participate in the regulation of early embryonic development [28].

We found that the number of DEGs in the HM and LM groups was far higher than that in the H8C and L8C groups. A large number of transcription factors were activated after the early embryonic ZGA occur, this result may be related to the ZGA period of goat embryos starting at the 8-cell stage. In addition, we analyzed the difference in the rate of blastocysts under different oxygen concentrations, and the results showed that the expression levels of maternal genes, zygotic genes, and transcription factors were significantly different in the two groups. These genes play an essential role in early embryonic development. Studies have shown that the maternal gene *WEE1* was essential for maintaining genomic integrity in mammals; lack of *WEE1* can lead to DNA damage and chromosome aneuploidy, which plays an important role in the regulation of early embryonic development [29]. Previous studies found that the transcription level of *GDF9* was also different in various stages of embryonic development [30,31]. In mouse oocytes, *BTG4* plays the role of the meiotic cell cycle coupling maternal-to-zygotic transition (MZT) permitting factors, while females lacking *BTG4* will produce other morphologically normal oocytes, but they are unable to reproduce due to early developmental arrest [32]. As a new effector of *SOX2* protein degradation, *UBE2S* regulates the stability of *SOX2* and participates in the maintenance of embryonic stem cell pluripotency [33]. The synthesis of *NPM2* maternal mRNA was a necessary condition for the activation of the zygotic genome and is essential for normal development after the blastocyst stage. *NPM2* knockout mouse embryos rarely develop to the 2-cell stage due to the lack of complexes necessary for transcription [34]. *NANOG* as a transcription factor that is necessary for the self-renewal of embryonic stem cells and maintenance of pluripotency. The number of vegetative ectoderms and the total number of blastocysts in embryos is reduced with *NANOG* expression [35]. According to the above, the difference in blastocyst rates between the two groups may be closely related to the differential expression of these genes. The differential expression of zygote genes, maternal genes, and transcription factors was involved in regulating the development of early embryos.

It can be seen from the experiment results that the blastocyst rate of early embryos cultured under hypoxia were greater than those cultured under normoxia, suggesting that the number of arrested embryos under normoxic conditions was more than that of hypoxic conditions after cleavage, so we can infer that differences in embryo development were caused by different oxygen concentrations, and hypoxic culture was more conducive to embryo development. Studies have shown that embryos cultured under normoxia can accelerate the formation of ROS in the environment and produce more ROS than embryos cultured under hypoxia [36,37]. Our results were consistent with previous studies that showed that excessive ROS can affect maternal-to-zygotic transition and embryonic development retardation [38]. It also has been proven that hypoxia was more conducive to improving early embryonic development [39,40]. In addition, the effect of OS on the in vitro culture of sheep embryos has also been reported; L-carnitine supplementation during in vitro maturation reduces oxidative stress-induced embryo toxicity by decreasing intracellular ROS that in turn alters transcript levels of antioxidant enzymes and improves developmental potential of oocytes and embryos [41]. By studying the molecular mechanism of OS in embryo development, we are committed to exploring the suitable culture environment for embryo development, which has a positive significance for promoting the development of in vitro-assisted reproductive technology.

Previous studies have found that oxidative stress significantly alters the gene expression of a variety of cells to adapt to the culture environment [42]. In HM and LM groups, a total of 1710 differential genes were screened and identified: *FOXO1, GPX6, GADD45A, HSP70.1* were screened as hub genes and it has been confirmed that these genes were related to the regulation of oxidative stress [43,44,45,46]. Previous studies have shown that the silencing of *FABP3* can cause an excessive production of intracellular ROS and lead to mitochondrial dysfunction [47]. Functional enrichment analysis demonstrated that these DEGs were mainly related to biological processes and function regulation. For example, *CDC25B, CCNA1, WEE1, PLK2,* and *CKS1B* are related to cell cycle [48,49,50,51,52], while *HSPB1, ZBTB33, BMP4,* and *PDGFA* are involved in regulating embryonic growth and development [53,54,55,56].

Through the analysis of the enrichment of 8-cell stage differential genes using GO and KEGG under different oxygen concentrations, we found that the effect of ROS on 8-cell-stage embryos was mainly enriched in the biological process of immune response, inflammatory response, and disease occurrence. Previous studies have shown that oxidative stress can cause inflammation and immune responses in cells [57]. When the embryo developed to the blastocyst stage through zygotic genome activation, the main effect of ROS on the blastocyst-stage embryo was to promote angiogenesis, energy metabolism, hormone synthesis, cell apoptosis, and other biological processes, similar results were obtained in porcine granulosa cells undergoing oxidative studies [58]. A total of 66 DEGs were identified in both groups of embryos; the expression of these DEGs was not related to the stage of the embryo, but was caused by the different culture environment, which shows that oxidative stress is involved in the regulation of early embryonic development. We can infer that both 8-cell-stage embryos and blastocyst-stage embryos adapt to the culture environment by regulating organic defense mechanisms in response to oxidative stress.

Studies have shown that multiple signaling pathways were induced when oxidative stress regulated cellular biological processes, such as Wnt [59], TGF-β [3], FOXO [60], Hippo [61], PI3K-Akt [62], and MAPK signaling pathways [63]. In this experiment, the AMPK signaling pathway, p53 signaling pathway, and Rap1 signaling pathway were also highly enriched in the process of oxidative stress in the early development of goat embryos. Based on the above results, we hypothesize that goat embryos may adapt to oxidative stress through energy metabolism, immune stress response, cell cycle changes, receptor binding, signal transduction pathways, and expression of stress genes.

## 5. Conclusions

In summary, differentially expressed RNA profiles were constructed by RNA-seq technology in this experiment and demonstrated that 8-cell-stage- and blastocyst-stage embryos in goats have significant changes in gene expression under different oxygen concentrations. Oxidative stress regulates early embryo development by affecting the expression of zygotic genes and transcription factors, and those stress genes play a potential role in adaptation to normoxic environments in goat embryos. These results provide new insights for further understanding of the regulation mechanism of oxidative response in early embryo development of goats.

## Data Availability

The data used to support the findings of this study are included within the article and Supplementary Materials.

## Supplementary Materials

The following are available online at https://www.mdpi.com/article/10.3390/biology10050381/s1, Figure S1. The reference genome alignment region distribution; Figure S2. The known annotation information in the species database was counted; Figure S3. The PPI network established using 132 up-regulated DEGs; Figure S4. Validation of sequencing data. Table S1. The PCR primers were designed using NCBI Primer Blast online software. Table S2. Alignment of statistical results of reads; Table S3. The 399 DEGs between H8C and L8C; Table S4. The 20 key GO terms of DEGs in H8C and L8C groups; Table S5. The 20 key KEGG pathways of DEGs in H8C and L8C groups; Table S6. The 1709 DEGs between HM and LM; Table S7. The 20 key GO terms of DEGs in HM and LM groups; Table S8. The 20 key KEGG pathways of DEGs in HM and LM groups; Table S9. The 66 DEGs between H8C and L8C groups and HM and LM groups. Table S10. The 66 DEGs from the KEGG enrichment analysis; Table S11. The 132 DEGs were screened to build the PPI network in HM and LM groups; Table S12. The 22 hub genes were identified.

## References

[1] Amoah E.A., Gelaye S. (1997). Biotechnological advances in goat reproduction. J. Anim. Sci..

[2] Kussano N.R., Leme L.O., Guimarães A.L., Franco M.M., Dode M.A. (2016). Molecular markers for oocyte competence in bovine cumulus cells. Theriogenology.

[3] Maj T., Wang W., Crespo J., Zhang H., Wang W., Wei S., Zhao L., Vatan L., Shao I., Szeliga W. (2017). Oxidative stress controls regulatory T cell apoptosis and suppressor activity and PD-L1-blockade resistance in tumor. Nat. Immunol..

[4] Kesavardhana S., Kanneganti T.D. (2018). Stressed-out ROS take a silent death route. Nat. Immunol..

[5] Sies H. (2015). Oxidative stress: A concept in redox biology and medicine. Redox Biol..

[6] Takahashi M. (2012). Oxidative stress and redox regulation on in vitro development of mammalian embryos. J. Reprod. Dev..

[7] Fabian D., Koppel J., Maddox-Hyttel P. (2005). Apoptotic processes during mammalian preimplantation development. Theriogenology.

[8] Jurisicova A., Antenos M., Varmuza S., Tilly J.L., Casper R.F. (2003). Expression of apoptosis-related genes during human preimplantation embryo development: Potential roles for the Harakiri gene product and Caspase-3 in blastomere fragmentation. Mol. Hum. Reprod..

[9] Jurisicova A., Latham K.E., Casper R.F., Casper R.F., Varmuza S.L. (1998). Expression and regulation of genes associated with cell death during murine preimplantation embryo development. Mol. Reprod. Dev..

[10] Byrne A.T., Southgate J., Brison D.R., Leese H.J. (1999). Analysis of apoptosis in the preimplantation bovine embryo using TUNEL. J. Reprod. Fertil..

[11] Rinaudo P.F., Giritharan G., Talbi S., Dobson A.T., Schultz R.M. (2006). Effects of oxygen tension on gene expression in preimplantation mouse embryos. Fertil. Steril..

[12] Alahmar A.T. (2019). Role of Oxidative Stress in Male Infertility: An Updated Review. J. Hum. Reprod. Sci..

[13] Thompson J.G., Partridge R.J., Houghton F.D., Cox C.I., Leese H.J. (1996). Oxygen uptake and carbohydrate metabolism by in vitro derived bovine embryos. J. Reprod. Fertil..

[14] Fischer B., Bavister B.D. (1993). Oxygen tension in the oviduct and uterus of rhesus monkeys, hamsters and rabbits. J. Reprod. Fertil..

[15] Kasterstein E., Strassburger D., Komarovsky D., Bern O., Komsky A., Raziel A., Friedler S., Ron-El R. (2013). The effect of two distinct levels of oxygen concentration on embryo development in a sibling oocyte study. J. Assist. Reprod. Genet..

[16] Yang H.W., Hwang K.J., Kwon H.C., Kim H.S., Choi K.W., Oh K.S. (1998). Detection of reactive oxygen species (ROS) and apoptosis in human fragmented embryos. Hum. Reprod..

[17] Van Soom A., Yuan Y.Q., Peelman L.J., de Matos D.G., Dewulf J., Laevens H., de Kruif A. (2002). Prevalence of apoptosis and inner cell allocation in bovine embryos cultured under different oxygen tensions with or without cysteine addition. Theriogenology.

[18] Karagenc L., Sertkaya Z., Ciray N., Ulug U., Bahceci M. (2004). Impact of oxygen concentration on embryonic development of mouse zygotes. Reprod. Biomed. Online.

[19] Kitagawa Y., Suzuki K., Yoneda A., Watanabe T. (2004). Effects of oxygen concentration and antioxidants on the in vitro developmental ability, production of reactive oxygen species (ROS), and DNA fragmentation in porcine embryos. Theriogenology.

[20] Lee S.C., Seo H.C., Lee J., Jun J.H., Choi K.W. (2019). Effects of dynamic oxygen concentrations on the development of mouse pre- and peri-implantation embryos using a double-channel gas supply incubator system. Clin. Exp. Reprod. Med..

[21] Deng M., Liu Z., Ren C., Zhang G., Pang J., Zhang Y., Wang F., Wan Y. (2018). Long noncoding RNAs exchange during zygotic genome activation in goat. Biol. Reprod..

[22] Picelli S., Faridani O.R., Björklund A.K., Winberg G., Sagasser S., Sandberg R. (2014). Full-length RNA-seq from single cells using Smart-seq2. Nat. Protoc..

[23] Deng M., Chen B., Liu Z., Cai Y., Wan Y., Zhang G., Fan Y., Zhang Y., Wang F. (2020). YTHDF2 Regulates Maternal Transcriptome Degradation and Embryo Development in Goat. Front. Cell Dev. Biol..

[24] Wan Y., Deng M., Zhang G., Ren C., Zhang H., Zhang Y., Wang L., Wang F. (2016). Abnormal expression of DNA methyltransferases and genomic imprinting in cloned goat fibroblasts. Cell. Biol. Int..

[25] Liu Z., Zhang G., Deng M., Yang H., Pang J., Cai Y., Wan Y., Wang F. (2020). Inhibition of lysine-specific histone demethylase 1A results in meiotic aberration during oocyte maturation in vitro in goats. Theriogenology.

[26] Schultz R.M., Stein P., Svoboda P. (2018). The oocyte-to-embryo transition in mouse: Past, present, and future. Biol. Reprod..

[27] Telford N.A., Watson A.J., Schultz G.A. (1990). Transition from maternal to embryonic control in early mammalian development: A comparison of several species. Mol. Reprod. Dev..

[28] Lee M.T., Bonneau A.R., Giraldez A.J. (2014). Zygotic genome activation during the maternal-to-zygotic transition. Annu. Rev. Cell Dev. Biol..

[29] Tominaga Y., Li C., Wang R.H., Deng C.X. (2006). Murine Wee1 plays a critical role in cell cycle regulation and pre-implantation stages of embryonic development. Int. J. Biol. Sci..

[30] Lee G.S., Kim H.S., Hwang W.S., Hyun S.H. (2008). Characterization of porcine growth differentiation factor-9 and its expression in oocyte maturation. Mol. Reprod. Dev..

[31] Bebbere D., Bogliolo L., Ariu F., Fois S., Leoni G.G., Tore S., Succu S., Berlinguer F., Naitana S., Ledda S. (2008). Expression pattern of zygote arrest 1 (ZAR1), maternal antigen that embryo requires (MATER), growth differentiation factor 9 (GDF9) and bone morphogenetic protein 15 (BMP15) genes in ovine oocytes and in vitro-produced preimplantation embryos. Reprod. Fertil. Dev..

[32] Yu C., Ji S.Y., Sha Q.Q., Dang Y., Zhou J.J., Zhang Y.L., Liu Y., Wang Z.W., Hu B., Sun Q.Y. (2016). BTG4 is a meiotic cell cycle-coupled maternal-zygotic-transition licensing factor in oocytes. Nat. Struct. Mol. Biol..

[33] Wang J., Zhang Y., Hou J., Qian X., Zhang H., Zhang Z., Li M., Wang R., Liao K., Wang Y. (2016). Ube2s regulates Sox2 stability and mouse ES cell maintenance. Cell Death Differ..

[34] Burns K.H., Viveiros M.M., Ren Y., Wang P., DeMayo F.J., Frail D.E., Eppig J.J., Matzuk M.M. (2003). Roles of NPM2 in chromatin and nucleolar organization in oocytes and embryos. Science.

[35] Habibi R., Hosseini S.M., Zadegan F.G., Hajian M., Ostadhosseini S., Vash N.T., Naddafpour A., Nasr Esfahani M.H. (2018). Functional characterization of NANOG in goat pre-implantation embryonic development. Theriogenology.

[36] Favetta L.A., St John E.J., King W.A., Betts D.H. (2007). High levels of p66shc and intracellular ROS in permanently arrested early embryos. Free. Radic. Biol. Med..

[37] Gaspar R.C., Arnold D.R., Corrêa C.A., da Rocha C.V., Penteado J.C., Del Collado M., Vantini R., Garcia J.M., Lopes F.L. (2015). Oxygen tension affects histone remodeling of in vitro-produced embryos in a bovine model. Theriogenology.

[38] Agarwal A., Saleh R.A., Bedaiwy M.A. (2003). Role of reactive oxygen species in the pathophysiology of human reproduction. Fertil. Steril..

[39] Adam A.A., Takahashi Y., Katagiri S., Nagano M. (2004). Effects of oxygen tension in the gas atmosphere during in vitro maturation, in vitro fertilization and in vitro culture on the efficiency of in vitro production of mouse embryos. Jpn. J. Vet. Res..

[40] Yuan Y.Q., Van Soom A., Coopman F.O., Mintiens K., Boerjan M.L., Van Zeveren A., de Kruif A., Peelman L.J. (2003). Influence of oxygen tension on apoptosis and hatching in bovine embryos cultured in vitro. Theriogenology.

[41] Mishra A., Reddy I.J., Gupta P.S., Mondal S. (2016). L-carnitine Mediated Reduction in Oxidative Stress and Alteration in Transcript Level of Antioxidant Enzymes in Sheep Embryos Produced In Vitro. Reprod. Domest. Anim..

[42] Nilson K.A., Lawson C.K., Mullen N.J., Ball C.B., Spector B.M., Meier J.L., Price D.H. (2017). Oxidative stress rapidly stabilizes promoter-proximal paused Pol II across the human genome. Nucleic Acids Res..

[43] Lettieri-Barbato D., Ioannilli L., Aquilano K., Ciccarone F., Rosina M., Ciriolo M.R. (2019). FoxO1 localizes to mitochondria of adipose tissue and is affected by nutrient stress. Metabolism.

[44] Xu X., He L., Zhang A., Li Q., Hu W., Chen H., Du J., Shen J. (2015). Toxoplasma gondii isolate with genotype Chinese 1 triggers trophoblast apoptosis through oxidative stress and mitochondrial dysfunction in mice. Exp. Parasitol..

[45] Hu D.B., Li Z.S., Ali I., Xu L.J., Fang N.Z. (2017). Effect of potential role of p53 on embryo development arrest induced by H(2)O(2) in mouse. In Vitro Cell. Dev. Biol. Anim..

[46] Wang H., Ye Y., Yu Z.L. (2014). Proteomic and functional analyses demonstrate the involvement of oxidative stress in the anticancer activities of oridonin in HepG2 cells. Oncol. Rep..

[47] Shen Y.H., Song G.X., Liu Y.Q., Sun W., Zhou L.J., Liu H.L., Yang R., Sheng Y.H., Qian L.M., Kong X.Q. (2012). Silencing of FABP3 promotes apoptosis and induces mitochondrion impairment in embryonic carcinoma cells. J. Bioenerg. Biomembr..

[48] Bonnet F., Molina A., Roussat M., Azais M., Bel-Vialar S., Gautrais J., Pituello F., Agius E. (2018). Neurogenic decisions require a cell cycle independent function of the CDC25B phosphatase. Elife.

[49] Cao J., Dong J., Wang Y., Chen Y. (2019). The expressions of DNA methyltransferase 1 (DNMT1) and cyclin A1 (CCNA1) in cervical carcinogenesis. Int. J. Clin. Exp. Pathol..

[50] Moiseeva T.N., Qian C., Sugitani N., Osmanbeyoglu H.U., Bakkenist C.J. (2019). WEE1 kinase inhibitor AZD1775 induces CDK1 kinase-dependent origin firing in unperturbed G1- and S-phase cells. Proc. Natl. Acad. Sci. USA.

[51] Martinsson-Ahlzén H.S., Liberal V., Grünenfelder B., Chaves S.R., Spruck C.H., Reed S.I. (2008). Cyclin-dependent kinase-associated proteins Cks1 and Cks2 are essential during early embryogenesis and for cell cycle progression in somatic cells. Mol. Cell. Biol..

[52] Ma S., Charron J., Erikson R.L. (2003). Role of Plk2 (Snk) in mouse development and cell proliferation. Mol. Cell. Biol..

[53] Huang L., Min J.N., Masters S., Mivechi N.F., Moskophidis D. (2007). Insights into function and regulation of small heat shock protein 25 (HSPB1) in a mouse model with targeted gene disruption. Genesis.

[54] Ruzov A., Dunican D.S., Prokhortchouk A., Pennings S., Stancheva I., Prokhortchouk E., Meehan R.R. (2004). Kaiso is a genome-wide repressor of transcription that is essential for amphibian development. Development.

[55] Li H.B., Jin X.Q., Jin X., Guo Z.H., Ding X.H., Wang Q., Liu R.Z. (2018). BMP4 knockdown of NCSCs leads to aganglionosis in the middle embryonic stage. Mol. Med. Rep..

[56] Tallquist M.D., Weismann K.E., Hellström M., Soriano P. (2000). Early myotome specification regulates PDGFA expression and axial skeleton development. Development.

[57] Park H., Bourla A.B., Kastner D.L., Colbert R.A., Siegel R.M. (2012). Lighting the fires within: The cell biology of autoinflammatory diseases. Nat. Rev. Immunol..

[58] Du X., Li Q., Cao Q., Wang S., Liu H., Li Q. (2019). Integrated Analysis of miRNA-mRNA Interaction Network in Porcine Granulosa Cells Undergoing Oxidative Stress. Oxid. Med. Cell. Longev..

[59] Ebrahimi K.B., Cano M., Rhee J., Datta S., Wang L., Handa J.T. (2018). Oxidative Stress Induces an Interactive Decline in Wnt and Nrf2 Signaling in Degenerating Retinal Pigment Epithelium. Antioxid. Redox Signal..

[60] Shen M., Jiang Y., Guan Z., Cao Y., Li L., Liu H., Sun S.C. (2017). Protective mechanism of FSH against oxidative damage in mouse ovarian granulosa cells by repressing autophagy. Autophagy.

[61] Wang Y., Li J., Gao Y., Luo Y., Luo H., Wang L., Yi Y., Yuan Z., Jim Xiao Z.X. (2019). Hippo kinases regulate cell junctions to inhibit tumor metastasis in response to oxidative stress. Redox Biol..

[62] Gupta A., Anjomani-Virmouni S., Koundouros N., Dimitriadi M., Choo-Wing R., Valle A., Zheng Y., Chiu Y.H., Agnihotri S., Zadeh G. (2017). PARK2 Depletion Connects Energy and Oxidative Stress to PI3K/Akt Activation via PTEN S-Nitrosylation. Mol. Cell.

[63] Kang K.A., Wang Z.H., Zhang R., Piao M.J., Kim K.C., Kang S.S., Kim Y.W., Lee J., Park D., Hyun J.W. (2010). Myricetin protects cells against oxidative stress-induced apoptosis via regulation of PI3K/Akt and MAPK signaling pathways. Int. J. Mol. Sci..

