# Supplementary material for: Comprehensive Transcriptome Analysis of mRNA Expression Patterns of Early Embryo Development in Goat under Hypoxic and Normoxic Conditions
Source: Biology (Basel). 2021 Apr 28;10(5):381. doi: 10.3390/biology10050381 (PMC8146044; doi:10.3390/biology10050381)
Supplement: Supplementary file 1 [file biology-10-00381-s001.zip › biology-1188034-supplementary.pdf]

Comprehensive Analysis of mRNA Expression Patterns in Different Oxygen Concentrations of Early Embryo Development

Yongjie Wan,[1] Dongxu Li,[1] Mingtian Deng,[1] Zifei Liu,[1] Liang Liu,[1] Feng Wang,[1]

[1] Jiangsu Livestock Embryo Engineering Laboratory, College of Animal Science and Technology, Nanjing Agricultural University, Nanjing. 210095, China

Correspondence should be addressed to Yongjie Wan; wanyongjie@njau.edu.cn

Supplementary Materials

Figure S1

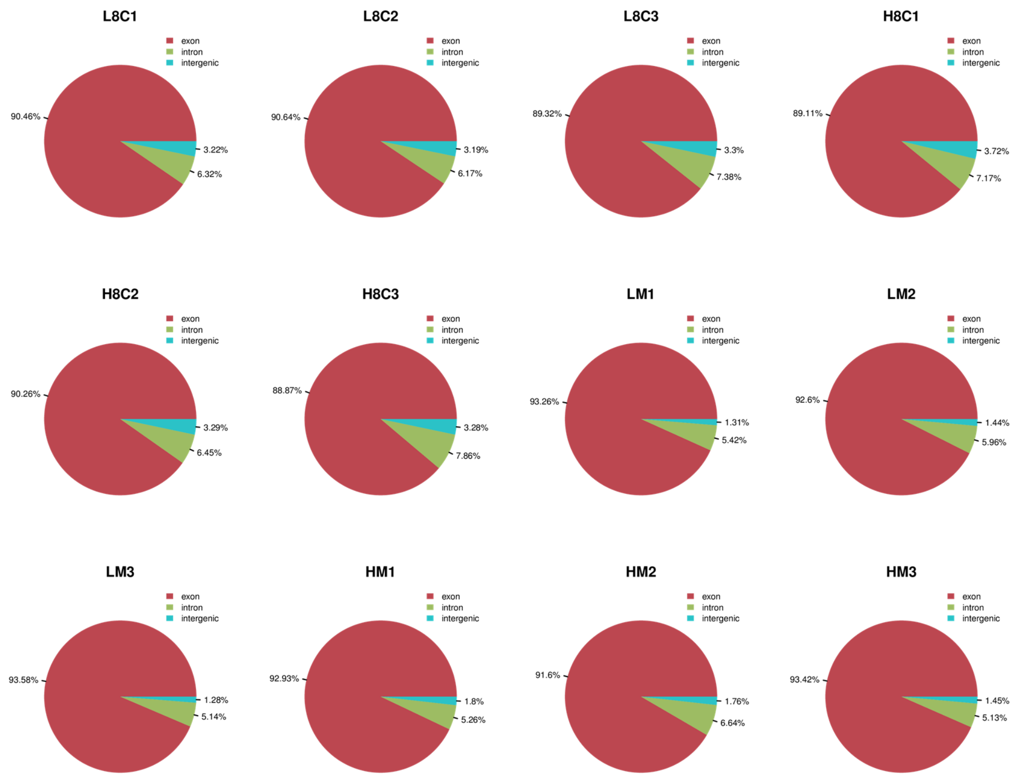

Figure S1. The reference genome alignment region distribution, L8C and H8C were 8-cell stage embryos under hypoxia and normoxia conditions. LM and HM were blastocyst stage embryos under hypoxia and normoxia conditions.

Figure S2

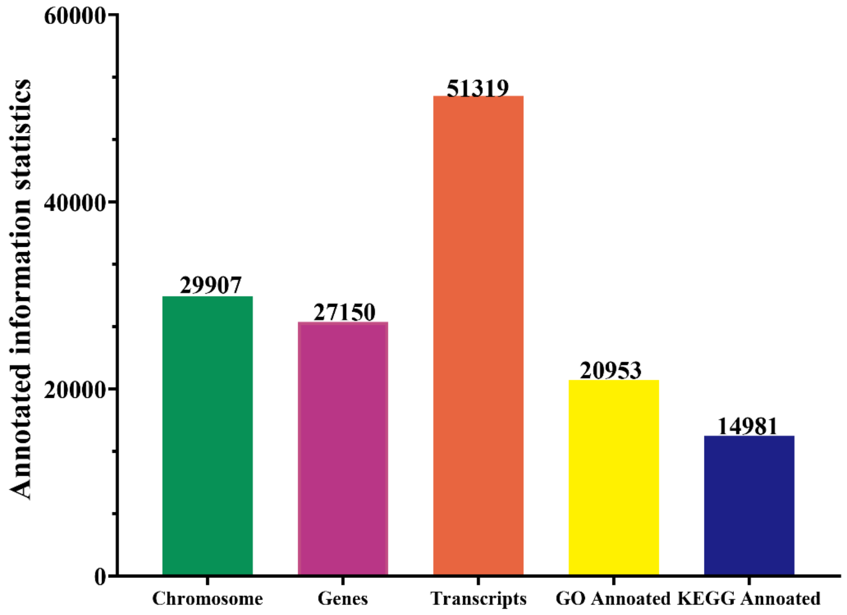

Figure S2. The known annotation information in the species database was counted.

Figure S3

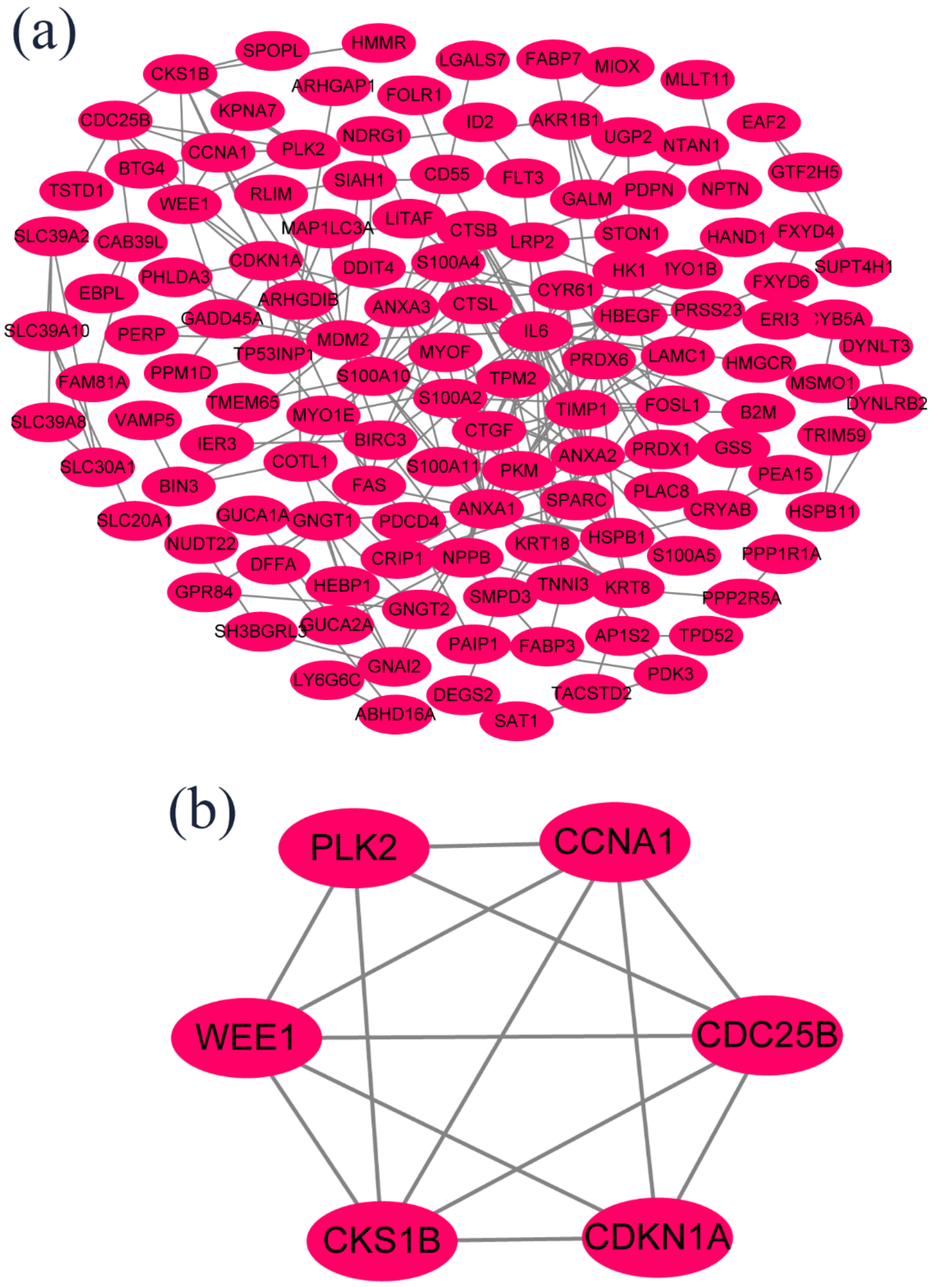

Figure S3. (a) The PPI network established using 132 up-regulated DEGs (FPKM > 2 and fold change > 2). (b) The most important module of the PPI network including 6 nodes and 14 edges.

Figure S4

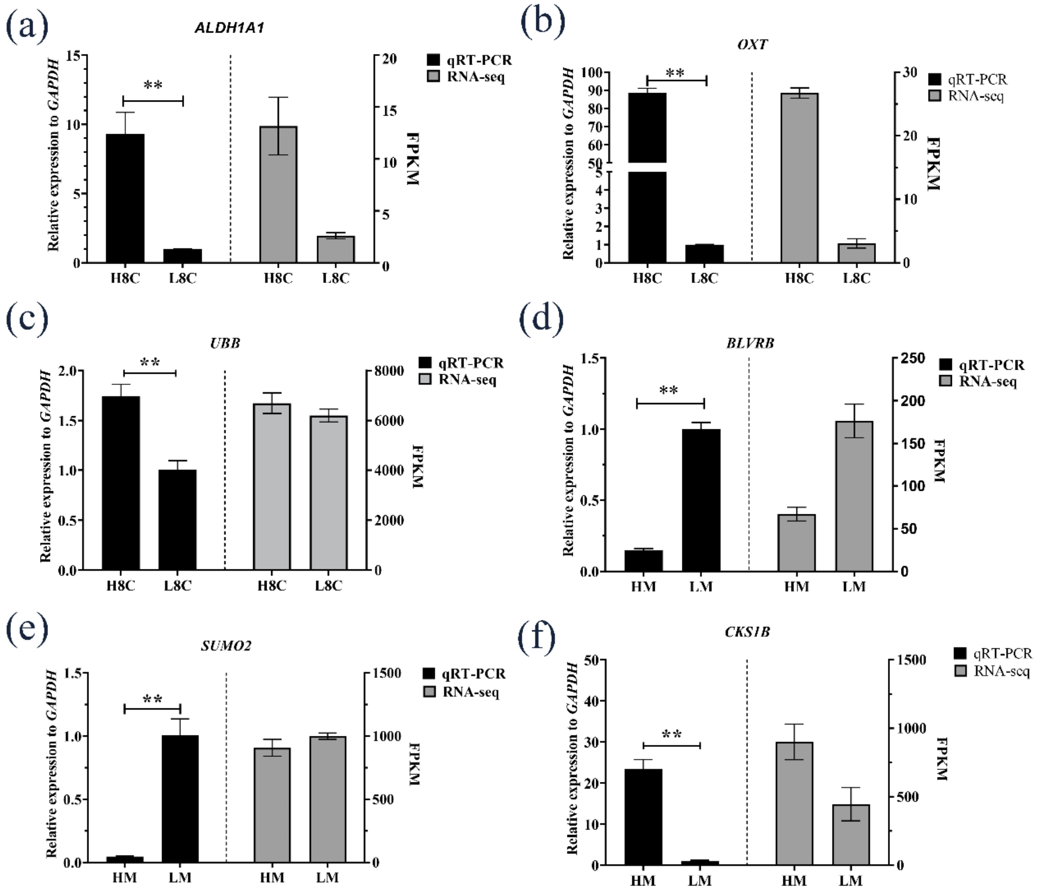

Figure S4. Validation of sequencing data. The quantitative real-time PCR (qRT-PCR) results showed the same trend compared with the sequencing data. The relative mRNA expression levels of target genes were calculated using the comparative $2^{-\Delta\Delta Ct}$ method after normalization to GAPDH control.

Supplementary Table S1: The PCR primers were designed using NCBI Primer Blast online software.

Gene name	Primer pairs sequence	Product size (bp)	Annealing temperature (℃)
OXT	F: GCTGCCAAGAGGAGAACTACC<br>R: CCTGGGGATGATTACAGAGGG	220	60
ALDH1A1	F: TCCCGCAACTGAGGAGAAAC<br>R: TCATAGCCTCCATTGTCGCC	203	60
CKS1B	F: TGAATCCAGCAGAACACGCT<br>R: ACATACACACAACACCTGGCA	122	60
UBB	F: CGGAGCACTTAGACGCGA<br>R: ACCTGTGAGTGAATGCGAAGA	100	60
SUMO2	F: GATGGTTCTGTGGTGCAGTTT<br>R: CATCTTCCATCTCCAACTGTGC	166	60
BLVRB	F: GACGCTGTCATCGTACTGCT<br>R: GGACATGGTCATCGGTCACA	196	60

Supplementary Table S2: Alignment of statistical results of reads.

Sample	Valid reads	Mapped reads	Unique reads	Multi reads	Non-splice reads	Splice reads
L8C1	48,510,992	43,157,263 (88.96%)	32,159,814 (66.29%)	10,997,449 (22.67%)	20,008,133 (41.24%)	19,948,596 (41.12%)
L8C2	38,394,118	34,010,274 (88.58%)	25,334,650 (65.99%)	86,756,24 (22.60%)	15,717,405 (40.94%)	15,701,950 (40.90%)
L8C3	43,705,096	39,035,674 (89.32%)	28,789,679 (65.87%)	10,245,995 (23.44%)	18,653,972 (42.68%)	16,648,146 (38.09%)
H8C1	43,728,392	38,809,411 (88.75%)	28,794,776 (65.85%)	10,014,635 (22.90%)	20,290,769 (46.40%)	15,538,212 (35.53%)
H8C2	44,487,354	39,855,685 (89.59%)	29,642,543 (66.63%)	10,213,142 (22.96%)	19,223,173 (43.21%)	17,523,969 (39.39%)
H8C3	46,129,430	40,589,565 (87.99%)	29,116,272 (63.12%)	11,473,293 (24.87%)	20,163,265 (43.71%)	15,964,814 (34.61%)
LM1	33,675,350	28,389,192 (84.30%)	19,652,872 (58.36%)	8,736,320 (25.94%)	11,078,972 (32.90%)	13,175,840 (39.13%)
LM2	42,594,282	35,888,810 (84.26%)	25,002,153 (58.70%)	10,886,657 (25.56%)	14,429,656 (33.88%)	16,349,625 (38.38%)
LM3	32,758,780	27,813,051 (84.90%)	19,661,647 (60.02%)	8,151,404 (24.88%)	11,090,144 (33.85%)	12,879,814 (39.32%)
HM1	45,522,090	38,641,158 (84.88%)	26,775,817 (58.82%)	11,865,341 (26.07%)	16,723,440 (36.74%)	16,362,033 (35.94%)
HM2	38,119,496	32,979,123 (86.52%)	23,258,980 (61.02%)	9,720,143 (25.50%)	14,221,322 (37.31%)	14,343,367 (37.63%)
HM3	44,799,218	39,157,775 (87.41%)	27,358,087 (61.07%)	11,799,688 (26.34%)	15,573,580 (34.76%)	17,942,133 (40.05%)

Supplementary Table S3: The 399 DEGs between H8C and L8C.

gene_name	log2(fold change)	pval	regulation
LOC108637665	6.470376438	9.66449E-15	up
VNN1	15.33243986	1.77872E-12	up
ADGRE1	15.40138563	8.54385E-12	up
LOC102174969	12.32607262	1.44663E-11	up
RETN	17.14840106	9.61053E-10	up
LOC102169149	9.288866659	1.43191E-09	up
MMP9	17.65947476	1.71318E-08	up
S100A9	9.605259701	1.64372E-07	up
S100A8	8.812529981	1.67012E-07	up
LOC102181154	11.42660844	1.70298E-07	up
LOC102181552	7.912593951	3.91265E-07	up
LOC102170772	7.150782323	1.55561E-06	up
PPBP	7.674903938	2.74559E-06	up
P2RY13	9.529972083	3.00935E-06	up
RGS1	5.099128792	3.62949E-06	up
LOC108635183	14.71466588	3.83821E-06	up
OXT	5.255074346	4.07364E-06	up
CLIC2	7.691440198	7.29062E-06	up
LOC102181582	7.041543145	1.55383E-05	up
LOC102168428	4.99751923	2.3568E-05	up
LOC102170310	4.9076411	2.53015E-05	up
LOC102182207	3.429166503	2.76316E-05	up
CLEC9A	12.11177252	3.50147E-05	up
LOC102186356	5.80218601	3.70364E-05	up
KCND2	10.90974561	4.54645E-05	up
IGSF6	4.943599682	4.81222E-05	up
CLEC4D	7.698225538	5.36945E-05	up
LOC102172037	5.837557287	6.81074E-05	up
LOC102175889	5.795802368	6.87958E-05	up
MSMB	3.626538309	7.2574E-05	up
LOC102181854	5.075540972	7.32716E-05	up
LOC108634452	3.375309355	9.39953E-05	up
LOC102183943	5.26086436	0.000227869	up
TREM1	13.84129341	0.000310298	up
LOC102173569	7.630137382	0.000407842	up
FGL2	4.238183905	0.000423835	up
LOC102177727	4.508398862	0.000434254	up
TMEM176A	5.319036799	0.000455504	up
TBXAS1	4.168536301	0.000535848	up
ORMDL1	1.865995946	0.00058171	up
PLBD1	4.210497821	0.000586398	up

LOC102182340	6.099577565	0.000591902	up
RNASE6	4.42525079	0.000608005	up
LIPA	3.112057489	0.000617311	up
LOC102182485	4.023361734	0.000652891	up
CLEC3B	3.625840424	0.000717602	up
LOC108635375	5.164635869	0.00086042	up
CTSL	3.393422525	0.000916781	up
C3H1orf162	5.215297398	0.000926074	up
LOC102173131	2.961293334	0.000980115	up
PECAM1	3.900909817	0.001071658	up
ABCA6	4.333177155	0.001253971	up
FTH1	2.678867506	0.00132346	up
CD14	4.258720845	0.001369577	up
ADAM28	3.462086395	0.001375284	up
IL1B	4.412501789	0.001458484	up
LOC108634577	4.748624846	0.001497809	up
LOC102187787	2.258675336	0.00163933	up
SELL	3.691828615	0.001676659	up
LOC102171003	5.183890305	0.001788322	up
SAMSN1	4.281427969	0.001926247	up
FOLR2	4.63893639	0.001944413	up
LOC102168808	2.49726636	0.002058077	up
DTX3L	4.729920634	0.002067758	up
F2RL2	4.102744696	0.002070395	up
P2RY12	6.92031986	0.002121719	up
LCP1	2.568148686	0.002190768	up
MSR1	3.816189279	0.002231391	up
LOC108634441	1.701499292	0.002262461	up
TFEC	4.77041143	0.002271013	up
LY96	3.795138472	0.00233727	up
ALOX5AP	2.034622568	0.00242072	up
LOC102184859	4.833846558	0.002505224	up
LOC102174762	3.765231218	0.002516769	up
FCGRT	2.787465015	0.002537435	up
LOC108638098	2.541002467	0.002559683	up
PI15	2.955037343	0.002622933	up
LOC102180110	2.567067468	0.002958561	up
LOC108634439	1.666142728	0.003065593	up
SAMD9	3.464762896	0.003072639	up
LOC102173987	4.3460243	0.003093165	up
DDAH1	6.816452291	0.003140257	up
ITGA1	4.540706897	0.00316756	up
PTH	2.987298746	0.003287355	up
LOC108634456	2.554152921	0.003290155	up

CLEC4E	4.469289511	0.003459139	up
FBP1	4.047194433	0.003551827	up
LOC102174023	4.475317527	0.003731477	up
LOC102186527	3.560735757	0.003850491	up
TNFSF8	5.206019591	0.003918282	up
HNMT	3.399532696	0.004102179	up
LOC106503022	1.910790037	0.004112797	up
LOC106503930	2.628598772	0.004285621	up
LST1	4.205754577	0.004292748	up
CLEC6A	3.46130136	0.004417382	up
CXCL8	5.226296994	0.004585142	up
ITGAM	5.235256727	0.005116518	up
TMEM200A	3.044857781	0.005173212	up
LOC102170764	1.968446608	0.005196622	up
CREG1	2.907619049	0.005453721	up
RIDA	1.427598623	0.005462372	up
VSIG4	6.365380449	0.005701212	up
SGPP1	4.077897204	0.005837644	up
LOC106502184	2.462140343	0.005857964	up
LPAR6	3.231417335	0.00601026	up
CLEC5A	4.010804031	0.006054757	up
LOC102169654	2.302264342	0.00637447	up
FYB	3.342010978	0.006428491	up
LOC108634243	2.5157372	0.006453625	up
LOC102168547	2.418848243	0.006899789	up
LOC102169183	4.099820588	0.006937593	up
TREM2	2.611535874	0.007061055	up
ACOX1	2.168290569	0.007191789	up
CD48	3.177792569	0.007253124	up
DOCK10	2.97149128	0.007272028	up
LOC106501751	3.313458318	0.007390125	up
ATP6AP2	1.564398003	0.007401165	up
PLEK	2.632643774	0.007431985	up
CD36	1.905235285	0.007599855	up
LOC102181202	3.181070811	0.007657061	up
EDA	3.535479449	0.007685706	up
GMFG	3.525029524	0.008050546	up
LOC102177400	2.027038743	0.008267725	up
PLA2G7	1.796484778	0.008419012	up
LIMS1	3.124691526	0.008806006	up
TNFAIP6	1.651145553	0.008809263	up
MX2	4.046921048	0.009091812	up
C16H1orf21	3.38542267	0.009329322	up
HEXB	2.108859634	0.00943605	up

MILR1	4.919637558	0.009576821	up
TLR7	2.454649061	0.00959465	up
SRGN	2.351078806	0.009627525	up
SLC13A5	3.070207636	0.009686888	up
HEBP1	3.088254862	0.009793689	up
CSF2RA	3.602040328	0.009813657	up
LOC102186003	1.453051961	0.009863343	up
B2M	2.069211725	0.009879634	up
LOC102169968	1.859086725	0.009963016	up
TLR4	3.937369266	0.010012426	up
LOC102188267	3.031889693	0.010094307	up
PFKP	4.065636282	0.010203972	up
CTSK	4.513627575	0.010702499	up
LOC108637984	3.056465469	0.010740109	up
LOC102182395	3.417143281	0.010760808	up
VAMP5	3.218802165	0.011040001	up
ENPP5	2.087749617	0.011137182	up
PTN	2.59601461	0.011266756	up
CRABP2	1.796280941	0.011718534	up
WIPF1	3.659362549	0.011964218	up
LOC102175526	2.839189089	0.011977788	up
FAM174B	3.478073199	0.012242623	up
ADGRF5	2.307249173	0.012457351	up
GSR	2.009376704	0.012560619	up
LOC100861168	3.649945534	0.012589408	up
LOC106502029	2.52340717	0.012600985	up
PKIB	2.845752451	0.012674819	up
CTGF	2.374831231	0.01268136	up
LOC102184237	2.117141258	0.012936436	up
GJA1	1.719788501	0.013059822	up
LOC102186814	3.264313906	0.013092538	up
RNF213	2.398866709	0.013379486	up
EVI2B	3.685207892	0.013618266	up
SCIMP	3.033375667	0.014133332	up
LOC108635630	3.803737223	0.014276976	up
ACTG2	2.839106082	0.014334073	up
WDR5B	3.395345087	0.014382708	up
OLR1	2.261185747	0.014619056	up
LOC102188826	1.796813786	0.014673631	up
IL2RG	3.665103164	0.014773805	up
ZNF711	1.862903971	0.014784672	up
LOC102182181	1.30136703	0.014882527	up
LOC102185698	3.957629666	0.014958274	up
RNF125	2.208654062	0.014969007	up

CXCL10	3.850949306	0.014977228	up
NUPR1	3.179706755	0.014984193	up
SNX10	1.769957999	0.015357172	up
PLD4	5.195668883	0.015599872	up
LOC102179924	1.374656137	0.016070991	up
ALDH1A1	1.488573121	0.016285205	up
NCF2	4.049836648	0.016431095	up
IFI44L	3.003168116	0.016458393	up
LRRK2	2.36016125	0.016470518	up
LOC102168687	2.433649798	0.017376079	up
ISG15	2.756139964	0.017487778	up
KCNMB3	2.440108676	0.017522332	up
UBE2J1	2.567288189	0.018043491	up
C5AR1	2.668109385	0.018111246	up
LOC102181993	3.858919099	0.018198803	up
ADIPOQ	5.11333001	0.018308244	up
LOC102180888	3.052771991	0.019245457	up
CD86	1.848566467	0.019290006	up
BCL2A1	2.36028658	0.019442243	up
CMPK2	2.059690808	0.019486281	up
ITGA4	3.50288396	0.019800995	up
GPR18	3.017777245	0.019949963	up
LOC108634443	1.287104585	0.02008593	up
PCDHB14	3.316399093	0.020209465	up
AIF1	2.935979579	0.020233009	up
LOC108634440	1.294837507	0.021021176	up
DPYD	2.532208605	0.021195491	up
LOC108636385	2.604191839	0.021224323	up
MRC1	1.354455933	0.021631626	up
LRG1	4.872418929	0.021808783	up
SCP2	1.558951367	0.021819241	up
HMGN5	1.235676693	0.021893916	up
LOC108638299	2.889256877	0.022098674	up
PRKAR2B	1.255190881	0.022488729	up
LOC102177561	3.221580642	0.022567468	up
ANKRD66	2.18960772	0.022877408	up
ERAP2	2.831179757	0.023397052	up
LOC102169861	1.377666691	0.023523871	up
SP3	1.249512826	0.023909503	up
TPD52	1.338447974	0.024378933	up
KIF21A	2.289498766	0.024718271	up
AMMECR1	2.039227926	0.024801613	up
LAT2	4.160929776	0.024829568	up
CXCR4	1.667876304	0.02489304	up

SLAMF6	1.927965488	0.024984544	up
CLN5	2.142498744	0.025027845	up
LOC108634238	2.358482149	0.025656528	up
INAFM2	2.74384697	0.025714763	up
GALNT1	1.175582283	0.026299006	up
LOC102180655	2.339445809	0.026366253	up
LOC100860930	1.244051815	0.026455323	up
ANKH	1.76770015	0.026527664	up
HCLS1	3.144491251	0.026585246	up
BEX5	2.355720004	0.0265996	up
LOC108633237	1.742636	0.026632868	up
SPI1	3.976167834	0.027072045	up
TAB3	1.471263756	0.027125374	up
LY86	2.89346664	0.027205616	up
MAFB	2.804311557	0.027781822	up
ADORA3	3.344524136	0.02797551	up
NKG7	3.321357907	0.027996895	up
LOC100860813	3.317056007	0.028038553	up
LOC102190537	2.812939026	0.028393402	up
LOC102171127	1.626261429	0.028490233	up
NNAT	3.124314812	0.028583376	up
CD68	2.631071605	0.028973793	up
TMBIM4	1.21335886	0.030181328	up
KCNA3	1.517020769	0.030229215	up
STMN2	2.571393108	0.030540863	up
LOC102185515	2.927914841	0.030642837	up
BCL2L15	1.380420397	0.03088832	up
LAPTM5	1.864986935	0.031094703	up
LOC102180664	2.069852747	0.031439589	up
LOC108637868	1.128910663	0.031508126	up
RAN	1.131124462	0.031557295	up
SPATS2L	2.84429141	0.031715651	up
PLAUR	4.224985203	0.032483682	up
LOC106503821	3.973334526	0.032768302	up
TNFSF13B	1.962529264	0.032863849	up
MFGE8	1.993679025	0.032949262	up
AWAT2	3.233028512	0.03316807	up
LOC108634581	1.536828666	0.033346635	up
SMIM13	1.357162251	0.033414716	up
LOC102187211	1.585156825	0.03353426	up
ENPP1	1.191166736	0.033599775	up
BLOC1S2	1.766759199	0.033627979	up
LOC102189064	3.710835221	0.03374589	up
ARL4C	2.193169453	0.034113832	up

CTSA	1.99363755	0.034307272	up
IL15	1.876751268	0.034567076	up
SKIL	1.833522024	0.034685329	up
NT5C3A	1.993437719	0.03473356	up
IFIT2	2.611920108	0.035136645	up
APBB1IP	1.550850753	0.035180589	up
LOC102177263	3.850802652	0.035198663	up
CCNG1	1.001849716	0.035209262	up
MARCKS	1.779499843	0.035269675	up
C5AR2	2.988821581	0.035314962	up
SH3BGRL3	1.430824635	0.035353574	up
LOC102175755	2.3460393	0.035358436	up
APOOL	2.316782502	0.035367486	up
CHI3L1	3.859353431	0.03556377	up
GPX8	1.44627782	0.035741913	up
CXCL16	4.849109643	0.035790539	up
ASAH1	1.046330675	0.035912712	up
CTSS	3.448557632	0.03603837	up
MVD	2.592736113	0.036840044	up
LOC108633505	2.827320391	0.036898567	up
ACYP2	1.73646606	0.037124696	up
SLC11A1	4.062242785	0.037219385	up
EMB	2.128216082	0.037220218	up
PRKRA	1.359431461	0.037336511	up
SAT1	1.178381159	0.037526007	up
RAB31	2.570702907	0.037550827	up
BHLHE40	2.050309616	0.037617094	up
AIDA	1.066896119	0.037787012	up
KMO	3.922169191	0.037882037	up
ASRGL1	1.482887072	0.038056592	up
LOC108634582	1.473217053	0.038070227	up
TMEM213	2.076789525	0.03808464	up
GRK3	1.399670289	0.03814613	up
IFI44	1.932485356	0.038221054	up
EIF2AK2	1.812086126	0.038251467	up
PTPRC	1.464082928	0.038650785	up
LITAF	1.816578446	0.039514255	up
MSRB1	1.904234895	0.03960286	up
LOC108634547	1.573391493	0.04018756	up
SEMA4A	4.600464473	0.040394989	up
ITM2B	2.079601553	0.040668229	up
C5H12orf75	1.324956027	0.040721705	up
LOC100861338	3.286102362	0.04091816	up
SLC25A46	1.514159429	0.041065471	up

LOC108635601	1.539417357	0.041071587	up
LOC102186286	1.462035976	0.041157174	up
EGR2	1.992256774	0.041203784	up
DECR1	2.357282936	0.041564498	up
C1QC	4.021195516	0.041704102	up
LOC102180291	3.361508275	0.042102902	up
BEX3	1.621111758	0.042169838	up
ZSCAN2	3.061425126	0.042177725	up
LOC102180879	2.188034244	0.042495749	up
LOC108635382	1.455567085	0.042589992	up
ZNF189	2.072509951	0.042709949	up
MS4A8	3.056168612	0.04271287	up
NAP1L2	4.056588516	0.042883125	up
ZNF570	1.76419571	0.043151697	up
CXCL13	2.39117655	0.043168068	up
TRIM38	1.554531132	0.043287613	up
PIK3AP1	3.025580129	0.043361356	up
RBM11	1.963739915	0.043364441	up
COLEC11	2.853712368	0.043525791	up
MBNL1	1.417532113	0.043891956	up
C1QL3	2.361419656	0.043916971	up
CD55	1.161423783	0.044109255	up
TLDC2	1.841211485	0.044586735	up
PSMA2	1.699488576	0.044609332	up
CD1E	4.234955174	0.044611549	up
LOC108636735	1.163755906	0.044742974	up
LOC102184252	4.076594814	0.044804535	up
LOC102178379	2.936691393	0.045456874	up
MYL6	1.208443117	0.045627988	up
OSTM1	1.131197279	0.046234156	up
LOC108637516	1.031363245	0.046416865	up
TCEAL9	2.3711432	0.046498122	up
LOC102177044	2.309628473	0.046579418	up
LOC108634366	1.355989727	0.046988326	up
ITFG1	1.849204673	0.047093761	up
BLOC1S1	1.449793806	0.048635208	up
FBXO11	1.345448138	0.04868687	up
DOCK8	1.480376769	0.048753825	up
LOC108637161	1.073602108	0.048773719	up
SEMA4D	2.300304004	0.048878908	up
NEK6	2.617430336	0.048949875	up
COMMD1	1.56489253	0.049007044	up
LOC108634546	1.466664769	0.049150816	up
ZNF653	-11.92704458	0.000283575	down

LOC108635065	-13.16871442	0.000715131	down
LOC108633777	-6.383480164	0.001202531	down
PRMT8	-5.720462903	0.002599241	down
NDUFA4L2	-5.43550649	0.002958466	down
CACNA1C	-5.519928966	0.004364562	down
FZR1	-2.517780829	0.006628504	down
INSM2	-4.593538957	0.009187793	down
CRB3	-4.392670024	0.013296495	down
BRD3	-2.270362909	0.013407121	down
ZBTB49	-2.703054879	0.015315256	down
LDB1	-2.095986627	0.016209091	down
FOXR1	-1.973152548	0.016779329	down
USP19	-2.318244576	0.017314765	down
VPS18	-3.487491091	0.017382553	down
NOC4L	-2.459832364	0.018157886	down
LIG1	-1.947361615	0.020143722	down
USB1	-2.203869814	0.020377194	down
CASR	-2.428034181	0.022256573	down
SERTAD4	-3.235068199	0.022357809	down
C19H17orf96	-3.120912644	0.02243882	down
RRP1B	-1.938524548	0.023947211	down
PCBP4	-2.089787904	0.02468467	down
VAV2	-2.524508888	0.025821138	down
PRR19	-2.621062893	0.027235803	down
HUNK	-1.947945898	0.027277511	down
PIP5K1C	-2.027763222	0.028005742	down
GUCY1A2	-3.912537159	0.028745684	down
STK11IP	-2.288049562	0.030011261	down
CKMT1A	-1.681754149	0.030424718	down
C7H19orf44	-2.651281385	0.031161369	down
FBXW5	-3.56493044	0.03253447	down
PSRC1	-2.016379845	0.0333725	down
CLEC3A	-2.205025639	0.03346312	down
LOC106502244	-3.716794659	0.033986394	down
U2AF2	-1.871484698	0.034581015	down
PCIF1	-1.923095139	0.03491263	down
CARM1	-2.121075582	0.035044017	down
DDX42	-1.834294349	0.037020299	down
LOC102170412	-1.991986462	0.038594188	down
CUX2	-1.973945622	0.039085711	down
ADCK1	-2.276891415	0.041545353	down
ERCC2	-2.04938934	0.042537676	down
CD3EAP	-2.258824994	0.042661602	down
CDK2	-1.713370035	0.043336327	down

RAVER1	-2.427546741	0.046913531	down
SEC16A	-1.885354843	0.047205452	down
BAIAP2	-1.886126529	0.047931617	down
DMRTC2	-1.84806027	0.047937204	down
KANSL3	-1.80178717	0.048220722	down
PRR14	-1.718934617	0.049639611	down

$|\ Log_2(\text{Fold change})\ | \geq 1$ and adjusted FDR < 0.05 was the cut-off criteria for DEGs.

Supplementary Table S4: The 1,709 DEGs between HM and LM

gene_name	log2(fold change)	pval	regulation
LOC108637993	4.621937299	1.29E-29	up
LOC102185054	5.732597977	2.35E-22	up
CCL17	7.666414256	1.11E-20	up
LOC108636734	7.82699719	2.9E-20	up
SLC23A1	4.273324356	1.11E-19	up
CDKN1A	3.361729806	1.95E-19	up
ZNF385A	13.59125059	3.87E-17	up
DSC3	5.033848176	5.86E-17	up
LOC102169211	16.91087664	2.06E-16	up
GAPDH	3.505566547	2.09E-16	up
DGKA	3.610536121	1.39E-15	up
LOC102181811	3.60253915	2.13E-15	up
LRP1	5.455772225	3.44E-15	up
ANXA2	2.313632918	5.41E-15	up
AKR1B1	3.547600607	5.96E-15	up
BMPR1B	3.003724641	1.08E-14	up
STOX2	3.52377255	1.72E-14	up
DCAF16	2.450336027	2.78E-14	up
LOC108633282	2.900376247	3.97E-14	up
LOC102169846	3.648236596	4.04E-14	up

FAM43A	3.664813804	8.43E-14	up
C22H3orf67	2.582839036	2.56E-13	up
PDLIM5	11.88215435	3.09E-13	up
CCND2	4.225542316	3.17E-13	up
MOB1B	2.858274804	1.76E-12	up
FXYD4	3.960079139	2.25E-12	up
CRYAB	2.61978515	2.58E-12	up
LOC102186901	2.770212038	4.38E-12	up
RAET1E	12.68453679	5.3E-12	up
LOC102185625	2.525669458	5.38E-12	up
SYT10	13.53044779	6.79E-12	up
SERINC5	3.209643546	7.18E-12	up
CLEC4E	3.763231197	7.45E-12	up
MPC1L	14.64353305	1.28E-11	up
VAV1	4.029272721	1.36E-11	up
TPM2	3.100288005	1.43E-11	up
LOC108635993	7.806669007	1.59E-11	up
LOC102174044	3.634517533	1.75E-11	up
ZC3HAV1	1.795467832	2.62E-11	up
LOC108634443	4.720511336	2.74E-11	up
PBX3	2.188449163	3.01E-11	up
HSH2D	3.50677582	3.31E-11	up

LOC102179758	2.003854883	3.91E-11	up
NPR3	11.91672398	7.5E-11	up
HOPX	4.00201075	7.69E-11	up
LOC102173176	9.99374983	9.84E-11	up
RAB31	4.725568285	1.02E-10	up
LOC102182127	5.463691647	1.09E-10	up
STXBP6	3.048202021	1.22E-10	up
IL18	3.549782766	1.36E-10	up
LOC102175315	12.52580759	1.49E-10	up
LOC108634456	2.83342619	1.72E-10	up
LOC102180110	2.873937276	1.76E-10	up
TMEM37	2.344260419	1.81E-10	up
MLLT11	2.148712024	2.71E-10	up
LOXL4	6.587969694	2.71E-10	up
POLK	2.126850192	3.01E-10	up
COTL1	1.878629235	3.61E-10	up
MDM2	1.635340914	4.16E-10	up
GABRA2	12.1773614	4.34E-10	up
EGLN3	3.045169598	4.39E-10	up
LOC102168459	3.12014881	4.54E-10	up
TMEM217	13.76495865	4.89E-10	up
LOC102186003	4.075631244	5.06E-10	up

PCSK1	3.412611593	5.8E-10	up
PNP	2.614591186	6.75E-10	up
TM4SF1	3.413517733	7.56E-10	up
BLOC1S2	1.56956817	7.69E-10	up
ADA	3.276109829	7.94E-10	up
LOC102180547	2.491913511	8.28E-10	up
KRT19	2.670971399	1.08E-09	up
BBOX1	3.813951798	1.3E-09	up
NUAK2	3.097941142	1.38E-09	up
IRX3	13.10449363	1.51E-09	up
KITLG	2.215127113	1.64E-09	up
NEFL	2.765469034	1.65E-09	up
LOC102178882	4.040145148	1.67E-09	up
LOC102179515	3.61969826	1.68E-09	up
LOC102183358	3.010302171	2.52E-09	up
LOC102173518	2.908157784	2.53E-09	up
CPO	4.019342819	2.7E-09	up
LOC102173131	14.0033841	2.76E-09	up
LOC102171823	3.141221566	2.89E-09	up
CDKL5	2.178225311	2.99E-09	up
PHLDA3	1.664346069	3.13E-09	up
EMP2	2.96674045	3.19E-09	up

SERPINI2	4.21236485	3.37E-09	up
LOC102184211	2.603112629	4.28E-09	up
HNMT	6.457360376	6.47E-09	up
NEUROD1	12.32349486	7.08E-09	up
SPTBN2	6.32666867	7.42E-09	up
LOC102176495	3.381539858	7.79E-09	up
CCNA1	2.403528157	7.87E-09	up
LOC102171918	2.491026683	8.72E-09	up
PTPN5	11.65244765	1.01E-08	up
ZMAT3	1.885327339	1.08E-08	up
BTG4	2.221768937	1.12E-08	up
FOSB	3.08284354	1.17E-08	up
DAP	1.942211875	1.45E-08	up
LOC106502282	11.41389057	1.48E-08	up
ABCA1	2.09841383	1.51E-08	up
CRIP1	2.997592945	1.73E-08	up
FAM198B	5.826882801	1.77E-08	up
LOC108634440	3.828696255	1.82E-08	up
CMYA5	6.882235744	1.84E-08	up
NDRG1	1.820585306	2.37E-08	up
GPR19	2.481290688	2.5E-08	up
EDA2R	4.857024837	2.61E-08	up

LOC102187782	12.72836391	2.65E-08	up
ALCAM	3.981237573	3E-08	up
S100A10	1.653621793	3.43E-08	up
SLC15A3	6.689879886	3.65E-08	up
DNAJC12	2.745449942	3.66E-08	up
S100A2	2.422201208	3.67E-08	up
LOC102171580	12.63789805	3.71E-08	up
FLT3	1.985127124	4.15E-08	up
LOC102169573	3.703370398	4.16E-08	up
LOC102185583	5.616135776	4.19E-08	up
C23H6orf15	13.3288446	4.29E-08	up
C5H12orf54	3.518615747	4.45E-08	up
LOC102190498	13.34778332	4.54E-08	up
CCDC168	9.073400743	4.92E-08	up
AHNAK	1.912224306	5.09E-08	up
APLP2	2.567723775	5.18E-08	up
LOC102176672	4.389399419	5.24E-08	up
MCUB	1.98559569	5.85E-08	up
TCP11L1	3.069973691	6.42E-08	up
LY6G6C	2.837777922	6.46E-08	up
KCNN1	5.107152661	8.22E-08	up
CLDN1	4.664560983	8.85E-08	up

AJUBA	2.779322992	1.03E-07	up
LOC102186225	2.221513782	1.03E-07	up
LOC102178473	2.76043122	1.2E-07	up
HSPB1	2.410502697	1.24E-07	up
LOC108634467	13.33955451	1.29E-07	up
LOC102179924	3.470966801	1.31E-07	up
TACR3	6.220519652	1.37E-07	up
RTN2	2.31432992	1.49E-07	up
SCRN1	2.862216675	1.49E-07	up
TMEM88	3.309968332	1.6E-07	up
PYROXD2	5.430450333	1.63E-07	up
LOC108637498	5.741405681	1.73E-07	up
S100A11	2.085574851	1.78E-07	up
CDC20B	3.591802494	1.89E-07	up
PTPRB	3.043516924	1.91E-07	up
TACSTD2	1.587461731	1.94E-07	up
LOC102169861	4.434198294	2.04E-07	up
TEX11	4.801050293	2.06E-07	up
CPEB1	2.789501971	2.22E-07	up
SPAG1	1.809548504	2.33E-07	up
CFAP44	2.88593987	2.34E-07	up
CIDEC	3.460985866	2.64E-07	up

AIG1	6.005209595	2.64E-07	up
HOXA3	10.67288201	2.65E-07	up
S100A5	2.31555433	2.66E-07	up
GCA	4.498281369	2.8E-07	up
INA	2.437457153	2.95E-07	up
LOC108636742	3.760475136	3.02E-07	up
GADD45B	2.597388039	3.03E-07	up
PCSK5	2.876473606	3.04E-07	up
MRAP2	3.466824118	3.18E-07	up
CEMIP	3.661993337	3.36E-07	up
LOC108637776	2.227648446	3.38E-07	up
CLEC1A	2.458921604	3.66E-07	up
BPIFC	4.948666838	3.92E-07	up
FXYD6	1.844753646	4.04E-07	up
KRT1	12.23104613	4.58E-07	up
S100A3	4.417105369	4.68E-07	up
KRT5	3.219174842	5.39E-07	up
COL17A1	2.762145682	5.46E-07	up
NLRP3	6.342441988	5.76E-07	up
LOC102180587	2.000624621	7.16E-07	up
LSAMP	5.571573364	7.25E-07	up
LOC108637372	4.452922993	7.46E-07	up

PLS3	2.115579909	7.53E-07	up
TM6SF1	3.323274518	7.71E-07	up
LOC102176170	4.383416894	7.99E-07	up
PAQR8	3.679974719	9.11E-07	up
PDPN	1.70167568	9.35E-07	up
LOC102178315	1.889446113	9.36E-07	up
KLHL28	1.778674682	9.76E-07	up
LOC102168959	1.550254706	1E-06	up
PCDH17	2.918818319	1E-06	up
LAMC1	1.254820406	1.05E-06	up
TNS1	2.881346084	1.08E-06	up
SPTBN1	1.755132683	1.08E-06	up
MYLIP	2.224593512	1.15E-06	up
PLAC8	1.882097563	1.16E-06	up
SLC6A17	2.260290809	1.17E-06	up
MUC13	3.14593348	1.21E-06	up
TLR8	5.587317292	1.34E-06	up
SIAH1	1.785229414	1.4E-06	up
LOC108637665	4.486498246	1.46E-06	up
DNAH3	2.260567254	1.47E-06	up
MYRFL	6.639792274	1.48E-06	up
TNFRSF13B	2.994986166	1.58E-06	up

MORF4L2	1.269413936	1.59E-06	up
MYOF	1.537003284	1.61E-06	up
MZB1	6.307520251	1.74E-06	up
LOC102184551	2.34969768	1.82E-06	up
SLC7A2	2.845775818	1.87E-06	up
LOC102178584	3.693200299	2.04E-06	up
SORBS3	2.668587652	2.08E-06	up
DLX5	1.785472021	2.11E-06	up
LOC102168680	1.300637038	2.16E-06	up
LOC102175701	1.724316202	2.17E-06	up
FILIP1	1.48824706	2.19E-06	up
LOC102177878	4.79904715	2.2E-06	up
IFI30	2.490605624	2.25E-06	up
COLEC12	2.519057511	2.32E-06	up
LOC102179881	2.511125944	2.37E-06	up
TUFT1	1.384863579	2.64E-06	up
GCNT4	3.73034855	2.66E-06	up
MTERF2	1.430965341	2.76E-06	up
C16H1orf116	1.55318079	2.76E-06	up
LRRC66	2.635558232	2.85E-06	up
GUCY2C	2.737010197	2.88E-06	up
GUCA1A	1.220599012	2.94E-06	up

IER3	1.487174137	2.97E-06	up
IL1A	2.722229226	3.11E-06	up
LOC102180339	3.190169922	3.14E-06	up
BRWD3	1.62801437	3.19E-06	up
CBLN2	3.119205912	3.26E-06	up
LMCD1	4.205092914	3.37E-06	up
ZNRF2	1.896336374	3.4E-06	up
LOC108634083	1.396450632	3.45E-06	up
ARRDC2	2.555244925	3.55E-06	up
LOC102187869	5.172586611	3.62E-06	up
LOC102173807	1.922284571	3.73E-06	up
GADD45A	1.257952036	3.87E-06	up
LOC108634365	13.44146066	3.95E-06	up
PRTFDC1	4.677615816	4.18E-06	up
MME	1.964909813	4.29E-06	up
M1AP	2.616261065	4.32E-06	up
LOC102175234	2.346054334	4.7E-06	up
ITPR1	2.199718609	4.71E-06	up
FHIT	3.181737478	4.74E-06	up
LOC108636061	2.820555815	4.87E-06	up
TIMP1	1.551534133	5.03E-06	up
SIM1	4.608297596	5.34E-06	up

SGMS2	1.678840573	5.37E-06	up
HMOX1	2.591907369	5.45E-06	up
STARD13	4.745338662	5.57E-06	up
ADD3	1.552598804	5.69E-06	up
ADGRL3	5.922258369	5.73E-06	up
SALL1	5.345803695	5.8E-06	up
LOC102172184	12.83292113	6.51E-06	up
LOC106503869	5.428245802	6.69E-06	up
LOC108637519	3.432252525	6.76E-06	up
LOC106502109	2.337034784	7.12E-06	up
RCAN1	5.229578675	7.52E-06	up
OLR1	4.166105695	7.56E-06	up
SDC2	2.907770961	7.77E-06	up
LOC106502191	13.83513781	7.79E-06	up
NTAN1	1.283887078	8.12E-06	up
SH3BGRL3	1.698840048	8.17E-06	up
APELA	3.213033223	8.57E-06	up
MYH11	2.081461183	8.63E-06	up
OSBPL3	1.780782221	9.04E-06	up
LOC102189031	3.029151456	9.57E-06	up
BCAR3	2.401993801	1.05E-05	up
FAM169B	1.765617039	1.06E-05	up

IER5	1.705786236	1.1E-05	up
TIMP2	2.831407721	1.12E-05	up
DNAJC6	2.753212164	1.16E-05	up
EPB41L2	1.523125535	1.19E-05	up
CREB3L1	3.058776246	1.19E-05	up
MORC4	2.671642214	1.2E-05	up
PPM1D	1.662521992	1.27E-05	up
LOC108638494	4.432383875	1.28E-05	up
LOC108635643	4.088899225	1.31E-05	up
LOC108634189	2.850966327	1.39E-05	up
GPR84	2.296501233	1.44E-05	up
TNFRSF17	3.153559361	1.46E-05	up
SPINK7	2.978804933	1.49E-05	up
NKD1	4.828651534	1.53E-05	up
EPAS1	1.656043378	1.58E-05	up
UNC119	1.943688604	1.59E-05	up
AMMECR1	1.567286235	1.64E-05	up
HK1	1.40427717	1.65E-05	up
GALM	1.251431146	1.68E-05	up
CXHXorf66	6.146507297	1.72E-05	up
LOC102177904	4.121245582	1.77E-05	up
HSP70.1	2.295333874	1.79E-05	up

UGP2	1.124706859	1.82E-05	up
SPATS2L	2.135287564	1.89E-05	up
LOC108636716	5.677566772	1.9E-05	up
TDRD10	5.256151555	1.93E-05	up
PERP	1.2304598	1.98E-05	up
LOC102187762	2.847491635	1.99E-05	up
LOC102182927	4.167547613	1.99E-05	up
IL2RG	5.016965516	2.02E-05	up
SFN	2.31416439	2.02E-05	up
LOC108638538	2.267649395	2.06E-05	up
PRSS33	12.93809033	2.13E-05	up
CDK20	2.145746969	2.17E-05	up
ITGA3	1.891082369	2.19E-05	up
MMP2	3.504480473	2.2E-05	up
WFDC2	4.266034472	2.21E-05	up
RLIM	1.366710912	2.25E-05	up
LOC102190263	2.14440568	2.3E-05	up
SERTAD4	1.967643824	2.34E-05	up
CD99	1.027448543	2.34E-05	up
GDPD1	1.58619629	2.37E-05	up
FKBP10	5.018382206	2.39E-05	up
EPHA4	2.734194362	2.41E-05	up

LOC106503797	4.561635632	2.43E-05	up
DFNA5	4.078773124	2.44E-05	up
LOC102176710	4.69699529	2.47E-05	up
LOC102188781	4.351255867	2.55E-05	up
TPBG	4.30659906	2.73E-05	up
EGF	5.116838431	2.78E-05	up
ARID5B	2.827657599	2.89E-05	up
AGBL2	3.885686978	2.93E-05	up
SLC6A4	2.561439683	2.97E-05	up
MAP1LC3A	1.54137664	2.98E-05	up
FOSL1	1.726624865	3.02E-05	up
RECK	1.9474846	3.05E-05	up
USP32	1.63687322	3.29E-05	up
BGN	12.14808673	3.36E-05	up
LRMP	4.399082278	3.44E-05	up
LOC102186863	3.691338113	3.7E-05	up
LOC106503091	1.732963824	3.75E-05	up
LAMA1	1.577502283	3.83E-05	up
LRAT	1.747402714	3.87E-05	up
LOC102169116	3.574453755	3.93E-05	up
SLC25A21	2.44108678	3.99E-05	up
N4BP2L1	1.815584282	4.01E-05	up

ELF1	1.492941042	4.02E-05	up
BMP4	1.559232324	4.31E-05	up
NMNAT2	4.009662972	4.39E-05	up
KIAA0408	4.806210934	4.43E-05	up
CPEB4	1.158426767	4.47E-05	up
RNF11	1.372883662	4.52E-05	up
PAX6	2.353370776	4.7E-05	up
DAB1	3.608471235	4.71E-05	up
XIRP2	9.669074023	4.72E-05	up
GML	1.979030044	4.79E-05	up
MID1	2.514838179	4.81E-05	up
SLC7A6	1.533031934	4.94E-05	up
RNF217	11.19515276	4.96E-05	up
PLIN3	1.875889835	4.96E-05	up
LOC102188015	2.153701352	5.02E-05	up
CNNM2	1.446622752	5.08E-05	up
HMGCR	1.099780545	5.16E-05	up
ST3GAL1	4.004529043	5.17E-05	up
LIFR	1.855825445	5.18E-05	up
GM2A	2.058468925	5.32E-05	up
LOC102187632	2.495899612	5.4E-05	up
KRT7	12.12771925	5.5E-05	up

PMP22	3.744588819	5.58E-05	up
S100A4	1.92227043	5.63E-05	up
FSD2	4.330872442	5.65E-05	up
LYPD1	2.087453713	5.69E-05	up
RGS16	1.724830633	5.83E-05	up
TCEA3	4.999951561	5.95E-05	up
CGA	13.21018681	5.97E-05	up
NRP2	2.358938893	6.11E-05	up
ARHGEF6	2.612676261	6.13E-05	up
GSS	1.283777843	6.31E-05	up
PRDX1	1.05700355	6.43E-05	up
DYNLRB2	1.857348923	6.46E-05	up
HTR1B	3.777799526	6.48E-05	up
KCTD12	2.369392516	6.61E-05	up
SUSD4	4.764840427	6.77E-05	up
DCBLD2	2.812966093	6.81E-05	up
LOC108635488	2.02517122	6.88E-05	up
SLC41A2	1.653507271	6.91E-05	up
DPYSL2	2.070663648	6.96E-05	up
AAMDC	1.566784782	7.36E-05	up
LGALS7	2.602557756	7.71E-05	up
MEIS2	2.090343117	7.81E-05	up

FOXO4	2.742230088	8.06E-05	up
ECE1	3.066615323	8.22E-05	up
TNMD	3.058652623	8.23E-05	up
CDH13	2.662695373	8.23E-05	up
LOC102171160	1.863220292	8.26E-05	up
LITAF	1.053317897	8.26E-05	up
LOC102177879	3.534468186	8.42E-05	up
ARMCX3	2.647032845	8.48E-05	up
MAPK13	1.890762311	8.71E-05	up
GIP	2.407752369	8.89E-05	up
DYNLT3	1.389273359	8.98E-05	up
SLC30A1	1.392108589	9.08E-05	up
PPM1N	1.352718861	9.22E-05	up
GMNC	4.914924126	9.34E-05	up
RTN4	1.936161959	9.49E-05	up
VCAN	2.254350377	9.82E-05	up
ASB11	1.863892045	9.87E-05	up
LOC102180938	3.955442176	0.000106	up
LOC102176862	1.912727404	0.000109	up
WWC3	1.744141652	0.000109	up
LOC106502633	4.157156367	0.00011	up
GNGT1	1.917917389	0.000115	up

WEE1	1.264950043	0.000115	up
SH3RF2	4.52852421	0.00012	up
ELF3	2.664950276	0.00012	up
COL12A1	1.996595874	0.000121	up
ATOH1	5.372728403	0.000126	up
SUPT4H1	1.040337949	0.000127	up
ZNF385B	5.662494064	0.000127	up
ZC2HC1A	1.700455892	0.000128	up
STK10	2.706677703	0.000128	up
ANKRD44	2.207111945	0.000129	up
NPPA	2.951132374	0.00013	up
MYO10	2.164427138	0.000134	up
LOC108634969	2.946281368	0.000137	up
PCBP4	2.582746214	0.000139	up
LOC108634205	3.950692562	0.00014	up
PEA15	1.397150197	0.000149	up
LOC106502711	1.268375147	0.00015	up
LOC102171344	1.896326991	0.000153	up
ZP2	2.310485437	0.000153	up
SH2D4B	4.170966964	0.000156	up
DFFA	1.280122082	0.000156	up
DYRK3	1.620525574	0.000157	up

PAIP1	1.009212948	0.000159	up
STOX1	2.308314297	0.000162	up
LOC102180595	1.646434573	0.000162	up
COL5A3	2.082486686	0.000163	up
LOC102180840	3.219092185	0.000165	up
CCND1	1.827900163	0.000166	up
IL4R	1.715035895	0.000166	up
LOC102173020	1.859358511	0.000167	up
FRMD6	1.777371718	0.000169	up
BSPRY	3.187216238	0.00017	up
LOC108638455	2.25953771	0.000173	up
LOC102180074	1.112501512	0.000177	up
FOSL2	2.062215052	0.000177	up
ACSL6	1.716175041	0.000179	up
TP53INP1	1.138409162	0.00018	up
CDC25B	1.186164003	0.000181	up
EPHA2	2.44290025	0.000182	up
SLC16A11	2.327676213	0.000183	up
CSNK1G1	1.451775601	0.000184	up
TSC22D2	1.405302018	0.000184	up
AMIGO3	2.745661733	0.000186	up
CAB39L	1.143849172	0.000187	up

SERPINI1	1.636929864	0.000188	up
PTPN14	1.336070187	0.000188	up
GJC1	1.607203935	0.000189	up
LOC102178042	2.11551045	0.000189	up
SHISA2	1.717587164	0.000191	up
DSG3	2.733608515	0.000192	up
SPOPL	1.251311504	0.000197	up
EFR3B	2.461635135	0.000198	up
INPP1	1.868697457	0.000198	up
PRICKLE1	1.41623408	0.0002	up
MGARP	1.91433873	0.000201	up
LOC102176775	1.377093753	0.000209	up
GPRC5A	2.44558481	0.000213	up
FOXO1	1.223447736	0.000215	up
SLC10A1	3.491763517	0.000215	up
NPTN	1.151738302	0.000215	up
IL1RAP	2.827208793	0.000217	up
UNC5D	3.938802472	0.000217	up
IL6	1.233652946	0.00022	up
LOC106502520	4.603368181	0.00022	up
FBXO40	2.211580278	0.000221	up
LOC102173519	2.165943832	0.000222	up

FOLR1	1.073706874	0.000223	up
LOC102172474	1.672442226	0.000225	up
LRRK1	2.84790831	0.000226	up
ARNTL	1.845841079	0.000227	up
SLC20A1	1.000131911	0.000228	up
LOC102168946	4.994756751	0.000228	up
RGSL1	2.186847328	0.000229	up
LOC102184534	1.327823959	0.000231	up
LOC108638073	1.812851614	0.000232	up
TIFA	2.687353605	0.000234	up
C16H1orf105	1.402149826	0.000234	up
PEG10	2.813791749	0.000236	up
ARHGAP30	4.61869942	0.000237	up
CKS1B	1.013947042	0.00024	up
DOCK4	1.481230678	0.000244	up
STK38L	1.331003621	0.000245	up
LMO3	3.072380299	0.000246	up
LOC108635411	2.412395611	0.000248	up
AREG	3.693606191	0.00025	up
TMEM106A	2.225295028	0.000251	up
FAM177B	2.770656937	0.000256	up
CTGF	1.431283971	0.000258	up

LOC102179835	4.929933113	0.000262	up
ANO6	1.260429685	0.000267	up
SORBS2	4.849752928	0.000269	up
LOC106501722	1.155770083	0.000271	up
ATXN1	4.937846757	0.000275	up
TMEM159	2.73782014	0.000286	up
LOC108638027	2.362937901	0.000289	up
SLC40A1	2.623572854	0.00029	up
GPR37	4.5905131	0.000291	up
GUCA2A	1.442740981	0.000291	up
SFXN5	3.070433819	0.000292	up
SRGAP1	1.590437895	0.000293	up
HTRA4	4.493650319	0.000295	up
LOC102190382	3.304632416	0.000302	up
LOC102184972	2.640331954	0.000306	up
TMEM176B	3.121801348	0.000308	up
DDIT4	1.509467409	0.000311	up
FABP3	1.234891303	0.000311	up
LAMA3	2.695587747	0.000316	up
LOC102182304	2.467240427	0.000319	up
TANC1	1.247740445	0.000322	up
LRRC8D	1.418352791	0.000325	up

TRIM59	1.578654009	0.000327	up
GTF2H5	1.026420223	0.000328	up
ELOVL7	1.522196545	0.000331	up
JAG1	1.848595233	0.000332	up
USP13	2.563510271	0.000334	up
COL6A6	4.616066853	0.000337	up
MYO1B	1.286089383	0.000347	up
NFATC3	1.275803969	0.000348	up
PRM2	2.588435567	0.000363	up
KRT18	1.421445426	0.000367	up
SERGEF	2.217461054	0.000368	up
RGL1	2.83971285	0.000368	up
TP53INP2	1.399648758	0.00037	up
KERA	3.753639523	0.000385	up
FGF13	3.531033683	0.000387	up
SWAP70	1.11185367	0.00039	up
LOC108638473	2.475630268	0.000392	up
MUC15	3.977825679	0.000395	up
B2M	1.25966217	0.000403	up
MYO1E	1.182068811	0.00042	up
PLEKHB1	2.751784173	0.000421	up
BANF2	2.233077347	0.00043	up

EGR1	2.772522509	0.000433	up
ENPP2	1.658359431	0.000443	up
CDC42EP3	2.562854466	0.000445	up
ALOXE3	3.516190575	0.000445	up
BCHE	4.296316511	0.000447	up
MAPK10	3.592709497	0.00045	up
CDK2AP2	1.321871662	0.000452	up
BIK	1.858926987	0.000453	up
KIAA1217	1.022995014	0.000455	up
JADE3	1.607375578	0.000458	up
CYR61	1.241662081	0.000462	up
MBNL2	4.790488385	0.000462	up
TNS2	2.394473909	0.000463	up
CTSL	1.529379713	0.000464	up
PYGL	2.152920094	0.000465	up
LOC102174926	3.563527105	0.000467	up
SLC12A7	2.024338829	0.000473	up
ADAMTS1	3.016351295	0.000476	up
INO80C	1.154589363	0.000477	up
SPAG17	3.10450099	0.000478	up
CDKL1	2.320815106	0.000482	up
DPF3	1.686805346	0.000485	up

PDE3B	2.641759488	0.00049	up
LOC108634850	4.248661483	0.000491	up
SPARC	1.131273039	0.000495	up
RGAG1	4.327722142	0.000497	up
LOC102168424	2.668523058	0.000497	up
NECTIN4	3.468305997	0.000499	up
PARM1	2.349635556	0.000502	up
LOC106501876	6.257457226	0.000502	up
LOC102181869	1.704462744	0.000511	up
LAMA5	2.947688314	0.000511	up
GDF3	1.357933428	0.000523	up
LOC106501929	1.301633781	0.000523	up
LOC102178093	2.456511864	0.000524	up
RAB27A	1.416879288	0.000525	up
EPHB4	3.54966177	0.000527	up
INCA1	2.120639051	0.000532	up
ZFPM2	3.385417646	0.000532	up
VAMP5	1.901928603	0.000536	up
MFSD2A	1.929688065	0.00054	up
LOC108633414	2.421486873	0.000541	up
LOC102185494	1.734554804	0.000545	up
PRDM1	4.639894739	0.000552	up

LOC108636407	1.568951322	0.000554	up
LPIN1	1.75186555	0.000561	up
PROB1	3.721523985	0.000561	up
MSMO1	1.076267219	0.000565	up
WNT6	2.047388687	0.000574	up
TRIB2	2.430988872	0.000575	up
ANXA1	1.79606113	0.000577	up
TACC2	2.220688395	0.000586	up
TNNT1	1.967389005	0.000592	up
HMMR	1.066500554	0.000593	up
FABP5	1.761773913	0.0006	up
PPFIBP1	1.926322622	0.000605	up
LOC102177846	2.334878097	0.000611	up
LOC106503010	3.635471875	0.000611	up
TRIM38	2.621741743	0.000612	up
LOC102191197	2.834914996	0.000613	up
LOC108633215	1.429510201	0.000619	up
ERBB4	3.458042753	0.00062	up
LOC102191731	1.45942269	0.000623	up
LOC102191603	1.313665099	0.000627	up
SLC29A1	2.060578737	0.000631	up
STK26	2.139654304	0.000631	up

BEND4	2.195550918	0.000637	up
SHCBP1L	2.767523293	0.000644	up
LOC108633254	1.278077683	0.000647	up
FAM46C	1.83889834	0.000668	up
PRKD2	3.004396831	0.000669	up
SOX6	1.435048332	0.000685	up
KPNA7	1.028505639	0.000688	up
LOC108636579	1.473663591	0.000691	up
ABTB2	2.760261739	0.000691	up
CD70	2.390363617	0.000696	up
RNF43	2.772516065	0.000698	up
TRAK1	1.559102119	0.000698	up
FBXL17	1.466621718	0.000708	up
NEB	1.315821917	0.000717	up
LOC102173645	1.098001796	0.000722	up
PDIA4	1.885115285	0.000722	up
STEAP2	2.671305019	0.000731	up
LOC108637986	1.561471333	0.000736	up
ID2	1.003485121	0.000737	up
LBH	3.27398003	0.000744	up
AUTS2	1.498494147	0.000755	up
PRDX6	1.008822576	0.000768	up

CD55	1.267350736	0.000769	up
RAP2A	1.239038659	0.00079	up
BHLHE22	4.243462881	0.000825	up
LOC102177005	1.018537096	0.000839	up
SAMD4A	2.498869051	0.000843	up
LOC102189655	1.941382592	0.000847	up
LOC102180412	1.479894367	0.000853	up
IL20RA	2.568621771	0.000859	up
LDB3	1.410879941	0.000859	up
CCDC160	1.859702517	0.000863	up
PLAC9	2.076864495	0.000866	up
USHBP1	4.559015649	0.000875	up
LOC108635092	2.447193946	0.000887	up
IL10RA	1.885055288	0.000891	up
LOC108636602	4.314896563	0.000904	up
ACSF2	3.051599212	0.000905	up
CHST15	1.895640572	0.000916	up
CREB3L2	2.242010615	0.000921	up
CYB5A	1.14595073	0.000922	up
CNTNAP2	2.303629521	0.000923	up
LPP	1.306919025	0.000934	up
CDK14	1.519459821	0.000936	up

ZHX1	3.349623368	0.000936	up
IGSF9	2.847989982	0.000954	up
LOC108638078	1.980379383	0.001008	up
TNFAIP8	1.656013091	0.001012	up
MYBL1	1.60477439	0.001015	up
FAM129A	2.383826102	0.001023	up
ZAR1L	1.812545715	0.001028	up
SPRY3	2.661958091	0.001033	up
EPHX2	3.253065928	0.001037	up
SNX25	2.090327719	0.001042	up
EFHB	1.285925497	0.001043	up
BIRC3	1.052444545	0.001046	up
LOC102184867	3.639823601	0.001052	up
SLC16A4	1.734109247	0.001065	up
RASGRP3	1.65948953	0.001069	up
OPHN1	4.408430585	0.00107	up
ADM	1.507110416	0.001092	up
STON1	1.134834173	0.001093	up
PTPRK	2.288027246	0.001093	up
INSL6	3.57911201	0.001101	up
HNRNPLL	2.036245189	0.001104	up
CAPN8	2.60974932	0.001104	up

VIM	2.174694015	0.001108	up
ITGA2B	1.688553397	0.001109	up
ABLIM1	1.598241336	0.001121	up
FAM13A	2.38388528	0.001124	up
CAV1	4.202783949	0.001132	up
LOC108634362	2.699772993	0.001141	up
CCNJL	4.245257751	0.001144	up
NRCAM	3.320255623	0.001145	up
FAM109B	1.443880817	0.00116	up
LOC108635916	3.077492184	0.001165	up
GLIS3	1.919031735	0.001169	up
LOC102189920	2.650092832	0.001218	up
FRMD4B	1.266862019	0.001219	up
SLC22A16	3.345609903	0.001227	up
LOC108637112	3.064238789	0.001228	up
DLX4	2.095959904	0.001245	up
ERI3	1.291673939	0.001246	up
RBP4	3.090642085	0.001249	up
LOX	2.617467344	0.001263	up
KRT8	1.127531304	0.001267	up
PLEKHO1	2.439770666	0.001271	up
SMPD3	1.121769466	0.001277	up

LOC102171358	1.081776432	0.001297	up
FAM107B	1.755450286	0.001298	up
LOC102191717	6.018288656	0.001305	up
LOC102179277	1.141010594	0.001307	up
CCR7	4.995045239	0.00132	up
DRD1	3.835956858	0.00132	up
LOC102172183	1.217285918	0.001329	up
LOC108637745	2.390888349	0.001332	up
LOC108637960	1.285154682	0.001336	up
ELAVL2	1.208089129	0.001339	up
ABCC6	3.319445774	0.001343	up
THEGL	3.643567352	0.001347	up
ZBTB33	1.188773874	0.001353	up
LOC106501935	2.524159712	0.001371	up
PLCXD2	2.213146921	0.001378	up
DPP4	3.617924183	0.001384	up
TMEM139	1.318707712	0.001392	up
LOC108633237	3.198308245	0.00143	up
FADS1	1.394743084	0.001433	up
FAM159B	3.517367478	0.00144	up
TEX14	2.60536217	0.001462	up
SESN1	1.271020536	0.001466	up

LOC102169815	1.27269467	0.001472	up
SCARB1	1.49884949	0.001476	up
C16H1orf101	3.391226453	0.001479	up
KRT80	4.120791718	0.00148	up
PLK2	1.143579247	0.001485	up
LOC102176799	3.006344804	0.001486	up
LOC102184512	1.56299276	0.001488	up
TMEM40	2.035742964	0.001498	up
LOC108636832	1.5549791	0.001499	up
MACROD1	1.891057998	0.0015	up
SCN8A	1.329994048	0.001517	up
GFPT2	1.791007795	0.001525	up
FAM83C	1.968210617	0.001525	up
SBK1	1.669793237	0.001526	up
LOC102170513	1.797382258	0.001528	up
NLRP14	2.073416842	0.001534	up
ACTC1	4.542522471	0.001535	up
LOC102186135	2.498782042	0.001542	up
PRKAR2B	1.776157703	0.001544	up
ENPP1	1.95817989	0.001547	up
SEMA4F	1.915787255	0.001547	up
C5H12orf75	4.253738171	0.001556	up

HMCN1	1.907567287	0.001563	up
IGF2BP3	1.254540212	0.001579	up
PAH	1.901850663	0.001583	up
LOC108635155	1.69829684	0.001605	up
EFHC1	3.108888295	0.001613	up
FRY	2.042917758	0.001617	up
STAR	1.859615789	0.001621	up
PID1	2.492481087	0.001621	up
TRIM63	1.822810142	0.001622	up
LOC102175278	3.42016341	0.001626	up
STARD10	2.013513908	0.001627	up
HAND1	1.395849349	0.001648	up
LRP4	2.332135504	0.001671	up
NUDT22	1.416321379	0.001719	up
LOC108636128	1.551810891	0.001725	up
BHLHE40	1.835141075	0.001734	up
CT83	2.419104253	0.00175	up
LOC102188976	2.683941969	0.001751	up
SQLE	1.317974671	0.00177	up
BCL2L10	1.7429611	0.001771	up
BNC2	1.598379629	0.001782	up
TTC38	1.43980785	0.001797	up

LOC108637403	3.166614597	0.001812	up
SPSB1	2.450520426	0.00184	up
OSBPL10	2.709402828	0.001845	up
DCHS2	3.121265119	0.00185	up
LOC102172037	2.660366729	0.001855	up
IKZF2	2.270187867	0.001859	up
DLST	1.577258533	0.001868	up
LOC102182562	1.013794329	0.001875	up
ZNF596	1.482261934	0.001911	up
WNT2B	2.397426211	0.001923	up
SP4	2.170797627	0.001923	up
FAS	1.190279639	0.00197	up
LCA5	3.284349956	0.001982	up
SCAPER	1.262398883	0.001983	up
SLC35A1	1.33900513	0.002018	up
AMPH	2.953142472	0.002038	up
LOC108635252	2.434571913	0.002052	up
ZNF503	2.826626422	0.002054	up
PDE7A	1.338644209	0.002075	up
CHAC2	2.145270454	0.002079	up
FAM65B	1.521771246	0.002082	up
CTNNA3	2.814597655	0.002091	up

RRAGB	1.746490342	0.002094	up
LOC106503278	3.817978065	0.002099	up
EYA1	1.119220826	0.002112	up
LRRC15	2.57454478	0.002116	up
GLI3	1.39283114	0.002125	up
B4GAT1	1.460002688	0.002137	up
ADAM19	1.391086916	0.002148	up
SLC22A4	1.268715925	0.00215	up
ITGA4	2.90319095	0.002158	up
DAPK1	1.2523258	0.002158	up
DTNA	3.022308597	0.002159	up
AASS	1.135568892	0.002159	up
PCDH1	1.232077163	0.002163	up
FILIP1L	2.164426631	0.002166	up
LOC108633781	1.345918509	0.002185	up
LIMCH1	2.643828723	0.002188	up
LOC102174125	1.859719782	0.002205	up
SLC35E4	2.255980607	0.002212	up
BIN3	1.06448078	0.002238	up
SMOC1	3.300980384	0.002275	up
LOC102176721	2.259319178	0.002277	up
LZTFL1	1.116464576	0.002277	up

LRP2	1.071639628	0.002278	up
EPHX1	1.596142038	0.002327	up
SGCB	3.328560504	0.002333	up
MISP	1.503893073	0.002334	up
BNC1	1.989724252	0.002337	up
MSX1	2.351698331	0.002396	up
DCLRE1B	1.144758406	0.002401	up
TUBB6	1.268568254	0.002406	up
PAG12	3.343239533	0.002419	up
ARHGAP1	1.17686422	0.002423	up
TPCN1	1.668794719	0.002454	up
PTPRF	1.434571331	0.002462	up
FSCN1	2.440883671	0.002486	up
C5H12orf60	2.436842861	0.002513	up
DNASE1L3	3.494192647	0.00255	up
CCL25	2.2001488	0.002574	up
DYX1C1	1.527832589	0.00261	up
LOC102180576	1.766737375	0.002615	up
LOC102191590	2.903210117	0.002618	up
CFAP126	3.00245221	0.002624	up
ADGRB3	1.781022611	0.002642	up
C14H8orf89	1.912470823	0.002685	up

CNTROB	1.632030978	0.002705	up
SH3BGRL	1.985204766	0.00272	up
VGLL3	2.19414118	0.002732	up
PYGO1	1.01043274	0.002734	up
SEPT10	2.190818638	0.002735	up
NEDD4	1.667098317	0.00274	up
ADGRL2	1.619354106	0.002742	up
GLS	3.297390432	0.002753	up
LOC102171053	2.149866177	0.002765	up
CA12	2.107683917	0.002766	up
ZNF879	1.815106653	0.002768	up
LOC102188617	4.578742588	0.002784	up
LOC102190811	2.126702616	0.002788	up
SH3BP5	2.679114809	0.002799	up
FAM13C	2.231197908	0.00284	up
PTER	1.110619446	0.002878	up
PKM	1.126208581	0.002901	up
SAT1	1.448923335	0.002906	up
LOC108634342	2.475154917	0.002913	up
SPTAN1	1.063566889	0.002922	up
LOC102178444	2.328076953	0.002948	up
LRRC1	1.123587664	0.002956	up

GRP	2.286881748	0.002962	up
SMPD1	1.403198343	0.003029	up
LOC106503025	2.033043276	0.003065	up
NOV	2.631512072	0.003068	up
NUAK1	3.779238964	0.003085	up
RICTOR	1.013472785	0.003097	up
PHLDA2	3.317840719	0.003133	up
PTN	2.662905687	0.003137	up
ADGRF1	2.877632189	0.003153	up
TK1	1.294402903	0.003173	up
TMEM65	1.046676538	0.003184	up
EFNA5	2.384263622	0.003187	up
HEG1	1.675872939	0.003218	up
FABP7	1.14336669	0.003221	up
KIAA0141	1.488125209	0.003263	up
GNGT2	1.125888452	0.003273	up
LOC108634100	2.140445031	0.003291	up
LOC108638589	1.335197543	0.003322	up
SORCS3	2.403128389	0.003324	up
COL14A1	1.889279887	0.003332	up
LY6G6D	2.547230422	0.003342	up
PRPS2	1.236286177	0.003346	up

RPS6KA6	1.718266847	0.003361	up
CDCP1	2.391843775	0.00339	up
LOC106501961	3.096165231	0.003412	up
ITPRIP	1.995857535	0.003412	up
SLC7A8	1.285279945	0.00345	up
NXNL2	2.217316478	0.003503	up
GDF9	1.489755783	0.003504	up
SCNN1G	3.141730841	0.003515	up
KCNMB4	3.030726363	0.00352	up
WNT5B	2.214238213	0.003536	up
LOC102177258	2.747820478	0.003546	up
SLC26A2	1.258279482	0.00356	up
FGGY	2.635627113	0.00357	up
KIF21A	1.637850137	0.003644	up
TRAF3IP3	2.238719868	0.003652	up
DOK5	2.560680207	0.00366	up
RASSF3	1.599527233	0.003672	up
NOSTRIN	2.529154873	0.003701	up
TCEAL9	1.638419307	0.003708	up
LOC102173120	1.156869548	0.00374	up
NFIX	1.844875641	0.003751	up
LOC102188626	2.98605882	0.003756	up

PAQR5	2.147169185	0.003774	up
TLDC1	3.588400681	0.0038	up
DUSP10	2.553568557	0.003826	up
CHRNA3	1.618653728	0.003834	up
PLEKHA7	1.418842212	0.003862	up
CHGB	3.073464008	0.003866	up
HEXIM1	1.305021076	0.003937	up
RAP2C	1.35687903	0.003947	up
NPPB	1.659499816	0.003952	up
ARHGEF12	1.015982473	0.003957	up
LOC102185239	2.355682734	0.003965	up
PHACTR2	1.839399655	0.003979	up
CLVS1	3.793098861	0.004015	up
GSTO2	2.975991322	0.004015	up
LOC102190090	2.065979158	0.004035	up
ITPKB	2.433168732	0.004042	up
MAP3K5	2.620458086	0.004091	up
KCNMA1	1.486794372	0.004101	up
FAM117B	1.028633217	0.004107	up
MPP7	1.537161225	0.004107	up
C18H16orf74	1.178795134	0.004116	up
MAP1A	1.350111847	0.004117	up

ANKS1B	3.3247833	0.004127	up
TTYH3	2.269777941	0.004157	up
ALOX12	3.088576231	0.004171	up
PWWP2B	4.016757051	0.004176	up
SLC39A8	1.03879971	0.004205	up
LOC102172897	1.78785413	0.004206	up
PDK3	1.156704308	0.004219	up
HMGCS2	1.794661317	0.00424	up
NUDT13	2.058598056	0.004266	up
LOC108633300	3.191390339	0.00427	up
ABRACL	2.791139483	0.004279	up
LOC102191333	2.555047849	0.004321	up
SERHL2	1.903456177	0.004452	up
FAM189A2	1.498473001	0.00447	up
LOC102172244	2.953168292	0.004473	up
EPHX3	2.006155772	0.004564	up
KRT14	1.584201141	0.004568	up
TNFAIP2	2.97716733	0.004594	up
SCEL	2.902277315	0.004608	up
ZDBF2	1.576499735	0.004615	up
ABI2	2.204621594	0.004651	up
FLNC	3.730234472	0.004659	up

GPA33	2.92122971	0.004673	up
PRSS23	1.072789865	0.004686	up
MAPT	1.725314121	0.004687	up
TRIM55	2.439821071	0.004711	up
SPDYC	1.491196757	0.004789	up
ARHGDIB	1.124282344	0.004804	up
ORAI2	1.63484256	0.004818	up
PDCD4	1.058697527	0.00482	up
ZCWPW1	1.639088517	0.004824	up
PPP2R2B	1.880310967	0.004832	up
CACNA2D1	1.191924369	0.004909	up
TMTC4	3.126948296	0.004951	up
THRSP	4.634857388	0.004967	up
ASIC1	2.94897685	0.004972	up
LOC102172084	1.265754056	0.004987	up
TRPM3	1.620885605	0.005066	up
CTSB	1.193887434	0.00512	up
C23H6orf25	1.858966915	0.005123	up
HSPG2	2.770849237	0.005128	up
ALPK1	2.26513231	0.005134	up
ZFP36L1	1.223020671	0.005138	up
LOC102184420	1.74878233	0.005145	up

LOC108635630	3.649400407	0.005167	up
LOC102188340	3.819255221	0.005239	up
LOC102184887	1.486435042	0.005268	up
ATP1B2	1.437819179	0.005272	up
PTPRJ	1.315030852	0.005278	up
LOC102191152	3.167724109	0.005302	up
FKBP6	2.419630309	0.005302	up
LOC102174962	1.038712066	0.00531	up
THBS1	3.190485721	0.005317	up
ROBO2	1.899501908	0.005321	up
SSH2	1.132184839	0.005343	up
TFE3	1.502628279	0.005351	up
CD36	1.990067968	0.005431	up
CBLN4	1.670890729	0.005493	up
CBX4	1.2081628	0.005494	up
GDAP1L1	2.835203717	0.005504	up
LOC102175610	1.950361526	0.005545	up
HNF4G	1.622330663	0.005567	up
HYAL1	2.146935677	0.005569	up
FBXL4	1.732398641	0.005577	up
OSCP1	2.317288059	0.005712	up
MYC	3.205171868	0.005731	up

PDCD1LG2	2.891068376	0.005745	up
SLC12A4	1.766861714	0.005767	up
RAB27B	3.551065754	0.005769	up
FSTL3	1.179155852	0.005809	up
MYCL	2.277335245	0.005847	up
ZNF782	1.036837578	0.005883	up
MOCOS	1.612107122	0.0059	up
GAMT	1.826828026	0.005934	up
KANK2	1.170391969	0.005945	up
CCDC146	2.829079019	0.005985	up
MET	1.059645084	0.006022	up
PLEK2	1.96541631	0.006026	up
CD8B	1.757358919	0.006029	up
DLX3	3.205225351	0.006042	up
LOC102172790	1.338837622	0.006061	up
TMOD2	1.775431813	0.006071	up
HEBP1	1.463928533	0.006074	up
PPP2R5A	1.046760914	0.006114	up
CD200	1.610046691	0.006116	up
SH3KBP1	1.961833964	0.006136	up
LOC108633248	2.125631064	0.00615	up
SAXO1	2.350284022	0.006156	up

TNNI3	1.312987917	0.006169	up
ABCA12	1.561789758	0.006187	up
FBXL20	1.023380377	0.006206	up
RILPL1	1.175524909	0.006207	up
LIMA1	1.369762971	0.006207	up
ME2	1.659742456	0.006315	up
USP2	1.217574219	0.00639	up
LOC102170140	2.238053169	0.00644	up
BCL11A	1.530700318	0.006454	up
SRC	1.484802571	0.006491	up
C15H11orf70	2.075101632	0.006539	up
LOC108638269	2.231180038	0.006556	up
CHL1	1.797641119	0.006618	up
VMO1	2.262309237	0.006646	up
CD14	2.4035105	0.006723	up
ZNF174	1.182186267	0.006737	up
MID1IP1	2.078581685	0.006771	up
LOC108634607	1.924737104	0.006773	up
HEATR4	2.37456114	0.006776	up
ZNF462	1.296106591	0.006845	up
WIPF1	2.10692605	0.006898	up
MAPK11	2.772437437	0.006921	up

HESX1	1.359820425	0.006927	up
LOC102190983	1.685407553	0.006928	up
IPO13	1.015323691	0.006977	up
LOC108634738	1.606405754	0.00699	up
SKAP1	1.743637982	0.007072	up
FOXE1	3.019961083	0.007106	up
SLC26A11	1.616112975	0.00711	up
DEGS2	1.156290484	0.007164	up
LOC106501911	1.229211984	0.007165	up
LIN28A	2.098389694	0.007217	up
LGALSL	2.403760805	0.007272	up
CCDC71L	1.228035269	0.007296	up
SLFN11	1.151748578	0.007482	up
SLC39A10	1.029973074	0.007535	up
MGAT4A	1.117046665	0.007581	up
LOC102172703	1.539366474	0.007628	up
LY6E	1.993549916	0.007637	up
VGLL1	2.226687864	0.00766	up
EPB41L3	1.432350711	0.007726	up
ATP10D	1.430071754	0.007771	up
OTUD7B	1.130623063	0.007809	up
YBX2	1.342837534	0.00784	up

LOC102171183	2.163026731	0.00785	up
CALML4	2.829597549	0.007867	up
NLRP9	1.775834415	0.007949	up
FBLIM1	1.079273488	0.00795	up
GPR63	1.106338955	0.007962	up
ARHGAP29	1.120832923	0.007975	up
MARCH3	3.031623738	0.007994	up
LOC102172396	1.097999848	0.008012	up
FSD1	1.934944833	0.008087	up
RCBTB2	1.551746323	0.008114	up
LOC102188783	1.249614941	0.00815	up
NFATC1	1.515769735	0.008202	up
SDC1	1.627076933	0.00832	up
LOC108638392	1.497285364	0.008345	up
QSER1	1.848143419	0.008469	up
PSD3	1.831135619	0.00863	up
HPCA	1.602852195	0.00878	up
APOBR	2.130896832	0.008826	up
RFX7	1.049748055	0.008828	up
FAM168A	1.195362125	0.008871	up
BICC1	1.578575139	0.008872	up
SLC4A8	3.511456288	0.008872	up

C23H6orf132	1.318288593	0.008895	up
SPRYD3	1.252799351	0.008899	up
SIX2	4.664924953	0.008926	up
LHFPL3	1.902780216	0.008959	up
WDR63	1.571651702	0.008976	up
LOC102171073	2.700585347	0.008999	up
LOC108638596	1.317606739	0.00906	up
TMEM256	1.202309751	0.009115	up
RAPGEF1	1.440629578	0.009188	up
UHRF1	1.378109156	0.009228	up
P3H4	1.517647442	0.009271	up
MAJIN	2.246936115	0.009296	up
LAMA4	1.96812841	0.009357	up
FAM81A	1.119275632	0.009567	up
FKBP9	2.347401649	0.009694	up
EIF2AK2	2.149458724	0.009754	up
ARSI	3.128652613	0.009831	up
LOC106502368	1.198549293	0.009858	up
LOC108637951	1.715088735	0.009916	up
LOC102182149	2.199692274	0.009953	up
LOC102175118	3.567420187	0.00996	up
GALNT10	1.155072065	0.009979	up

SLC39A12	2.101212357	0.010072	up
LOC108635335	2.128272237	0.010151	up
FAM110B	1.928759754	0.010167	up
LOC108634236	2.274797943	0.010278	up
RNF44	1.562917488	0.010345	up
LOC106503212	1.87632684	0.010419	up
AMDHD1	2.343330497	0.010442	up
LOC102175147	1.998879455	0.010442	up
LOC106502792	2.864671689	0.010515	up
GPC4	2.133287165	0.010699	up
PCDH9	2.066711023	0.010758	up
LOC108634366	1.794899787	0.010791	up
PARD3B	1.958561887	0.010811	up
CD44	2.213955359	0.010908	up
CDH11	2.028873682	0.010988	up
RBPJL	1.951339961	0.011043	up
RTKN2	2.696052015	0.011056	up
KIAA1147	1.546302055	0.011098	up
PDGFA	1.265869504	0.011137	up
PEX5L	1.979824736	0.011155	up
LOC108633798	1.629994607	0.011176	up
LOC102186538	1.801401586	0.011204	up

EAF2	1.209276804	0.011278	up
SPATA6	2.675622479	0.011323	up
EHF	1.895660873	0.011486	up
EMP3	1.93722717	0.011542	up
CDKL2	1.508862603	0.011607	up
RBL2	1.412262762	0.011621	up
ZNF576	1.024393082	0.011633	up
KCTD11	1.003318554	0.011706	up
LOC108636583	1.043241345	0.011728	up
LOC102182436	1.35420798	0.011757	up
HAS2	2.95891686	0.01184	up
ATL1	1.607976326	0.011924	up
HSPB11	1.262948417	0.011944	up
XG	2.655518573	0.011945	up
TFAP2A	2.599054464	0.012071	up
KIAA1211	1.486652798	0.012164	up
LOC102191280	1.492841683	0.012203	up
CDC42EP1	1.502139348	0.012216	up
CEP126	1.480071568	0.012301	up
LOC102171438	2.294515222	0.012306	up
GNS	1.02963597	0.012312	up
MPP6	1.015576791	0.012374	up

SGMS1	1.292584184	0.012411	up
MIOX	1.264245174	0.012437	up
TMEM216	1.225570139	0.012495	up
PRPH	2.102227561	0.012568	up
LOC102181790	2.070792181	0.012605	up
SLC39A2	1.000801251	0.012608	up
CA5B	2.749269401	0.01273	up
CCDC62	1.53272927	0.012756	up
UGDH	1.712858604	0.012782	up
PDGFB	3.012595732	0.012791	up
SDS	1.951342677	0.012831	up
KLHL5	2.807838058	0.012982	up
POC1B	1.076753269	0.012986	up
ASXL3	2.914035896	0.012986	up
PRKCH	1.691439607	0.013031	up
RBM41	1.014647503	0.013077	up
LOC102178183	2.77002002	0.013258	up
LOC102188506	1.312709802	0.013299	up
TMEM5	1.191123193	0.013313	up
BDNF	1.965571774	0.013343	up
FXYD5	2.282196918	0.013354	up
PODXL	1.361372402	0.013366	up

LOC106503408	1.376860911	0.013499	up
CD58	2.072111044	0.01355	up
CFTR	1.341939188	0.013596	up
LOC102183137	1.81656779	0.013599	up
LOC108634206	1.614448038	0.013689	up
PPP2R3A	1.273302775	0.013845	up
TMEM98	2.974685804	0.013956	up
USP54	1.171484908	0.013975	up
ECSCR	3.425901721	0.013997	up
DSP	1.017737124	0.014234	up
ZNF527	1.848252873	0.014237	up
SGPP1	1.590277048	0.014243	up
LOC102169371	2.101212308	0.014313	up
FN1	1.499737592	0.014456	up
TEAD3	1.271415917	0.014462	up
RRAGD	1.62639098	0.014476	up
NEFM	2.038361063	0.014527	up
MEGF9	1.499192559	0.014559	up
LOC102175129	1.934119575	0.014568	up
PDLIM2	1.282962291	0.014576	up
GAD1	1.795604315	0.014591	up
BHLHB9	1.687267213	0.014599	up

LOC102180704	1.020424511	0.014647	up
AP1S2	1.248379089	0.014668	up
TRIM2	1.015910714	0.014708	up
TSPAN2	2.888594948	0.014766	up
MEP1B	2.124194194	0.014896	up
SEMA3C	1.6596441	0.015018	up
LOC102171908	1.005006052	0.015061	up
HECTD4	1.470401797	0.015066	up
TPD52	1.362270114	0.015107	up
MYL2	2.3416846	0.015111	up
STYXL1	1.737533733	0.015237	up
GLCE	1.128236862	0.015333	up
LOC102172692	2.114069053	0.015393	up
SAT2	1.701263707	0.015426	up
BCL2A1	2.192669391	0.01551	up
INPP4B	1.546569247	0.015515	up
ZNF608	1.15843991	0.015922	up
LOC102188072	1.11745056	0.01593	up
ST6GALNAC2	2.331669437	0.015976	up
DGKI	1.770685824	0.016027	up
PHF19	3.300523235	0.016031	up
WEE2	1.467498915	0.016119	up

UNC5C	1.187082588	0.016233	up
KRT6A	2.624951211	0.016237	up
TRIM54	1.958475224	0.016253	up
EBF1	1.321827358	0.016273	up
FOLR2	2.798451789	0.016369	up
GPX6	1.674209695	0.016611	up
RASA1	1.036674096	0.016621	up
ZNF711	1.481944452	0.016803	up
DEF8	1.521300827	0.01687	up
FAM228A	2.201946336	0.016902	up
CRSP-2	2.715311565	0.016911	up
ELOVL1	1.236502851	0.016945	up
LOC102173612	1.555635095	0.016993	up
KATNAL1	1.501942475	0.017009	up
NOL4L	1.229491166	0.017188	up
PDZD2	1.25774357	0.017308	up
CREB3	1.067455546	0.017433	up
LOC106501861	1.02467706	0.017491	up
CPS1	2.408587469	0.017496	up
LGALS4	1.829184505	0.017538	up
PFKFB3	1.732869327	0.017558	up
XRRA1	1.702290395	0.017578	up

SMAD5	1.041272837	0.017672	up
XKRX	2.291338375	0.017755	up
CD3E	2.475404502	0.017785	up
F5	1.556320854	0.017932	up
LOC108638418	2.56178383	0.018072	up
AIM1L	2.456035268	0.018101	up
LOC108633806	1.37210076	0.018121	up
APOBEC1	1.947359509	0.018138	up
KCNJ14	2.485008137	0.018218	up
CREB5	1.208703751	0.018299	up
CLEC1B	2.858024441	0.018312	up
TSTD1	1.045048176	0.018432	up
IRX4	2.66329164	0.018456	up
SERPINE1	1.4265848	0.018495	up
IRS2	2.031633941	0.018514	up
FRMD7	1.980863258	0.018542	up
MYBPC1	2.211560896	0.018579	up
ELAVL4	2.670243894	0.018746	up
PPP4R4	2.143188459	0.018779	up
LOC106503484	1.071102935	0.01882	up
CLDN23	1.633935808	0.018951	up
PIWIL3	1.787505292	0.01896	up

PRKG1	1.647254106	0.018979	up
PTK7	1.260739347	0.019068	up
EBPL	1.24427052	0.019112	up
ABHD16A	1.380797622	0.019162	up
PBLD	1.056450975	0.019189	up
KANK1	1.028391335	0.019198	up
LOC102170138	1.167585478	0.019269	up
LOC102186952	1.953935894	0.019309	up
BECN2	1.07635865	0.019382	up
MMP1	3.739818903	0.019389	up
TDGF1	1.897489248	0.019405	up
LOC106504020	1.942041075	0.019481	up
RUSC1	2.13093166	0.019734	up
CRABP1	1.587503261	0.019859	up
SEL1L3	1.467639798	0.019882	up
LOC106502835	1.110505492	0.01992	up
CT55	1.040410917	0.019928	up
LOC102187421	1.41105012	0.01996	up
ARHGAP9	2.627142541	0.02002	up
LMX1B	1.727866187	0.02007	up
NUDT6	1.20391698	0.020272	up
TGM3	3.97591558	0.020301	up

MAP2	1.041642637	0.020377	up
LRCH2	2.717483698	0.02061	up
GBGT1	1.504558173	0.020741	up
LOC102188361	2.102629429	0.020768	up
SLA2	2.847632775	0.021003	up
SHPK	2.01757579	0.021108	up
SEMA3E	1.526878404	0.021182	up
AMOTL1	1.335639231	0.021219	up
SYCP2L	1.841215633	0.021234	up
FCGBP	1.705645508	0.021336	up
LOC102173088	2.563166584	0.021354	up
HBEGF	1.025571807	0.021534	up
EPHB3	2.60669221	0.021578	up
COL4A1	1.011911372	0.02158	up
GYG2	1.530716377	0.021624	up
XYLT1	2.365895955	0.021734	up
ETS2	1.305071384	0.021777	up
LOC102177832	1.082643237	0.021787	up
AMER1	1.115470702	0.021872	up
STAMBPL1	3.13753701	0.021971	up
PDE6A	2.036951258	0.021994	up
FBXO43	2.582900311	0.022206	up

LOC102171223	2.052416834	0.02249	up
LOC102170015	3.341911291	0.022549	up
LOC102180474	1.480250371	0.022714	up
NMI	1.405540168	0.022795	up
NANOG	1.470154641	0.022844	up
TRPC3	1.482256522	0.022852	up
CROT	1.039697202	0.022883	up
RPGRIP1	2.282765877	0.023024	up
TRIM36	1.200482085	0.023042	up
CCDC14	1.385016557	0.023201	up
LOC102170645	1.236067149	0.023205	up
ROBO4	3.66985591	0.023223	up
LOC108633879	2.163180151	0.02344	up
LOC108634556	1.13812753	0.023532	up
LONRF3	1.536905074	0.023657	up
CMTM8	1.441270314	0.023713	up
NR4A1	1.66703996	0.023822	up
GPR153	1.586725768	0.023887	up
GNAI2	1.022035109	0.024006	up
HOXC13	1.22793919	0.024073	up
FGF12	2.116508819	0.024087	up
SRGN	1.841762447	0.02412	up

NPM2	1.796554502	0.024608	up
CA8	1.793148187	0.024694	up
CD53	1.281978958	0.024737	up
CCDC28B	1.961748265	0.024848	up
AMPD3	1.843273935	0.024888	up
SMTNL2	1.819598178	0.025074	up
SLC26A3	1.446710868	0.025087	up
FBXO47	1.679480619	0.025087	up
GJB4	1.846867976	0.025121	up
NCOA7	1.442768559	0.025188	up
LOC102172226	1.472695197	0.025283	up
MPP4	3.865692223	0.0253	up
LOC102175184	2.082695718	0.025337	up
MYL4	1.660122166	0.025395	up
LOC106503170	1.068633161	0.025428	up
RARG	2.729414998	0.025462	up
KDELR3	1.380190694	0.025543	up
LTBP1	1.685793973	0.025737	up
LUM	1.534771143	0.025771	up
B3GNT7	3.089477033	0.025804	up
TDRD1	1.053789224	0.026004	up
CASR	1.975595986	0.026196	up

ZSWIM6	1.559684523	0.026224	up
COL5A2	1.011728674	0.02624	up
PARP4	1.751469625	0.026272	up
ZNFX1	1.076563276	0.02629	up
PEAR1	2.465606368	0.026291	up
GABRA3	2.310195158	0.026327	up
LOC108636004	1.46265985	0.026346	up
LOC108636015	1.785230281	0.026569	up
SIX4	1.426163257	0.026642	up
GATSL2	1.116065229	0.026673	up
LAPTM5	2.567709783	0.026909	up
DEXI	1.99721027	0.026997	up
FAM71F2	2.057944073	0.02702	up
SHB	2.34292551	0.027105	up
LOC108636604	1.786632365	0.027111	up
MVP	2.327254082	0.027137	up
KHDC3L	1.566442426	0.027163	up
LOC102187883	1.661887907	0.027265	up
HES6	1.846117191	0.027316	up
PPP1R1A	1.161323669	0.027434	up
GABRG2	3.070038109	0.027626	up
TMEM171	1.116766821	0.027649	up

LOC102168905	2.556440214	0.027682	up
ETV4	1.974485811	0.027706	up
PITX2	1.792745025	0.027713	up
SLC35F6	1.26184764	0.027762	up
DCLK1	1.835552695	0.027815	up
BFSP1	1.977371848	0.027852	up
PLCB3	1.44130573	0.027882	up
LOC102186291	1.131120113	0.027886	up
MUM1L1	1.930928726	0.027968	up
WDR11	1.015445247	0.028209	up
GTPBP2	1.169159306	0.028422	up
LOC106502310	1.788345859	0.028436	up
LOC108636719	1.515450343	0.028473	up
SMARCA1	1.834683894	0.028559	up
GSN	1.318086168	0.028569	up
RNF39	1.437334229	0.028817	up
ANKRD13B	2.873581301	0.028977	up
NAAA	1.45800133	0.02903	up
PFN4	1.708299551	0.029226	up
MDK	2.207748023	0.029357	up
PBX1	2.219158848	0.029645	up
LOC102171684	1.558765753	0.029862	up

ELMO1	3.810923522	0.029899	up
PLCH1	1.644640729	0.02993	up
SUSD1	1.22221826	0.029991	up
ST14	1.218858178	0.030074	up
CNR2	1.556088536	0.030196	up
LOC102180297	1.292405377	0.030264	up
LPCAT4	1.58238435	0.030267	up
CAMTA2	1.380053008	0.030305	up
KCNA5	1.729375709	0.030413	up
PI4K2B	1.736975035	0.030511	up
VIPR1	1.959212277	0.030552	up
LOC108633292	1.842969813	0.030649	up
PI3	1.941508679	0.030702	up
PLCB1	1.808395489	0.03074	up
FSTL1	3.088931204	0.03079	up
SH3GLB2	1.515793676	0.03101	up
CPNE4	1.777022678	0.03102	up
ATP1B4	2.361111274	0.031065	up
DNALI1	1.734695781	0.031085	up
RNF125	1.910249986	0.031414	up
FERMT1	1.600158168	0.031478	up
PRSS12	1.392657734	0.031572	up

AVEN	1.239741336	0.031752	up
FAM227B	2.952601468	0.031752	up
LOC102188567	1.878747907	0.031789	up
CCDC155	1.734150798	0.032648	up
TNK1	1.484385273	0.032665	up
CHMP4C	1.774641726	0.032672	up
LOC108635249	2.141290747	0.032771	up
TP63	1.105750863	0.033024	up
LOC102171395	1.70276842	0.033093	up
LZTS3	1.421286071	0.033282	up
SBSPON	2.002849497	0.033519	up
ANGPT1	1.694220677	0.03356	up
DNAJB4	1.243052195	0.033943	up
ZFHX4	1.302533494	0.033948	up
LIMK1	1.451240156	0.034271	up
LOC108637515	1.116332748	0.034477	up
LOC102183064	1.771817072	0.034658	up
CCDC85B	1.342772516	0.03466	up
LOC102177207	1.46938475	0.034712	up
DOCK11	1.867955141	0.034737	up
NBEA	1.929485828	0.034871	up
PGPEP1L	1.235515801	0.035033	up

LOC108637626	1.083396378	0.035419	up
TGFB2	1.076723194	0.035422	up
IQCK	1.570613573	0.035524	up
MESDC1	1.743962377	0.035559	up
TMEM67	1.246117051	0.035871	up
LOC106503430	2.113232649	0.035898	up
DNAH8	1.133916974	0.03595	up
LOC102172423	1.70720749	0.036186	up
TMEM52B	2.661767677	0.03642	up
LOC108636993	2.292581405	0.036475	up
DGUOK	1.060303362	0.036693	up
SLC20A2	1.050381609	0.036762	up
PBXIP1	1.66465034	0.036808	up
LOC102182748	1.476570273	0.036924	up
FAM25A	1.760683592	0.037201	up
CAMK2N1	1.724852521	0.037284	up
RBFOX1	2.687625998	0.037576	up
NR1H4	2.910394223	0.037766	up
LRRC58	1.399444677	0.037864	up
ZNF169	2.279182744	0.037983	up
RNF213	2.187132257	0.037984	up
MPRIP	1.303977742	0.03827	up

ANXA3	1.044329344	0.038305	up
LOC108637420	1.777312326	0.038945	up
ROR2	2.212611778	0.039117	up
TCF7L1	1.350400883	0.039329	up
ADCK2	1.09892067	0.039452	up
SH3BGR	1.439227651	0.039557	up
LEUTX	1.749274136	0.039623	up
LOC102180551	1.435158224	0.039713	up
MFSD12	1.285115453	0.039813	up
EHHADH	1.513434019	0.039997	up
SLC12A5	2.156822557	0.040103	up
CACTIN	1.124898545	0.040156	up
NFE2L3	1.438752563	0.040183	up
FGF2	1.895299584	0.040184	up
LOC102177047	1.592786724	0.040268	up
GGA1	1.212879071	0.040508	up
GPR137B	1.495290405	0.040527	up
ADAMTS3	1.191803653	0.040716	up
IKZF3	1.670704083	0.0409	up
ITGA9	2.051019852	0.040939	up
LTBP2	3.349636667	0.040959	up
GATA2	1.199585088	0.040963	up

NCKAP5L	2.350479022	0.04118	up
CUX2	2.137203896	0.041324	up
UST	1.318823661	0.041342	up
FBXL7	2.196116998	0.041761	up
FRZB	1.624485005	0.041787	up
LOC102191067	1.247688334	0.042254	up
PHYHIPL	1.571293275	0.042288	up
LOC102177456	1.262058814	0.042328	up
MRC1	1.75875291	0.043028	up
NAV3	1.294294019	0.043127	up
PLEKHA6	1.110207707	0.043175	up
GREB1	1.348488495	0.043247	up
LOC108636071	2.329022553	0.043336	up
ABCC2	1.240833692	0.043338	up
LOC102191534	1.819456419	0.043576	up
ST3GAL2	1.760879413	0.043577	up
ARHGAP32	1.254851204	0.043622	up
HECA	1.288274506	0.043864	up
LOC102178000	1.378420542	0.043887	up
ZFP90	1.399694809	0.044034	up
GJA5	1.179261428	0.04411	up
JAK3	1.250113123	0.044341	up

MSH5	1.008458523	0.044414	up
TBC1D13	1.222559574	0.044477	up
SLC25A12	2.123699659	0.044534	up
GPNMB	1.731900313	0.044715	up
GREB1L	1.670535432	0.044821	up
LOC102168260	2.568390267	0.04491	up
TTC23	1.194322931	0.04496	up
MYL9	1.424274205	0.044995	up
SSBP2	1.145483233	0.045401	up
UPK1A	2.694299694	0.045586	up
EVC2	2.35449688	0.04588	up
SCN3B	2.19016635	0.046006	up
EPPK1	1.278719618	0.046056	up
LOC108634031	1.518231041	0.046212	up
LOC102176586	1.734222085	0.04623	up
LOC102172074	2.242659532	0.046261	up
MICALL2	1.191590578	0.046412	up
LOC102175132	1.263278494	0.04656	up
LOC102175171	1.058330757	0.046705	up
ZP4	1.156350624	0.04709	up
FBLN1	1.336861468	0.047136	up
TMEM116	1.024340762	0.047208	up

LOC102169313	1.134020257	0.047472	up
NDRG2	2.674908464	0.047534	up
REPIN1	1.355596702	0.047734	up
PLCG2	1.198915186	0.047817	up
FGL1	2.60669388	0.047971	up
LOC102179926	2.252180839	0.048101	up
LOC102188715	1.622029356	0.048219	up
AGPAT3	1.354739851	0.048273	up
LOC108635581	1.174163588	0.048291	up
CDON	1.370437469	0.048414	up
MAPK12	1.44042592	0.048505	up
IDH2	1.775604711	0.048644	up
LOC102172133	1.698671481	0.048742	up
LINGO2	1.595310721	0.048894	up
LOC108638246	1.665764517	0.048952	up
CRY1	1.084398296	0.049318	up
SLAMF7	1.76417315	0.049457	up
ZSWIM4	1.860825615	0.049813	up
JAZF1	1.419702507	0.049876	up
LOC102183395	1.058045678	0.049885	up
LOC102182159	1.775995716	0.049933	up
LOXL2	2.356851682	0.049946	up

PHGDH	-3.383879233	3.82E-23	down
BLVRB	-1.398365842	2.9E-11	down
LOC108634563	-2.524790591	3.62E-11	down
BEX4	-2.876420365	5.11E-11	down
HAX1	-1.460084496	1.36E-10	down
LOC102184663	-6.130104257	2.21E-10	down
CTH	-3.172049114	9.41E-10	down
KRTCAP3	-1.338376735	2.66E-09	down
CHAC1	-1.809523082	2.26E-08	down
SRSF7	-1.06690667	3E-08	down
NRBP1	-1.29878152	3.47E-08	down
CPM	-1.266263309	6.34E-08	down
TNC	-2.571399911	1.97E-07	down
BEX2	-1.871975787	2.11E-07	down
LOC102168687	-3.255996947	2.85E-07	down
ACOX2	-1.031273815	4.75E-07	down
STC2	-4.62878897	5.06E-07	down
SARS	-1.104122996	5.33E-07	down
HMBS	-1.172328937	1.14E-06	down
UBE2S	-1.031856358	1.42E-06	down
MAP28	-5.516651457	3.04E-06	down
CEBPG	-1.080694629	3.09E-06	down

SNX7	-1.060578588	3.26E-06	down
C1QBP	-1.004577936	3.36E-06	down
TNFSF9	-1.145465611	3.5E-06	down
RENBP	-1.185220506	4.34E-06	down
LOC108638067	-1.161776359	4.78E-06	down
METTL21A	-1.302614439	8.03E-06	down
KCNE3	-1.333302317	8.7E-06	down
SUMO3	-1.268353321	9.12E-06	down
UBXN2A	-1.238757492	1.34E-05	down
PKIA	-1.147552789	1.34E-05	down
LOC108633351	-1.320761173	1.39E-05	down
JADE1	-2.785024627	1.4E-05	down
MRPS34	-1.095325071	1.48E-05	down
SLC26A4	-1.281213572	1.73E-05	down
GJA1	-1.001661437	1.79E-05	down
MIS18A	-1.001916874	2.4E-05	down
LOC108635364	-12.01406814	2.56E-05	down
LOC102186527	-1.416195072	2.71E-05	down
SYNE1	-1.101445121	2.71E-05	down
PSAT1	-2.995096495	3.77E-05	down
LOC102189740	-2.316976904	4.8E-05	down
SLC6A9	-3.184594538	5.24E-05	down

TLCD1	-1.10521246	5.4E-05	down
RAB11FIP1	-1.69883726	7.05E-05	down
EIF4EBP1	-1.51675095	7.52E-05	down
PDCD2L	-1.239869788	8.03E-05	down
HOXA11	-1.852409998	9.1E-05	down
UFL1	-1.001293988	9.59E-05	down
RHBDL2	-1.371645309	0.000101	down
MAP3K4	-1.278809768	0.000105	down
L3MBTL3	-1.244600685	0.000114	down
ENPP5	-1.476825089	0.000118	down
ADAMTSL3	-2.742398674	0.000137	down
ACSM5	-1.917654139	0.000146	down
NIT2	-1.126003262	0.000154	down
LOC102173852	-2.024685476	0.000159	down
LOC108636601	-1.007766196	0.000164	down
RASA3	-2.693561908	0.000184	down
PFKFB4	-1.024089191	0.000188	down
ACD	-1.004876064	0.000194	down
CCDC107	-1.546818318	0.000231	down
IPO4	-1.045459287	0.00025	down
SETD6	-1.534962083	0.00026	down
SH2B3	-2.173952848	0.000273	down

STYK1	-1.283893242	0.000279	down
JMJD8	-1.052075408	0.00029	down
DHX30	-1.222140211	0.000291	down
HDHD3	-1.069358702	0.000298	down
GJB7	-2.162243609	0.000348	down
GARS	-1.160431871	0.000404	down
MTG1	-1.051440313	0.000431	down
NT5C3B	-1.059617745	0.000471	down
LOC102184297	-1.919468704	0.000474	down
METTL25	-1.477853318	0.000524	down
LOC102188417	-1.016067222	0.000537	down
LOC102178027	-1.106780497	0.000758	down
LOC108634238	-1.564809426	0.000784	down
PIK3R1	-1.076194706	0.000804	down
DHRS7	-1.044486946	0.001079	down
LOC102174202	-1.025261127	0.001102	down
GNPNAT1	-1.10084508	0.0012	down
ASNS	-1.028807339	0.001212	down
LOC108634314	-3.242940049	0.001237	down
LOC108634430	-2.02533861	0.00125	down
TRMU	-1.107202037	0.001253	down
RUFY1	-1.012827492	0.001258	down

STC1	-2.180864988	0.001328	down
TFAP2D	-1.081058119	0.001418	down
RELB	-2.730670622	0.001442	down
LOC102180655	-1.531963551	0.001497	down
LOC102176755	-1.14313413	0.001551	down
CBS	-1.413840293	0.001658	down
FAM89A	-1.201849712	0.001665	down
LOC108634559	-1.167872666	0.002063	down
ANKZF1	-1.080578472	0.002102	down
LOC102181343	-1.276071998	0.002211	down
C11H9orf142	-1.266800942	0.002376	down
LOC108634776	-1.02151713	0.002376	down
NUPR1	-1.690550822	0.002517	down
LOC102172960	-1.606583925	0.002559	down
RPL22L1	-1.12386107	0.002595	down
LOC102184275	-1.116604522	0.002722	down
SLC7A3	-2.612428912	0.002731	down
LOC102180290	-1.014959282	0.00291	down
LOC108636867	-2.338955642	0.003023	down
LOC106501755	-1.547775535	0.003082	down
KCNS3	-1.602800298	0.003161	down
KIT	-1.371496767	0.003173	down

LOC108635823	-1.824905975	0.003194	down
PSMA8	-1.1263437	0.003376	down
CAMKK2	-1.305038597	0.003961	down
MBTPS2	-1.328560691	0.004495	down
LOC108635892	-1.837304599	0.004905	down
LOC108633164	-1.50083274	0.006135	down
NFE2	-1.010104162	0.00615	down
SLC43A1	-1.44937902	0.006251	down
C21H14orf28	-1.280245959	0.006472	down
LOC102173837	-1.511558928	0.006607	down
ADAMTS17	-1.054368017	0.007249	down
SOGA1	-1.057804385	0.007582	down
CTNND2	-1.195904226	0.007848	down
LOC106503979	-1.086379865	0.008012	down
SLC7A11	-2.576165956	0.008359	down
GKN2	-1.930385324	0.008371	down
ZIC2	-1.286618153	0.008515	down
LOC102175154	-1.276739732	0.008979	down
AP1S3	-1.082432473	0.009423	down
LOC102171231	-1.316237527	0.010437	down
LOC102180443	-1.63333463	0.010702	down
LOC102169678	-1.586530647	0.010944	down

LIMD2	-1.192896907	0.011499	down
SPEF2	-1.310282697	0.011781	down
CLDN8	-1.171492383	0.011973	down
LOC102187765	-1.390607432	0.013155	down
DUSP23	-1.037254767	0.013654	down
LOC108633161	-1.029446741	0.014531	down
FGFR4	-1.034052797	0.014589	down
LOC102169326	-2.017247544	0.014927	down
MEST	-1.789559967	0.01542	down
STK16	-1.092234491	0.015583	down
AHSG	-1.157294106	0.016054	down
LOC102176915	-2.232701654	0.016735	down
JPH3	-1.556802397	0.017144	down
LOC108636650	-1.736843256	0.017944	down
ZXDC	-2.643463588	0.018005	down
LOC102191230	-1.012362613	0.019	down
LOC102182160	-1.220639234	0.020822	down
ANKRD27	-1.084933659	0.021113	down
PLA2G5	-1.20389911	0.021132	down
SFTPB	-1.053199853	0.0218	down
SLC7A1	-2.332282998	0.022426	down
FSIP2	-2.333579066	0.022562	down

PCBP3	-1.351708285	0.022698	down
LOC108633305	-1.02228561	0.023372	down
ZNF311	-1.544165901	0.023556	down
RASD1	-2.477598774	0.023584	down
LOC108638088	-1.653973477	0.023744	down
EEFSEC	-1.045851797	0.023814	down
EXPH5	-1.030709676	0.024627	down
LOC102181641	-1.026433806	0.025034	down
ZCCHC2	-1.38639535	0.02541	down
LOC108636220	-1.035929496	0.025945	down
CADM4	-1.886600417	0.025995	down
LOC102183122	-1.201838859	0.02632	down
NR2E1	-1.73231935	0.026475	down
LOC108634558	-1.186495475	0.026857	down
LOC108633470	-1.727233675	0.0273	down
LOC102169851	-1.341543866	0.028209	down
SAP30	-1.009704137	0.028583	down
HSD3B7	-1.844996416	0.029109	down
KEL	-1.250869979	0.029556	down
UGGT2	-1.001904644	0.029713	down
LOC102168304	-1.66119662	0.029951	down
LOC108636857	-1.255438905	0.030219	down

ETS1	-1.028217277	0.030265	down
LOC102176695	-1.652241601	0.030279	down
SPESP1	-1.054499424	0.030293	down
DOCK2	-1.061959668	0.030658	down
SHROOM1	-1.192233443	0.032998	down
KCNA3	-1.407643612	0.033622	down
LOC102168336	-1.073371568	0.033815	down
PRDM6	-1.212605088	0.035536	down
PYCR1	-1.672251305	0.038309	down
HPSE	-2.081277404	0.039173	down
GPR171	-1.1767416	0.041143	down
TPGS2	-1.448485875	0.041253	down
MUTYH	-1.029504747	0.047205	down
LOC102184252	-1.87118767	0.047268	down
LOC108635390	-1.490072573	0.04761	down
RARRES1	-1.36654545	0.047637	down
C5H12orf74	-1.685678714	0.048634	down
LOC106502679	-1.34500123	0.049282	down

| Log_2(Fold change) | ≥ 1 and adjusted FDR < 0.05 was the cut-off criteria for DEGs.

Table S5: The 20 key GO terms of DEGs in H8C and L8C groups.

GO_ID	GO-function	S gene number	B gene number	P value
GO:0006955	Biological-process	24.00	242.00	1.04E-11
GO:0008009	Molecular-function	11.00	43.00	1.60E-10
GO:0030593	Biological-process	11.00	49.00	7.38E-10
GO:0000788	Cellular-component	10.00	44.00	3.90E-09
GO:0006954	Biological-process	18.00	210.00	4.11E-08
GO:0071222	Biological-process	10.00	57.00	5.53E-08
GO:0045236	molecular-function	5.00	8.00	9.08E-08
GO:0070374	Biological-process	14.00	132.00	9.66E-08
GO:0050829	Biological-process	7.00	24.00	1.38E-07
GO:0005615	Cellular-component	41.00	950.00	1.40E-07
GO:0006334	Biological-process	12.00	101.00	2.30E-07
GO:0019864	Molecular-function	4.00	5.00	4.77E-07
GO:0038094	Biological-process	4.00	5.00	4.77E-07
GO:0070098	Biological-process	9.00	56.00	5.65E-07
GO:0009897	Cellular-component	14.00	155.00	7.07E-07
GO:0042612	Cellular-component	6.00	24.00	3.04E-06
GO:0019885	Biological-process	4.00	7.00	3.25E-06
GO:0045087	Biological-process	16.00	250.00	1.08E-05
GO:1900121	Biological-process	4.00	9.00	1.14E-05
GO:0042742	Biological-process	9.00	84.00	1.77E-05

S gene number: the number of genes annotated with significant differences for a particular GO.

B gene number: annotated as the number of genes in a particular GO.

Supplementary Table S6: The 20 key KEGG pathways of DEGs in H8C and L8C groups

Pathway-id	S gene number	B gene number	P value
ko04145	26	346	1.4821E-09
ko05323	15	109	1.5572E-09
ko05322	18	219	1.4209E-07
ko05150	13	119	3.1613E-07
ko04062	17	217	6.2767E-07
ko04612	12	112	1.1165E-06
ko05203	20	328	3.3475E-06
ko05140	10	90	6.3764E-06
ko04672	9	72	7.0197E-06
ko04060	21	382	9.4967E-06
ko05168	19	344	2.3857E-05
ko05332	8	70	4.4329E-05
ko05152	16	280	7.0701E-05
ko04640	11	148	9.9612E-05
ko04940	8	82	0.00013816
ko05330	7	63	0.00015998
ko04142	14	246	0.00020832
ko04620	11	164	0.00024658
ko05133	9	113	0.00025414
ko05320	7	77	0.00055784

S gene number: the number of genes annotated with significant differences for a particular GO.

B gene number: annotated as the number of genes in a particular GO.

Supplementary Table S7: The 20 key GO terms of DEGs in HM and LM groups

GO_ID	GO-function	S gene number	B gene number	P value
GO:0045569	Molecular-function	8.00	12.00	4.04E-07
GO:0070062	Cellular-component	226.00	2191.00	5.93E-07
GO:0004859	Molecular-function	6.00	7.00	1.23E-06
GO:0005737	Cellular-component	326.00	3402.00	1.70E-06
GO:0042127	Biological-process	28.00	158.00	2.06E-05
GO:0008625	Biological-process	12.00	41.00	3.45E-05
GO:0007613	Biological-process	12.00	41.00	3.45E-05
GO:0016477	Biological-process	23.00	125.00	5.99E-05
GO:0045060	Biological-process	6.00	11.00	6.24E-05
GO:0007612	Biological-process	11.00	38.00	8.16E-05
GO:0043086	Biological-process	12.00	45.00	9.46E-05
GO:0001968	Molecular-function	6.00	12.00	1.17E-04
GO:0045995	Biological-process	5.00	9.00	2.42E-04
GO:2000178	Biological-process	5.00	9.00	2.42E-04
GO:0005911	Cellular-component	20.00	112.00	2.68E-04
GO:0001570	Biological-process	11.00	43.00	2.74E-04
GO:0001077	NA	28.00	183.00	2.88E-04
GO:0000788	Cellular-component	11.00	44.00	3.41E-04
GO:0016324	Cellular-component	27.00	178.00	4.21E-04
GO:0001765	Biological-process	3.00	3.00	4.35E-04

S gene number: the number of genes annotated with significant differences for a particular GO.

B gene number: annotated as the number of genes in a particular GO.

Supplementary Table S8: The 20 key KEGG pathways of DEGs in HM and LM groups

Pathway-id	S gene number	B gene number	P value
ko05034	40	245	5.96E-06
ko05205	47	308	6.34E-06
ko04390	36	227	3.27E-05
ko05206	43	309	1.41E-04
ko04261	38	263	1.55E-04
ko04151	58	458	1.56E-04
ko05203	44	328	2.72E-04
ko05412	19	111	8.89E-04
ko04918	17	95	9.77E-04
ko04728	25	165	9.87E-04
ko05200	63	545	9.89E-04
ko04210	29	205	1.25E-03
ko05219	12	57	1.25E-03
ko05162	26	177	1.26E-03
ko04115	23	150	1.31E-03
ko04152	23	152	1.57E-03
ko05144	16	91	1.65E-03
ko04512	31	231	2.04E-03
ko04520	25	177	2.67E-03
ko04510	71	658	3.03E-03

S gene number: the number of genes annotated with significant differences for a particular GO.

B gene number: annotated as the number of genes in a particular GO.

Supplementary Table S9: The FPKM with 66 DEGs between H8C and L8C groups and HM and LM groups.

**Gene-name**	**H8C1**	**H8C2**	**H8C3**	**L8C1**	**L8C2**	**L8C3**	**HM1**	**HM2**	**HM3**	**LM1**	**LM2**	**LM3**
AMMECR1	27.39057	6.222587	129.02814	3.7913	4.35331	10.66623	4.555895	3.914973	2.827014	0.983558	1.611915	1.216913
B2M	186.0369	7.222587	25.910767	3.804166	0.151064	2.315674	49.69528	97.52278	44.93232	21.14271	25.76525	33.34206
BCL2A1	2.694022	8.222587	11.068684	0.0001	0.049012	0.336618	0.761792	0.640381	1.352822	0.271393	0.048117	0.283135
BHLHE40	1.734495	9.222587	9.449632	0.074526	0.068923	0.446049	1.924238	1.29342	1.253471	0.552131	0.288603	0.412363
BLOC1S2	1.66217	10.22259	9.9013288	0.089233	0.133071	0.577576	241.8634	300.2991	317.3583	102.9967	102.5585	84.02531
C5H12orf75	567.6102	11.22259	48.507801	74.26254	102.5325	38.65886	2.335356	0.349792	0.66073	0.0001	0.175191	0.0001
CASR	3.754873	12.22259	10.122665	0.0001	0.443659	0.209108	0.167508	0.215822	0.32783	0.099492	0.027217	0.054114
CD14	5.078474	13.22259	36.780599	0.0001	0.0001	0.0001	0.546163	0.399796	0.445106	0.135894	0.021365	0.105658
CD36	201.8343	14.22259	345.90912	0.0001	0.716143	1.977396	0.678391	0.769513	0.740801	0.331087	0.072603	0.147266
CD55	1.404492	15.22259	10.164432	0.033846	0.145679	0.138741	5.853389	11.03656	9.463975	2.494759	3.793453	4.659787
CLEC4E	18.19285	16.22259	171.29163	0.0001	0.168596	0.255635	6.895	19.23368	31.13458	1.388536	1.328791	1.499935
CTGF	185.7647	17.22259	386.15921	14.61954	32.99216	8.434348	12.84512	28.79476	13.68531	5.917621	5.288311	9.308692
CTSL	2.196288	18.22259	10.489251	0.0001	0.0001	0.0001	28.88228	89.65329	122.9164	31.30316	25.2115	27.13065
CUX2	1.090596	19.22259	28.09486	0.109886	0.37334	0.913602	0.33357	0.287944	0.238349	0.0001	0.142528	0.052836
EIF2AK2	1.887915	20.22259	4.408348	0.112838	0.152628	0.320952	1.138743	0.484165	0.797482	0.051354	0.116855	0.377339
ENPP1	1.018366	21.22259	10.777434	0.688247	0.867528	0.612913	8.597221	1.679229	1.670047	1.281098	1.037222	0.756146
ENPP5	5.604435	22.22259	18.263386	0.080073	0.101612	0.0001	1.538425	1.45818	1.663975	6.43824	2.289118	4.244692
FOLR2	82.01662	23.22259	1709.2194	0.721165	1.331336	0.831897	0.197158	1.322003	2.013807	0.317811	0.189923	0.0001
GJA1	0.691137	24.22259	0.418529	0.0001	0.0001	0.0001	39.21246	26.47822	42.80813	58.54853	80.22705	78.47209
HEBP1	6.850292	25.22259	71.80304	0.928755	1.037781	0.416711	35.31809	9.331114	8.990926	8.01291	7.010006	4.421881

HNMT	8.292335	26.22259	9.290145	0.0001	0.074711	0.0001	0.876505	1.054253	1.358708	0.0001	0.037234	0.0001
IL2RG	3.151015	27.22259	16.708719	0.0001	0.095645	0.0001	0.659268	0.899428	0.43485	0.06137	0.0001	0.0001
ITGA4	0.0001	28.22259	0.0001	0.054899	0.515909	0.597398	0.088364	0.176794	0.418227	0.031155	0.038284	0.021913
KCNA3	41.5283	29.22259	9.360742	1.184923	0.247188	0.0001	0.046924	0.135532	0.074956	0.219678	0.277842	0.185403
KIF21A	5.787611	30.22259	23.632462	0.140078	0.141496	1.123991	7.71422	2.28616	2.025523	2.074042	1.121912	0.668388
LAPTM5	16.92649	31.22259	222.9151	3.76662	5.333091	14.68054	0.294246	1.564226	0.494139	0.267587	0.129132	0.0001
LITAF	0.359413	32.22259	52.171593	1.170608	0.191697	0.598646	82.69804	53.80144	62.94333	36.24371	30.97984	28.87971
LOC102168687	79.55785	33.22259	140.00349	4.385259	14.52698	3.651889	0.161714	1.092758	0.180108	3.501406	4.195933	6.007613
LOC102169861	45.57385	34.22259	21.96333	9.865852	11.05198	8.344605	2.049779	6.302172	5.393992	0.171973	0.314068	0.149799
LOC102172037	3.471279	35.22259	7.9398324	0.0001	0.0001	0.0001	1.021336	15.22128	29.11905	0.651678	3.51971	3.003891
LOC102173131	21.37426	36.22259	342.67334	0.072018	0.0001	0.0001	0.102969	2.022434	2.80134	0.0001	0.0001	0.0001
LOC102179924	35.70918	37.22259	74.143417	0.0001	2.361535	1.090304	6.153804	15.27229	14.39432	1.455688	0.535262	1.239518
LOC102180110	1.27357	38.22259	60.776855	0.0001	0.0001	0.0001	14.21014	18.13884	17.71437	2.147197	2.060382	2.621759
LOC102180655	0.0001	39.22259	0.0001	1.318435	1.443946	0.0001	13.65724	3.949799	2.247918	23.02886	17.10265	17.2849
LOC102184252	2.715662	40.22259	8.2435184	0.0001	0.2481	0.333556	0.544969	0.0001	0.091431	0.649825	0.778646	0.90006
LOC102186003	5.80584	41.22259	0.0001	0.0001	0.0001	0.0001	16.41028	6.760868	41.40779	1.419101	0.894001	1.516941
LOC102186527	1.480186	42.22259	228.2197	0.0001	0.083715	0.0001	69.94218	36.74974	24.20255	70.79139	166.5827	111.958
LOC108633237	8.191915	43.22259	15.590065	0.586017	0.411788	1.007041	3.024565	2.635057	1.513183	0.409686	0.371666	0.0001
LOC108634238	1082.38	44.22259	1651.2845	95.53519	100.2785	0.141342	6.039352	2.6797	1.977703	13.42833	10.28124	7.935539
LOC108634366	77.5345	45.22259	522.72296	25.82672	31.54573	56.95118	13.94547	66.1284	26.26351	2.371747	5.301616	22.97218
LOC108634440	1.452218	46.22259	6.247712	0.0001	0.038668	0.0001	11.12079	5.22667	21.86762	1.049661	0.723168	0.916738
LOC108634443	2.19534	47.22259	2.207733	0.0001	0.0001	0.0001	15.85347	6.286497	20.33045	0.804849	0.315826	0.490234

LOC108634456	14.49129	48.22259	186.51691	0.0001	0.260182	0.0001	12.22944	13.49341	11.24465	1.252774	1.782521	2.151198
LOC108635630	0.183626	49.22259	0.295746	0.0001	0.0001	0.0001	0.292774	9.238443	3.361735	0.166684	0.0001	0.860696
LOC108637665	14.0396	50.22259	10.393849	0.148777	0.167851	0.108309	8.828726	6.027174	0.177193	0.183923	0.213734	0.272965
MRC1	3.820436	51.22259	12.62632	0.094709	0.612592	0.179773	1.020659	0.187807	0.215595	0.172752	0.152783	0.09528
NUPR1	8.502779	52.22259	30.035353	2.286271	0.911204	2.510773	13.52114	2.167522	1.848948	28.79492	14.94466	12.86828
OLR1	4.692432	53.22259	23.923502	0.048929	0.283015	0.628902	1.946011	4.044212	5.203015	0.296712	0.0001	0.326683
PCBP4	2.489219	54.22259	24.119204	0.0001	0.078836	0.309999	5.462187	5.151195	4.824081	0.736598	1.71694	0.123328
PRKAR2B	1.143085	55.22259	8.6492957	0.148164	0.232555	0.181782	1.154415	1.288034	1.359422	0.368263	0.324862	0.416869
PTN	61.01282	56.22259	62.889256	2.016126	1.793969	0.128119	0.533943	1.799424	1.488105	0.139549	0.407385	0.056481
RAB31	6743.51	57.22259	38530.184	1571.48	1709.929	4321.991	0.589364	2.705382	1.659507	0.053747	0.091935	0.041576
RNF125	139.89	58.22259	1336.4608	0.522057	2.349111	24.31353	0.109606	0.512378	0.457087	0.134794	0.033695	0.118594
RNF213	6.656619	59.22259	30.797992	0.262989	0.422352	0.0001	0.123874	0.080448	0.069414	0.023395	0.036614	0.0001
SAT1	1.162439	60.22259	6.2554843	0.010252	0.259114	0.100833	452.906	1159.631	1216.757	625.6074	196.2998	214.448
SERTAD4	0.722779	61.22259	3.023445	0.07	0.0001	0.052085	1.421085	1.496257	1.345582	0.437174	0.295456	0.357272
SGPP1	51.54351	62.22259	66.747734	0.0001	0.549445	0.289632	7.39086	1.216916	1.662047	1.332679	1.375473	0.702535
SH3BGRL3	0.0001	63.22259	0.042549	1.401218	1.610493	0.557276	680.1162	1584.897	1701.051	546.4583	278.8654	396.3576
SPATS2L	24.24042	64.22259	227.25103	0.0001	1.420763	5.527192	3.803976	7.543389	11.58096	0.94629	2.823853	1.44885
SRGN	1.192005	65.22259	16.829287	0.0001	0.0001	0.024306	1.271479	1.972086	0.988832	0.596459	0.291714	0.292584
TCEAL9	3.878918	66.22259	10.442064	0.0001	0.06963	0.531785	6.231921	4.631107	4.704242	1.903181	1.543405	1.553749
TPD52	2.980817	67.22259	19.221964	0.085478	0.845686	0.281057	30.61637	7.478103	4.5903	5.031817	6.3935	5.177769
TRIM38	1.196004	68.22259	9.356319	0.288968	0.264361	0.27623	1.269416	1.394806	2.507759	0.578092	0.105117	0.15709
VAMP5	1.076602	69.22259	16.073542	0.0001	0.085095	0.0001	23.89006	23.64727	24.51303	12.82163	3.536117	2.921875

WIPF1	36.2283	70.22259	133.52277	0.194084	5.210567	0.515665	7.710636	1.957819	0.856599	0.806948	1.449212	0.187138
ZNF711	8.722135	71.22259	73.248802	0.0001	0.851264	0.612764	0.693177	1.516496	0.590077	0.320347	0.186791	0.49519

Supplementary Table S10: The 66 DEGs KEGG enrichment analysis

Pathway-id	Pathway-name	Genes	S gene number	B gene number	P value
5.20E+03	Viral carcinogenesis	BLOC1S2FOLR2, PCBP4, ZNF711, NUPR1, RNF213	8.00	8.00	0.11767133
		, TCEAL9, LOC108635630			
4.51E+03	Focal adhesion	CD55, ENPP5, PCBP4, ENPP1	4.00	4.00	0.35854906
5.03E+03	Alcoholism	FOLR2, NUPR1, ZNF711, LOC108635630	4.00	4.00	0.35854906
5.20E+03	Transcriptional misregulation in cancer	RNF213, PTN, BLOC1S2, PCBP4	4.00	4.00	0.35854906
4.39E+03	Hippo signaling pathway	AMMECR1, CD55, CTGF, PCBP4	4.00	4.00	0.35854906
4.12E+03	p53 signaling pathway	LOC102173131, BLOC1S2, PCBP4, RNF213	4.00	4.00	0.35854906
5.32E+03	Systemic lupus erythematosus	NUPR1, FOLR2, LOC108635630, ZNF711	4.00	4.00	0.35854906
4.06E+03	Cytokine-cytokine receptor interaction	LOC108634456, SGPP1, AMMECR1, LOC108634440	4.00	4.00	0.35854906
4.07E+03	HIF-1 signaling pathway	LOC102179924, BLOC1S2, MRC1	3.00	3.00	0.46694761
4.07E+03	FoxO signaling pathway	RNF213; BLOC1S2, PCBP4	3.00	3.00	0.46694761
4.11E+03	Cell cycle	RNF213; BLOC1S2; PCBP4	3.00	3.00	0.46694761
5.21E+03	MicroRNAs in cancer	BLOC1S2; RNF213; PCBP4	3.00	3.00	0.46694761
4.06E+03	Chemokine signaling pathway	LOC108634440; ENPP1; LOC108634456	3.00	3.00	0.46694761
5.20E+03	Pathways in cancer	BLOC1S2; MRC1; RNF213	3.00	3.00	0.46694761
4.81E+03	Regulation of actin cytoskeleton	CD55; ENPP5; ENPP1	3.00	3.00	0.46694761
4.15E+03	PI3K-Akt signaling pathway	PCBP4; RNF213; BLOC1S2	3.00	3.00	0.46694761
4.97E+03	Salivary secretion	WIPF1; LAPTM5	2.00	2.00	0.60490941
5.21E+03	Proteoglycans in cancer	BLOC1S2; RNF213	2.00	2.00	0.60490941
4.65E+03	Natural killer cell mediated cytotoxicity	TRIM38; ENPP1	2.00	2.00	0.60490941

5.14E+03	Malaria	SGPP1; LOC108637665	2.00	2.00	0.60490941
4.37E+03	VEGF signaling pathway	ENPP5; KIF21A	2.00	2.00	0.60490941
5.17E+03	Epstein-Barr virus infection	BLOC1S2; RNF213	2.00	2.00	0.60490941
1.23E+03	Biosynthesis of amino acids	LOC102179924; LOC108634443	2.00	2.00	0.60490941
5.22E+03	Melanoma	BLOC1S2; RNF213	2.00	2.00	0.60490941
5.15E+03	Tuberculosis	LOC102180655; SGPP1	2.00	2.00	0.60490941
4.63E+03	Jak-STAT signaling pathway	PCBP4; BLOC1S2	2.00	2.00	0.60490941
5.21E+03	Glioma	RNF213; BLOC1S2	2.00	2.00	0.60490941
5.22E+03	Bladder cancer	BLOC1S2; RNF213	2.00	2.00	0.60490941
4.14E+03	Endocytosis	LIN28A; RNF213	2.00	2.00	0.60490941
5.01E+03	Alzheimer disease	LOC108637665; LOC102179924	2.00	2.00	0.60490941
5.22E+03	Prostate cancer	RNF213; BLOC1S2	2.00	2.00	0.60490941
5.17E+03	Human T-cell leukemia virus 1 infection	BLOC1S2; PCBP4	2.00	2.00	0.60490941
1.20E+03	Carbon metabolism	LOC108634443; LOC102179924	2.00	2.00	0.60490941
5.22E+03	Chronic myeloid leukemia	RNF213; BLOC1S2	2.00	2.00	0.60490941
4.01E+03	ErbB signaling pathway	BLOC1S2; CD55	2.00	2.00	0.60490941
5.41E+03	Hypertrophic cardiomyopathy (HCM)	C5H12orf75	1.00	1.00	0.77966102
1.40E+02	Steroid hormone biosynthesis	SERTAD4	1.00	1.00	0.77966102
5.23E+03	Choline metabolism in cancer	LOC102169861	1.00	1.00	0.77966102
2.60E+02	Glycine, serine and threonine metabolism	LOC108634443	1.00	1.00	0.77966102
4.53E+03	Tight junction	ENPP5	1.00	1.00	0.77966102
5.64E+02	Glycerophospholipid metabolism	LOC102169861	1.00	1.00	0.77966102

2.01E+03	ABC transporters	LOC102168687	1.00	1.00	0.77966102
5.13E+03	Legionellosis	SGPP1	1.00	1.00	0.77966102
4.96E+03	Aldosterone-regulated sodium reabsorption	B2M	1.00	1.00	0.77966102
4.67E+03	Leukocyte transendothelial migration	ENPP1	1.00	1.00	0.77966102
8.60E+02	Porphyrin and chlorophyll metabolism	SAT1	1.00	1.00	0.77966102
3.46E+03	Fanconi anemia pathway	RNF125	1.00	1.00	0.77966102
4.72E+03	Synaptic vesicle cycle	LOC108634238	1.00	1.00	0.77966102
5.16E+03	Influenza A	SGPP1	1.00	1.00	0.77966102
4.07E+03	Phospholipase D signaling pathway	LOC102169861	1.00	1.00	0.77966102
4.26E+03	Adrenergic signaling in cardiomyocytes	C5H12orf75	1.00	1.00	0.77966102
4.67E+03	Fc gamma R-mediated phagocytosis	ENPP1	1.00	1.00	0.77966102
4.08E+03	Neuroactive ligand-receptor interaction	CUX2	1.00	1.00	0.77966102
4.02E+03	cAMP signaling pathway	ENPP1	1.00	1.00	0.77966102
4.55E+03	Signaling pathways regulating pluripotency of stem cells	AMMECR1	1.00	1.00	0.77966102
1.00E+01	Glycolysis / Gluconeogenesis	LOC102179924	1.00	1.00	0.77966102
4.73E+03	GABAergic synapse	CUX2	1.00	1.00	0.77966102
5.03E+03	Morphine addiction	CUX2	1.00	1.00	0.77966102
4.72E+03	Retrograde endocannabinoid signaling	CUX2	1.00	1.00	0.77966102
4.66E+03	B cell receptor signaling pathway	ENPP1	1.00	1.00	0.77966102
5.32E+03	Inflammatory bowel disease (IBD)	SGPP1	1.00	1.00	0.77966102
4.66E+03	T cell receptor signaling pathway	ENPP1	1.00	1.00	0.77966102

5.41E+03	Dilated cardiomyopathy (DCM)	C5H12orf75	1.00	1.00	0.77966102
5.16E+03	Hepatitis B	BLOC1S2	1.00	1.00	0.77966102
7.40E+02	Riboflavin metabolism	SAT1	1.00	1.00	0.77966102
9.10E+02	Nitrogen metabolism	BCL2A1	1.00	1.00	0.77966102
4.62E+03	NOD-like receptor signaling pathway	SGPP1	1.00	1.00	0.77966102
4.92E+03	Oxytocin signaling pathway	BLOC1S2	1.00	1.00	0.77966102
5.03E+03	Nicotine addiction	CUX2	1.00	1.00	0.77966102
4.92E+03	Thyroid hormone signaling pathway	RNF213	1.00	1.00	0.77966102
5.21E+03	Renal cell carcinoma	MRC1	1.00	1.00	0.77966102
4.61E+03	Complement and coagulation cascades	EIF2AK2	1.00	1.00	0.77966102
4.26E+03	Cardiac muscle contraction	C5H12orf75	1.00	1.00	0.77966102
4.14E+03	Protein processing in endoplasmic reticulum	CTSL	1.00	1.00	0.77966102
4.98E+03	Vitamin digestion and absorption	LOC102186003	1.00	1.00	0.77966102
4.62E+03	Cytosolic DNA-sensing pathway	SGPP1	1.00	1.00	0.77966102
4.74E+03	Taste transduction	CUX2	1.00	1.00	0.77966102
3.02E+03	Basal transcription factors	RAB31	1.00	1.00	0.77966102
4.66E+03	Fc epsilon RI signaling pathway	ENPP1	1.00	1.00	0.77966102
5.62E+02	Inositol phosphate metabolism	SRGN	1.00	1.00	0.77966102
5.16E+03	Hepatitis C	BLOC1S2	1.00	1.00	0.77966102
5.16E+03	Measles	PCBP4	1.00	1.00	0.77966102
5.14E+03	African trypanosomiasis	SGPP1	1.00	1.00	0.77966102

4.21E+03	Longevity regulating pathway - multiple species	CTSL	1.00	1.00	0.77966102
5.41E+03	Arrhythmogenic right ventricular cardiomyopathy (ARVC)	HNMT	1.00	1.00	0.77966102
4.07E+03	Phosphatidylinositol signaling system	LOC102169861	1.00	1.00	0.77966102
4.36E+03	Axon guidance	AMMECR1	1.00	1.00	0.77966102
4.35E+03	TGF-beta signaling pathway	AMMECR1	1.00	1.00	0.77966102
5.32E+03	Rheumatoid arthritis	SGPP1	1.00	1.00	0.77966102
4.92E+03	Prolactin signaling pathway	PCBP4	1.00	1.00	0.77966102
5.13E+03	Salmonella infection	SGPP1	1.00	1.00	0.77966102
5.61E+02	Glycerolipid metabolism	LOC102169861, SH3BGRL3	2.00	3.00	0.880833
4.01E+03	MAPK signaling pathway	CD55	1.00	2.00	0.95441262
2.30E+02	Purine metabolism	HEBP1	1.00	2.00	0.95441262
5.10E+01	Fructose and mannose metabolism	SH3BGRL3	1.00	2.00	0.95441262
5.17E+03	Herpes simplex virus 1 infection	TCEAL9	1.00	2.00	0.95441262
4.12E+03	Ubiquitin mediated proteolysis	RNF213	1.00	2.00	0.95441262
4.31E+03	Wnt signaling pathway	PCBP4	1.00	2.00	0.95441262
4.00E+01	Pentose and glucuronate interconversions	SH3BGRL3	1.00	2.00	0.95441262
5.20E+01	Galactose metabolism	SH3BGRL3	1.00	2.00	0.95441262

S gene number: the number of genes annotated with significant differences for a particular GO.

B gene number: annotated as the number of genes in a particular GO.

Supplementary Table S11: The 132 DEGs were screened to build the PPI network in HM and LM groups

Gene-name	log2(fold change)	P value	regulation
ABHD16A	1.380797622	0.019161866	up
AKR1B1	3.547600607	5.95537E-15	up
ANXA1	1.79606113	0.000576989	up
ANXA2	2.313632918	5.40636E-15	up
ANXA3	1.044329344	0.038304758	up
AP1S2	1.248379089	0.014667661	up
ARHGAP1	1.17686422	0.002423263	up
ARHGDIB	1.124282344	0.004804429	up
B2M	1.25966217	0.000402961	up
BIN3	1.06448078	0.002237735	up
BIRC3	1.052444545	0.001045812	up
BTG4	2.221768937	1.11715E-08	up
CAB39L	1.143849172	0.00018705	up
CCNA1	2.403528157	7.86815E-09	up
CD55	1.267350736	0.000768789	up
CDC25B	1.186164003	0.000181093	up
CDKN1A	3.361729806	1.955E-19	up
CKS1B	1.013947042	0.000239968	up
COTL1	1.878629235	3.61311E-10	up
CRIP1	2.997592945	1.72762E-08	up

CRYAB	2.61978515	2.58002E-12	up
CTGF	1.431283971	0.000257834	up
CTSB	1.193887434	0.005120317	up
CTSL	1.529379713	0.000464022	up
CYB5A	1.14595073	0.000921702	up
CYR61	1.241662081	0.000461504	up
DDIT4	1.509467409	0.000310708	up
DEGS2	1.156290484	0.007163958	up
DFFA	1.280122082	0.000156285	up
DYNLRB2	1.857348923	6.46303E-05	up
DYNLT3	1.389273359	8.98098E-05	up
EAF2	1.209276804	0.011278399	up
EBPL	1.24427052	0.019111846	up
ERI3	1.291673939	0.001246467	up
FABP3	1.234891303	0.000310968	up
FABP7	1.14336669	0.003220597	up
FAM81A	1.119275632	0.009566902	up
FAS	1.190279639	0.001969736	up
FLT3	1.985127124	4.14869E-08	up
FOLR1	1.073706874	0.000223191	up
FOSL1	1.726624865	3.01974E-05	up
FXYD4	3.960079139	2.24809E-12	up

FXYD6	1.844753646	4.03911E-07	up
GADD45A	1.257952036	3.87469E-06	up
GALM	1.251431146	1.6776E-05	up
GNAI2	1.022035109	0.024006105	up
GNGT1	1.917917389	0.000114613	up
GNGT2	1.125888452	0.003272974	up
GPR84	2.296501233	1.43715E-05	up
GSS	1.283777843	6.30913E-05	up
GTF2H5	1.026420223	0.000327715	up
GUCA1A	1.220599012	2.93684E-06	up
GUCA2A	1.442740981	0.000291325	up
HAND1	1.395849349	0.001647692	up
HBEGF	1.025571807	0.021533563	up
HEBP1	1.463928533	0.006073544	up
HK1	1.40427717	1.65309E-05	up
HMGCR	1.099780545	5.16052E-05	up
HMMR	1.066500554	0.000592955	up
HSPB1	2.410502697	1.23819E-07	up
HSPB11	1.262948417	0.01194429	up
ID2	1.003485121	0.000736622	up
IER3	1.487174137	2.97476E-06	up
IL6	1.233652946	0.00021985	up

KPNA7	1.028505639	0.000687814	up
KRT18	1.421445426	0.000367307	up
KRT8	1.127531304	0.001267235	up
LAMC1	1.254820406	1.05311E-06	up
LGALS7	2.602557756	7.70799E-05	up
LITAF	1.053317897	8.2634E-05	up
LRP2	1.071639628	0.00227762	up
LY6G6C	2.837777922	6.46312E-08	up
MAP1LC3A	1.54137664	2.98301E-05	up
MDM2	1.635340914	4.15827E-10	up
MIOX	1.264245174	0.012437421	up
MLLT11	2.148712024	2.70735E-10	up
MSMO1	1.076267219	0.000565075	up
MYO1B	1.286089383	0.000347432	up
MYO1E	1.182068811	0.000420285	up
MYOF	1.537003284	1.61125E-06	up
NDRG1	1.820585306	2.37481E-08	up
NPPB	1.659499816	0.003952403	up
NPTN	1.151738302	0.00021522	up
NTAN1	1.283887078	8.12141E-06	up
NUDT22	1.416321379	0.001718778	up
PAIP1	1.009212948	0.000159011	up

PDCD4	1.058697527	0.004820393	up
PDK3	1.156704308	0.004218963	up
PDPN	1.70167568	9.34894E-07	up
PEA15	1.397150197	0.000149361	up
PERP	1.2304598	1.97726E-05	up
PHLDA3	1.664346069	3.13233E-09	up
PKM	1.126208581	0.002900826	up
PLAC8	1.882097563	1.15665E-06	up
PLK2	1.143579247	0.001485276	up
PPM1D	1.662521992	1.2695E-05	up
PPP1R1A	1.161323669	0.027434094	up
PPP2R5A	1.046760914	0.006114061	up
PRDX1	1.05700355	6.42919E-05	up
PRDX6	1.008822576	0.000768335	up
PRSS23	1.072789865	0.004686173	up
RLIM	1.366710912	2.24788E-05	up
S100A10	1.653621793	3.43284E-08	up
S100A11	2.085574851	1.77985E-07	up
S100A2	2.422201208	3.67125E-08	up
S100A4	1.92227043	5.62808E-05	up
S100A5	2.31555433	2.66252E-07	up
SAT1	1.448923335	0.002906342	up

SH3BGRL3	1.698840048	8.16669E-06	up
SIAH1	1.785229414	1.39985E-06	up
SLC20A1	1.000131911	0.000228072	up
SLC30A1	1.392108589	9.07981E-05	up
SLC39A10	1.029973074	0.007534889	up
SLC39A2	1.000801251	0.012607901	up
SLC39A8	1.03879971	0.004205339	up
SMPD3	1.121769466	0.001277162	up
SPARC	1.131273039	0.000495327	up
SPOPL	1.251311504	0.000197247	up
STON1	1.134834173	0.001092738	up
SUPT4H1	1.040337949	0.000126871	up
TACSTD2	1.587461731	1.94262E-07	up
TIMP1	1.551534133	5.03432E-06	up
TMEM65	1.046676538	0.003183872	up
TNNI3	1.312987917	0.006169044	up
TP53INP1	1.138409162	0.000179535	up
TPD52	1.362270114	0.015107468	up
TPM2	3.100288005	1.43385E-11	up
TRIM59	1.578654009	0.000327422	up
TSTD1	1.045048176	0.018432305	up
UGP2	1.124706859	1.8164E-05	up

VAMP5	1.901928603	0.000536372	up
WEE1	1.264950043	0.000114834	up

Supplementary Table S12: The 22 hub genes were identified

Gene symbol	log2(fold change)	P value	regulation	Functional analysis
FAS	1.190280	1.97E-03	up	p53 signaling pathway, MAPK signaling pathway, Pathways in cancer, Apoptosis, Proteoglycans in cancer
GDPD1	1.586196	2.37E-05	up	VEGF signaling pathway, GnRH signaling pathway, mTOR signaling pathway, TGF-beta signaling pathway, MAPK signaling pathway, FoxO signaling pathway, NOD-like receptor signaling pathway
GADD45A	1.260000	3.87E-06	up	Apoptosis, p53 signaling pathway, MAPK signaling pathway, FoxO signaling pathway, Cell cycle
ZBTB33	1.188774	1.35E-03	up	PI3K-Akt signaling pathway, Rap1 signaling pathway, MAPK signaling pathway, Ras signaling pathway
CREB3	1.067456	1.74E-02	up	Adrenergic signaling in cardiomyocytes, PI3K-Akt signaling pathway, Thyroid hormone synthesis, Estrogen signaling pathway
MDM2	1.635341	4.16E-10	up	PI3K-Akt signaling pathway, Cell cycle, p53 signaling pathway, FoxO signaling pathway
CDKN1A	3.361730	1.95E-19	up	FoxO signaling pathway, HIF-1 signaling pathway, Cell cycle, p53 signaling pathway, Pathways in cancer, PI3K-Akt signaling pathway, Epstein-Barr virus infection
CDC25B	1.186164	1.81E-04	up	Cell cycle, MAPK signaling pathway, MicroRNAs in cancer
IL6	2.200000	2.20E-04	up	Intestinal immune network for IgA production, Jak-STAT signaling pathway, Toll-like receptor signaling pathway, AGE-RAGE signaling pathway in diabetic complications
HSP70.1	2.295334	1.79E-05	up	Endocytosis，Protein processing in endoplasmic reticulum，MAPK signaling pathway
HSPB1	2.410000	1.24E-07	up	MAPK signaling pathway, VEGF signaling pathway, Amoebiasis

FOXO1	1.223448	2.15E-04	up	AMPK signaling pathway, Foxo signaling pathway, Pathways in cancer, AGE-RAGE signaling pathway in diabetic complications
FABP3	1.234891	3.11E-04	up	PPAR signaling pathway
PPP1R1A	1.610000	2.70E-02	up	Adrenergic signaling in cardiomyocytes, Intestinal immune network for IgA production
GPX6	1.674210	1.66E-02	up	Arachidonic acid metabolism, Glutathione metabolism, Thyroid hormone synthesis
MAPK13	1.890000	8.71E-05	up	Signaling pathways regulating pluripotency of stem cells, MAPK signaling pathway, Rap1 signaling pathway
PLK2	1.143579	1.49E-03	up	Foxo signaling pathway
WEE1	1.264950	1.15E-04	up	Cell cycle
CKS1B	1.013947	2.40E-04	up	Pathways in cancer, Small cell lung cancer
CCNA1	2.403528	7.87E-09	up	Cell cycle, AMPK signaling pathway, Epstein-Barr virus infection
CDC25B	1.186164	1.81E-04	up	Cell cycle, MAPK signaling pathway, MicroRNAs in cancer
CDKN1A	3.361730	1.95E-19	up	Cell cycle, Foxo signaling pathway, p53 signaling pathway, Pathways in cancer, PI3K-Akt signaling pathway, Prostate cancer
